# Effects of exercise on depression in adults with arthritis: a systematic review with meta-analysis of randomized controlled trials

**DOI:** 10.1186/s13075-015-0533-5

**Published:** 2015-02-03

**Authors:** George A Kelley, Kristi S Kelley, Jennifer M Hootman

**Affiliations:** Department of Biostatistics, Robert C. Byrd Health Sciences Center, School of Public Health, West Virginia University, Morgantown, WV 26506-9190 USA; Division of Population Health MS F-78, National Center for Chronic Disease Prevention and Health Promotion, Centers for Disease Control and Prevention Atlanta, Atlanta, GA 30341 USA

## Abstract

**Introduction:**

Previous randomized controlled trials have led to conflicting findings regarding the effects of exercise on depressive symptoms in adults with arthritis and other rheumatic conditions (AORC). The purpose of this study was to use the meta-analytic approach to resolve these discrepancies.

**Methods:**

The inclusion criteria were: (1) randomized controlled trials, (2) exercise (aerobic, strength training, or both) ≥4 weeks, (3) comparative control group, (4) adults with osteoarthritis, rheumatoid arthritis, fibromyalgia or systemic lupus erythematosus, (5) published studies in any language since January 1, 1981 and (6) depressive symptoms assessed. Studies were located by searching 10 electronic databases, cross-referencing, hand searching and expert review. Dual-selection of studies and data abstraction was performed. Hedge’s standardized mean difference effect size (g) was calculated for each result and pooled using random-effects models, an approach that accounts for heterogeneity. Non-overlapping 95% confidence intervals (CI) were considered statistically significant. Heterogeneity based on fixed-effect models was estimated using Q and *I*^*2*^ with alpha values ≤0.10 for Q considered statistically significant.

**Results:**

Of the 500 citations reviewed, 2,449 participants (1,470 exercise, 979 control) nested within 29 studies were included. Length of training, reported as mean ± standard deviation (±SD) was 19 ± 16 weeks, frequency 4 ± 2 times per week and duration 34 ± 17 minutes per session. Overall, statistically significant exercise minus control group reductions were found for depressive symptoms (g = −0.42, 95% CI, −0.58, −0.26, Q = 126.9, *P* <0.0001, *I*^*2*^ = 73.2%). The number needed-to-treat was 7 (95% CI, 6 to 11) with an estimated 3.1 million (95% CI, 2.0 to 3.7) United States adults not currently meeting physical activity guidelines improving their depressive symptoms if they began and maintained a regular exercise program. Using Cohen’s U_3_ Index, the percentile reduction was 16.4% (95% CI, 10.4% to 21.9%). All studies were considered to be at high risk of bias with respect to blinding of participants and personnel to group assignment.

**Conclusions:**

Exercise is associated with reductions in depressive symptoms among selected adults with AORC. A need exists for additional, well-designed and reported studies on this topic.

**Electronic supplementary material:**

The online version of this article (doi:10.1186/s13075-015-0533-5) contains supplementary material, which is available to authorized users.

## Introduction

Arthritis is a major public health problem among adults in the United States (US). Current US estimates place the prevalence of doctor-diagnosed arthritis at 55.2 million (22.7%) adults [1], and is projected to increase to 67 million (25%) adults by the year 2030 [2]. Not surprisingly, the costs associated with arthritis and other rheumatic conditions are substantial. In 2003, the total costs associated with arthritis were estimated at $127.8 billion, $80.8 billion in direct costs and $47.0 billion in indirect costs [3]. A common mental health problem among adults with arthritis is depression. For example, a recent study of 1,793 US adults 45 years of age and older with arthritis found that 18% had depression while only 51% sought help for their depression [4]. One potential non-pharmacologic intervention for reducing depressive symptoms in adults with arthritis is exercise. Unfortunately, the prevalence of exercise in adults with arthritis is low. For example, the percentage of adults with doctor-diagnosed arthritis who perform moderate physical activity for at least 30 minutes per day, three days per week, has been reported to be only 37% [5]. In addition, previous randomized controlled trials that examined the effects of exercise (aerobic, strength training, or both) on depressive symptoms in adults with arthritis and other rheumatic conditions (AORC) have led to conflicting results [6-34] with 23 and 36 exercise versus control between-group differences reported as either statistically significant [12-14,18,19,21,23,25,27-30,32,33] or null [6-11,15-18,20,22-24,26,31,34], respectively. While this may lead one to generally conclude that exercise does little to reduce depressive symptoms in adults with AORC, this would be shortsighted since it relies on the vote-counting approach [35], an approach that has been shown to be less valid than the meta-analytic approach [35]. Recently, two members of the investigative team (GAK and KSK) conducted a systematic review of previous meta-analyses addressing the effects of exercise on depressive symptoms in adults with AORC [36]. Only two previous meta-analyses, limited to adults with fibromyalgia [37,38], met the criteria for inclusion [36]. Exercise minus control group reductions in depressive symptoms were found for both meta-analyses (standardized mean difference (SMD), −0.61, 95% confidence interval (CI), −0.99 to −0.23, *P* = 0.002; SMD, −0.32, 95% CI, −0.53 to −0.12, *P* = 0.002 [36]. Another meta-analysis that included participants with a variety of chronic illnesses and which was excluded from our previous review [36] found a SMD reduction of −0.29 (95% CI, −0.16 to −0.43) in depressive symptoms among fibromyalgia participants as well as a reduction of −0.23 (95% CI, −0.11 to −0.34) in participants with chronic pain other than fibromyalgia [39]. However, these latter findings included participants with conditions other than arthritis, for example back pain. In addition, while this previous meta-analysis included an analysis for such things as small-study effects, number-needed-to treat and meta-regression, these analyses were conducted across all chronic illnesses versus those with AORC [39]. While these previous findings are encouraging, no meta-analysis focused specifically on the effects of exercise on depressive symptoms in adults with other AORC (osteoarthritis, rheumatoid arthritis, systemic lupus erythematous), met the eligibility criteria. Since the effects of exercise on depressive symptoms may vary across different AORC, the inclusion of such populations in a meta-analysis is important. In addition, the most recent meta-analysis of the two [38] was limited to studies published up until April 2009, suggesting the need for a more up-to-date review on the topic. Thus, the purpose of the current study was to conduct a systematic review with meta-analysis to determine the effects of exercise (aerobic, strength training, or both) on depressive symptoms in adults with AORC.

## Methods

### Study eligibility criteria

The *a priori* inclusion criteria for this meta-analysis were as follows: (1) randomized controlled trials with the unit of assignment at the participant level, (2) exercise-only intervention group (aerobic, strength training, or both), (3) community-deliverable exercise interventions ≥4 weeks in duration, (4) comparative control group (non-intervention, usual care, wait-list control, attention control), (5) adults 18-years old and older with one of the following: rheumatoid arthritis, osteoarthritis, or fibromyalgia, (6) studies published in any language between 1 January 1981 and 1 January 2013, (7) depressive symptoms as an outcome. *Post hoc*, a decision was made to include studies in adults with systemic lupus erythematosus. For this proposed project, community-deliverable exercise interventions were defined as those that could be performed, or had the potential to be adapted and performed, by persons in a community setting (recreation or senior centers, in the home or neighborhood, etc.) and meet the implementation guidelines for physical activity interventions recommended by the Arthritis Program at the Centers for Disease Control and Prevention [40]. This includes exercise in a pool [40]. An exercise duration of at least four weeks was chosen based on previous meta-analytic research that included physical activity regimens of as little as four weeks and in which depressive symptoms were reduced in the general adult population [41]. Studies were limited to full articles published in peer-reviewed journals and examined for potential publication bias based on recent recommendations (see Statistical Analysis section for description) [42]. Unpublished work, defined as master’s theses, dissertations, abstracts from conference proceedings, technical reports and studies filed in an investigator’s drawer, was not included. The rationale for this decision was based on the work of van Driel *et al*. [43], who concluded that: (1) the difficulty in retrieving unpublished work could lead to selection bias, (2) many unpublished trials are eventually published, (3) the methodological quality of such studies is poorer than those that are published, and (4) the effort and resources required to obtain unpublished work may not be warranted [43]. This approach is consistent with recent practice [43]. The year 1981 was chosen as the starting point for study searches based on a preliminary search in PubMed in which the first cited randomized controlled trial on exercise and arthritis in adults was published in 1981 [44].

### Data sources

Studies were retrieved using the following 10 electronic databases: (1) Medline, (2) CINAHL, (3) Sport Discus, (4) PsycINFO, (5) Scopus, (6) Academic Search Complete, (7) Proquest, (8) Cochrane Central Register of Controlled Trials, (9) PEDro and (10) Web of Science. All electronic searches were conducted by a Health Sciences librarian (JS) with assistance from the first and second authors. While the search strategies used varied according to the requirements of the different databases searched, keywords centered on the terms ‘exercise’ , ‘arthritis’ and ‘depression’. An example of the search strategy for one database (Scopus) can be found in Additional file 1. After removing duplicates and completing the study selection process, the overall precision of the searches was calculated by dividing the number of studies included by the total number of studies screened [45]. The number needed to read (NNR) was then calculated as the inverse of the precision [45]. In addition to electronic database searches, cross-referencing for potentially eligible meta-analyses from retrieved reviews was also conducted as well as expert review. All studies were stored in Reference Manager, version 12.0.1 [46].

### Study selection

All studies were selected by the first two authors, independent of each other. Disagreements regarding the final list of studies to be included were resolved by consensus. Multiple publication bias was addressed by only including one set of data on the same subjects. All included studies, as well as a list of excluded studies, including reasons for exclusion, were stored in Reference Manager (version 12.0.1) [46].

### Data abstraction

Prior to data abstraction, a detailed codebook that could hold up to 260 items per study was developed by all three members of the research team in Microsoft Excel 2007 [47]. The major categories of variables that were coded included: (1) study characteristics, (2) subject characteristics, (3) exercise program characteristics, (4) primary outcomes and (5) secondary outcomes. The primary outcome for this study, established *a priori*, was changes in depressive symptoms. Secondary outcomes included the following variables: body weight, body mass index (BMI) in kg^.^m^2^, percent body fat, physical function, pain (global), quality of life (overall score), anxiety, aerobic fitness (VO_2max_ in ml^.^kg^-1.^min^−1^), muscular strength (upper and lower body) and balance (overall, dynamic or static). Secondary outcomes were only included if data for depressive symptoms were available. Our rationale for including these secondary outcomes was based on their potential impact on depressive symptoms as well as the fact that they are often at less than optimal levels in adults with AORC. All studies were coded by the first two authors, independent of each other. They then met and reviewed every entry (22,136 total) for accuracy and consistency. Discrepancies were resolved by consensus. If consensus could not be reached, the third author served as an arbitrator. Using Cohen’s kappa statistic [48], the overall agreement rate prior to correcting discrepant items ranged from 0.70 to 0.98 $\left(\overline{x}\pm SD = 0.89\pm 0.07,\ \mathrm{M}\mathrm{d}\mathrm{n} = 0.90\right)$.

### Risk of bias

The Cochrane Collaboration risk of bias instrument was used to assess bias across six domains: (1) random sequence generation, (2) allocation concealment, (3) blinding of participants and personnel, (4) blinding of outcome assessment, (5) incomplete outcome data, (6) selective reporting and (7) whether the participants were physically inactive, as defined by the original study authors, prior to taking part in the study [49]. Each item was classified as having a high, low, or unclear risk of bias [49]. Assessment for risk of bias was limited to the primary outcome of interest, changes in depressive symptoms. Since it is impossible to blind participants to group assignment in exercise intervention protocols, all studies were considered to be at a high risk of bias with respect to blinding of participants and personnel. Based on previous research, no study was excluded based on the results of the risk of bias assessment [50]. All assessments were performed by the first two authors, independent of each other. Both authors then met and reviewed every item (203 total) for agreement. Disagreements were resolved by consensus. Using Cohen’s kappa statistic [48], the overall agreement rate prior to correcting discrepant items ranged from 0.14 to 0.71 $\left(\overline{x}\pm SD = 0.63\pm 0.32,\ \mathrm{M}\mathrm{d}\mathrm{n} = 0.71\right)$.

### Statistical analysis

The *a priori* plan was to conduct a one-step individual participant data (IPD) meta-analysis [51]. However, because of: (1) the inability to obtain IPD from all eligible studies, (2) the inability to resolve discrepancies between the IPD provided and data reported in the published studies, for example, final sample sizes and (3) the potential loss of power with fewer included studies at the IPD level, a *post hoc* decision was made to conduct an aggregate data meta-analysis, an approach similar to conducting a two-step meta-analysis with IPD [51].

#### Calculation of effect sizes for primary and secondary outcomes from each study

The primary outcome for this study was depressive symptoms, calculated as the SMD effect size *g*. This was accomplished by subtracting the change score difference in the exercise group from the change score difference in the control group. Variances were calculated from the pooled standard deviations of change scores in the intervention and control groups. If change score standard deviations were not available, these were calculated from reported 95% CIs, pre and post standard deviation (SD) values according to procedures developed by Follmann *et al*. [52], or other traditional methods (for example, *t*-tests, exact probability values). Each *g* was then weighted by the inverse of its variance and adjusted for small sample bias [53]. The beneficial effects of exercise on depressive symptoms were denoted by a negative *g*. Studies that used assessment instruments in which a positive *g* represented reductions in depressive symptoms were reverse scaled so that negative values were indicative of improvements. In order to try to maintain independence as well as the fact that no one most valid and reliable measure for assessing depressive symptoms in adults with AORC exists, overall results were pooled for those studies that assessed depressive symptoms using more than one assessment instrument. The same approach was used for studies that reported results based on both intention-to-treat and per-protocol analyses. Effect sizes for secondary outcomes were calculated using either the original metric, for example body weight in kilograms, or *g* given the different assessment instruments used for many of the included outcomes (anxiety, pain, and etc.). For all secondary outcomes the beneficial direction of effect reported was the natural direction of benefit, for example, negative values for decreases in anxiety, positive values for increases in quality-of-life. Where necessary, values were reverse-scaled. Similar to depressive symptoms, results were pooled for those studies that assessed any secondary outcomes using more than one assessment instrument and/or analyzed data using both the intention-to-treat and per-protocol approach.

#### Pooled estimates for primary and secondary outcomes

Random-effects, method-of-moments models that incorporate heterogeneity into the overall estimate were used to pool both primary and secondary outcomes from each study [54]. Multiple groups from the same study, for example aerobic and strength training groups, were analyzed independently as well as collapsing multiple groups so that only one result represented each outcome from each study. The rationale for collapsing multiple groups so that only one effect size represented each outcome from each study was based on the tendency for results from multiple groups in the same study to be correlated, the result being a loss of statistical independence. This study-level analysis was limited to overall findings only. All other analyses (influence analysis, cumulative meta-analysis, moderator analysis, simple meta-regression, etc.) were conducted with group level data. While results were pooled for those studies in which the same outcome was measured using more than one assessment instrument and/or results were reported using both intention-to-treat and per-protocol approaches, separate moderator analyses were also conducted for each assessment type and type of analysis. Non-overlapping 95% CI were considered statistically significant. To enhance practical application, the number-needed-to treat (NNT) was calculated for any overall findings that were reported as statistically significant. This was accomplished using the approach suggested by the Cochrane Collaboration and assuming a control group risk of 30% [55]. Briefly, based on recommendations of the Cochrane Collaboration [55], we converted the standardized mean difference into a natural log odds ratio, odds ratio, assumed control risk, based on 30%, and finally, the NNT. The 30% control group risk was based on a previous review by Sonawalla and Rosenbaum [56] in which it was reported that mean placebo response rates in antidepressant clinical trials were 30% to 40%. Based on the NNT for changes in depressive symptoms, gross estimates of the number of adults with AORC in the US who could benefit from exercise but were not meeting current exercise guidelines were calculated. This was based on an estimated 34.8 million US adults with doctor-diagnosed arthritis, derived by multiplying the number of adults with doctor-diagnosed arthritis (55.2 million) [1] by the percentage of adults with arthritis who were not currently meeting physical activity guidelines (63%) [5]. Practical application was further enhanced by calculating Cohen’s U_3_ index, an index used to determine the percentile gain in an intervention group [57]. For example, a g of 0.40 suggests that, on average, a person in the exercise group would be at approximately the 66th percentile in terms of improving their depressive symptoms. This translates into being approximately 16 percentiles higher than the control group [58].

#### Stability and validity of changes in primary and secondary outcomes

Heterogeneity of results between studies was examined using Q as well as an extension of the Q statistic, *I*^*2*^ [59]. Statistical significance for Q was set at an alpha value of ≤0.10. For *I*^*2*^_,_ values of <25%, 25% to <50%, 50% to <75% and 75% or greater were considered to represent very low, low, moderate and large amounts of inconsistency, respectively [59]. To determine treatment effects in a new trial, 95% prediction intervals (PI) were also calculated [60,61]. Small-study effects (publication bias, and so on) were examined qualitatively and quantitatively based on recent recommendations [42]. This included funnel plots as well as the regression approach of Egger *et al*. [42,62]. Non-overlapping 95% CIs for Egger’s regression test for the intercept (β_0_) were considered to be indicative of small-study effects. Outliers were considered to be individual study results in which their 95% CI did not overlap with the 95% CI from pooled results. In order to examine the effects of each result from each study on the overall findings, results were analyzed with each study deleted from the model once. Cumulative meta-analysis, ranked by year, was used to examine the accumulation of evidence over time [63]. Cumulative meta-analysis is an approach where studies are added one at a time and the results summarized as each new study is added. This allows one to visually examine how results have accumulated, and possibly changed, over time [63].

#### Moderator analysis for depressive symptoms

Within and between-group differences in depressive symptoms for categorical variables were examined using mixed effects models that consisted of a random-effects model for combining studies within each subgroup and a fixed effect-model across subgroups [64]. Between-study variance (tau-squared) was considered to be unequal for all subgroups. This value was computed within subgroups but not pooled across subgroups. Categorical analyses included: (1) study characteristics (country, type of control group, whether IPD was provided, random sequence generation, allocation concealment, blinding of participants and personnel, blinding of outcome assessor, incomplete data, selective outcome reporting, whether subjects were physically inactive prior to enrollment as defined by the original study authors, type of analysis performed, provision of sample-size estimates, whether the study was funded, method to assess depression), (2) participant characteristics (adverse events, sex, race/ethnicity, cigarette smoking, whether participants were overweight and/or obese (BMI ≥25 kg/m^2^), type of AORC, medications taken for AORC) and (3) exercise intervention characteristics (type, intensity, delivery). Intensity for aerobic exercise was categorized as low (<40% of heart rate/VO_2_ reserve or <55% of maximal heart rate), moderate (between 40% and 59% of heart rate/VO_2_ reserve or 55% and 69% of maximal heart rate) or high (>59% of heart rate/VO_2_ reserve or >69% of maximal heart rate) [65]. Intensity for strengthening exercise was categorized as low (<50% of 1-repetition maximum), moderate (between 50% and 69% of 1-repetition maximum) or high (>69% of 1-repetition maximum for strength exercise) [65]. *Post hoc*, type of exercise was also examined by separating out tai chi and qi gong from combined aerobic and strength training. The rationale for this was based on the fact that both tai chi and qi gong are considered to be meditative movement therapies which include a mental component that is not traditionally included in typical aerobic and strength training interventions. Non-overlapping 95% CI for both within and between-group analyses were considered statistically significant. All moderator analyses were considered exploratory [66].

#### Meta-regression for changes in depressive symptoms and potential covariates

Simple mixed-effects, method of moments meta-regression was used to examine the potential association between changes in depressive symptoms and continuous variables [64]. Because missing data for different variables from different studies was expected, only simple meta-regression was planned and performed. Potential predictor variables included: (1) study characteristics (year of publication, percentage of dropouts), (2) participant characteristics (age, symptom duration, diagnosis duration, changes in body weight, BMI in kg^.^m^2^, percent body fat, physical function, pain, quality of life, anxiety, maximum oxygen consumption, expressed as VO_2max_ in ml^.^kg^-1.^min^−1^, upper and lower body strength, balance) and (3) exercise intervention characteristics (length, frequency, duration of training, compliance, total minutes per week, calculated as frequency x duration, total minutes per week, adjusted for percent compliance, total minutes of training for the entire intervention period, calculated as length x frequency x duration, and total minutes of training, adjusted for compliance). Non-overlapping 95% CI for the slope (β_1_) were considered statistically significant. Because this was a meta-analysis, all meta-regression analyses were considered exploratory [66].

#### Software used for statistical analysis

Analyses were carried out using Comprehensive Meta-Analysis (version 2.2) [67], Microsoft Excel 2010 [68], and two external add-ins for Excel, SSC-Stat (version 2.18) [69] and EZ-Analyze (version 3.0) [70].

#### Reporting metrics

Selected data are reported as mean ± standard deviation $\left(\overline{x}\pm SD\right)$ and median (Mdn).

## Results

### Study characteristics

**Figure 1:**
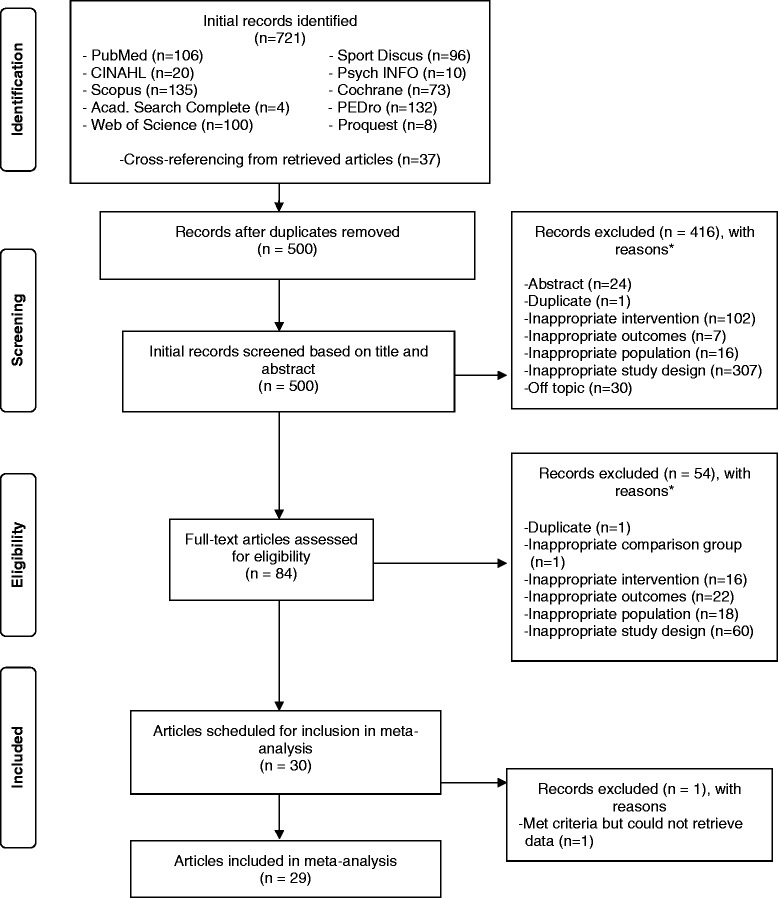
**Flow diagram for the selection of studies.** *, number of reasons exceeds the number of studies because some studies were excluded for more than one reason.

A general description of the characteristics of each study is shown in Table 1. Of the 500 citations reviewed, a total of 2,449 participants (1,470 exercise, 979 control) nested within 64 groups (35 exercise, 29 control) and 29 studies were included in the final analysis [6-34]. The precision of the searches was 0.06 while the NNR was 17. A description of the search process, including the reasons for excluded studies, is shown in Figure 1 while a list of each excluded study, including the reason(s) for exclusion, is shown in Additional file 2. The number of exercise groups exceeded the number of studies because six studies included more than one group [11,19,20,23,24,26]. The number of subjects in each group from each study varied widely, ranging from 8 to 149 in the exercise groups $\left(\overline{x}\pm SD=42\pm 35,\;\mathrm{M}\mathrm{d}\mathrm{n}=28\right)$ and 8 to 144 in the control groups $\left(\overline{x}\pm SD=34\pm 30,\;\mathrm{M}\mathrm{d}\mathrm{n}=27\right)$. Twenty-eight studies were published in English language journals [6-27,29-34] and one in Spanish [28]. Studies were conducted in eleven different countries; thirteen in the United States [7-10,16,17,19,20,22-24,32,33], two each in either Brazil [15,30], Canada [12,26], Finland [14,31], Portugal [28,29], Spain [6,25] or Sweden [13,18], and one each in either Australia [11], Norway [34], Turkey [27], or the United Kingdom [21]. For types of controls, eight appeared to use a non-intervention control group [6,12,14,17,20,21,29,31], six used an attention control group [7,8,10,16,32,33], four used a wait-list control [9,11,13,22], three used usual care [25,28,34], and eight were control groups which could not be definitively categorized [15,18,19,23,24,26,27,30]. Prior to randomization, nine studies matched participants according to either sex [12,20,22], age and sex [21], age, sex and BMI [16], age and duration of symptoms [18], disease status [8], diagnosis of rheumatoid arthritis or osteoarthritis [19], or score on the Fibromyalgia Impact Questionnaire [24]. None of the studies used a crossover design. For data analysis, 19 studies reported using the per-protocol approach only [6-10,13-22,27-30], five used the intention-to-treat approach [11,23,25,32,33] and four used both [12,24,26,34]. One other study essentially used an intention-to-treat approach because they reported no dropouts [31]. Sample size justification was provided by 14 (48.3%) of the studies [8,10,11,16,20-22,24-26,28,30,32,33] while 23 (79.3%) reported receiving some type of funding to conduct their investigation [7-12,14,16-24,26,29-34]. Only seven studies (24.1%) provided IPD [7,9,11,20,26,27,31]. Dropout data, available for 32 (91.4%) of the exercise groups ranged from 0% to 50% ($\overline{x}$ ± SD = 16.2% ± 12.2%, Mdn = 15.3%) while control group dropouts ranged from 0% to 46% ($\overline{x}$ ± SD = 14.1% ± 13.3%, Mdn = 12.5%) for the 25 (86.2%) groups in which data were available. Reasons for participants dropping out or for the investigative team to drop participants from their study included the following: (1) personal reasons, for example, family or job issues, moved, (2) travel issues, (3) schedule conflicts, (4) health issues, (5) time, (6) not attending the requisite number of exercise sessions, (7) lost to follow-up, (8) began taking medications not allowed during the intervention, (9) pain when exercising, (10) began other exercise, (11) identification of a co-morbidity during the intervention, (12) unhappy or unwilling to accept group assignment, (13) exercise too time consuming or boring, (14) increased pain, (15) not enough room or privacy to perform exercises, (16) drug intolerance, (17) incomplete assessments, (18) lost contact with participant, (19) initiated additional treatments and (20) changed medications.

**Table 1:** **General characteristics of studies**

**Study**	**Country**	**Participants**	**Exercise intervention**	**Depression assessment**
Alentorn-Geli *et al*. [6]	Spain	24 women with FM randomly assigned to either an exercise (n = 12, $\overline{x}$ ± SD = 53.7 ± 9.4 yrs) or control group (n = 12, age, $\overline{x}$ ± SD = 59.3 ± 7.3 yrs)	2 x/wk, 90 min/d (aerobic, 30 min walking, 65-85% MHR, or salsa dancing: 2, 15/min sessions plus stretching and relaxation exercises) for 6 wks	FIQ (depression)
Buckelew *et al*. [7]	United States	60 men and women with FM assigned to either an exercise (n = 30, age $\overline{x}$ ± SD = 45.6 ± 9.4 yrs) or attention control group (n = 30, age, $\overline{x}$ ± SD = 44.3 ± 11.2 yrs)	1-3 x/wk, 1.5-3 hr/session, ROM exercises, strengthening exercises, and low-moderate intensity aerobic exercise (walking at 60-70% MHR) for 6 wks	CES-D
Daltroy *et al*. [8]	United States	71 men and women with either RA or SLE assigned to either an exercise (n = 35, age $\overline{x}$ ± SD = 38.4 ± 7.5 yrs, range 18–50 yrs) or an attention control group (n = 36, age, $\overline{x}$ ± SD = 35.7 ± 7.1 yrs, range 18–50 yrs)	2.2 x/wk, 30 min/session, home-based, unsupervised aerobic exercise (walk, jog, cycle, swim) at 60-80% MHR for 24 wks	CES-D
Etnier *et al*., [9]	United States	16 women (age $\overline{x}$ ± SD = 55.1 ± 8.9 yrs, range 32–70 yrs) with FM assigned to an exercise (n = 8) or wait-list control group (n = 8)	3 d/wk, 60 min/session: walk, 15 min, 55-65% MHR, 8-station light resistance exercise circuit, static-bridging exercises, stretching, for 18 wks	CES-D
Fontaine *et al*., [10]	United States	84 men and women with FM assigned to a lifestyle physical activity (n = 46, age, $\overline{x}$ ± SD = 46.4 ± 11.6 yrs) or control group (n = 38, age, $\overline{x}$ ± SD = 49 ± 10.2 yrs)	5-7 d/wk, accumulate 30 min of moderate-intensity physical activity in short bouts throughout the day by walking, garden/outdoor, household or sports activity for 12 wks	CES-D
Fransen *et al*., [11]	Australia	152 men and women with OA of the hip or knee assigned to a hydrotherapy (n = 55, age, $\overline{x}$ ± SD = 70 ± 6.3 yrs), tai chi (n = 56, $\overline{x}$ ± SD = 70.8 ± 6.3 yrs), or control group (n = 41, age, $\overline{x}$ ± SD = 69.6 ± 6.1 yrs)	2 x/wk, 1 hr/session, of either Tai Chi (for Arthritis, 24 modified forms of Sun style) or hydrotherapy exercises in warm water, for 12 wks	DASS-21
Gowans *et al*., [12]	Canada	57 men and women with FM assigned to an exercise (n = 30, age, $\overline{x}$ ± SD = 44.6 ± 8.7 yrs), or control group (n = 27, age, $\overline{x}$ ± SD = 49.8 ± 7.3 yrs)	3 d/wk, 30 min/session, aerobic exercise (2 walking/jogging classes in a gym, 1 pool class) at 60-75%MHR, for 23 wks	BDI, MHI (depression)
Haak and Scott, [13]	Sweden	57 women with FM assigned to an intervention (n = 29, $\overline{x}$ ± SD = 54 ± 9.4 yrs of age) or wait-list control group (n = 28, $\overline{x}$ ± SD = 53.4 ± 8 yrs of age)	qigong, 9 group sessions over 7 weeks (11.5 hours total), 2 × per day at home for 20 minutes each	BDI
Hakkinen *et al*., [14]	Finland	21 premenopausal women with FM assigned to a progressive strength training (n = 11, $\overline{x}$ ± SD = 39 ± 6 yrs) or control group (n = 10, $\overline{x}$ ± SD = 37 ± 5 yrs)	2 days/wk of supervised progressive strength training, 6–8 exercises, 8–20 reps, 40-80% 1RM, for 21 weeks	BDI
Ide *et al*., [15]	Brazil	40 women with FM assigned to either an exercise (n = 20, age, $\overline{x}$ ± SD = 46.6 ± 9.8 yrs) or control group (n = 20, age, $\overline{x}$ ± SD = 45.5 ± 8.65 yrs)	4 d/wk, 1 hr/session, aquatic respiratory exercise-based program in warm water, for 4 wks	FIQ (depression)
Jones *et al*., [16]	United States	101 men and women with FM assigned to a placebo and exercise (n = 47, $\overline{x}$ ± SD = 49.6 ± 7.7 yrs of age) or attention control: placebo plus diet recall group (n = 54, $\overline{x}$ ± SD = 49.8 ± 7.9 yrs of age)	3 x/wk, 60 min/session, low-impact, nonrepetitive cardioaerobics (30 min), strength training (10 min), flexibility training (5 min), balance training (5 min), and relaxation (10 min) at 40-50% MHR or Borg RPE scale 10–12, for 6 months	FIQ (depression)
Komatireddy *et al*., [17]	United States	49 men and women with RA assigned to a circuit training (n = 25, $\overline{x}$ ± SD = 57.7 ± 9.8 yrs of age, range 40–72 yrs) or control group (n = 24, $\overline{x}$ ± SD = 60.5 ± 11 yrs of age, range 35–76 yrs)	Circuit weight bearing with light loads and high repetitions, 7 exercises, 2–3 circuits/session, 12–15 reps,30-second rest between sets, 20–27 min/session, at least 3 x/wk, RPE of 3–4, for 12 wks	AIMS (depression)
Mannerkorpi et al., [18]	Sweden	69 women with FM assigned to either a training (n = 37, $\overline{x}$ ± SD = 45 ± 8 yrs of age) or control group (n = 32, $\overline{x}$ ± SD = 47 ± 11.6 yrs of age)	1 d/wk, 35 min/session, endurance, flexibility, coordination and relaxation exercises in a temperate pool for 6 months	FIQ (depression), AIMS (depression)
Minor *et al*., [19]	United States	115 men and women ages 21–83 yrs with RA or OA assigned to a pool (n = 47), walking (n = 36) or control group (n = 32)	3 x/wk, 1 hr/session (30 min of this was aerobic), 60-80% MHR, for 12 wks either in a pool (aerobic aquatics) or walk (aerobic walking) group	AIMS (depression)
Neuberger *et al*., [20]	United States	310 men and women age 55.5, range 40–70 yrs, with RA assigned to a class (n = 102), or home-based (videotape) exercise (n = 103), or control group (n = 105)	3 d/wk, 1 hr/session, low-impact aerobics (25.4 min, range 10–30 min/session) and strength training (16.67 min, range 15–20 min/session), 60-80% MHR, for 12 wks	CES-D, POMS
O’Reilly *et al*., [21]	United Kingdom	191 men and women with knee OA ages 40–80 yrs assigned to an exercise (n = 113, $\overline{x}$ ± SD = 61.9 ± 10.0 yrs of age) or control group (n = 78, $\overline{x}$ ± SD = 62.2 ± 9.7 yrs of age)	5 strengthening exercises, 20 reps per leg, performed at home on a daily basis for 6 months	HAD
Patrick *et al*., [22]	United States	249 men and women 55–75 yrs of age with OA assigned to either an exercise (n = 125, 65.7 yrs of age) or wait-list control group (n = 124, 66.1 yrs of age)	2-7 d/wk, 45–60 min/session of aquatic exercise, for 20 wks	CES-D
Penninx *et al*., [23]	United States	439 men and women ($\overline{x}$ ± SD = 68.8 ± 5.6 yrs of age) assigned to an aerobic (n = 149), resistance, (n = 146) or control group (n = 144)	78 wks of training; Aerobic group: 3 d/wk, 40 min/session, 50-70%HRR, walking; Resistance group: 3 d/wk, 40 min/session, 2 sets, 10 reps using dumbbells and cuff weights	CES-D
Rooks *et al*., [24]	United States	152 women with FM assigned to an aerobic and flexibility (n = 51, $\overline{x}$ ± SD = 48 ± 11 yrs of age), strength training, aerobic, and flexibility (n = 51, $\overline{x}$ ± SD = 50 ± 11 yrs of age) or control group (n = 50, $\overline{x}$ ± SD = 51 ± 12 yrs of age)	3 d/wk, of either aerobic (45 min walking) and flexibility exercises or aerobic (20 min walking), strength training (25 min, 6 exercises, 50% 1RM, 1–2 sets of 6–12 reps), and flexibility exercises for 16 wks	FIQ (depression), BDI
Sanudo *et al*., [25]	Spain	42 women with FM assigned to an exercise (n = 21, $\overline{x}$ ± SD = 55.5 ± 7.1 yrs of age) or control group (n = 21, $\overline{x}$ ± SD = 56.2 ± 8.5 yrs of age)	2 x/wk, aerobic (walk/jog, 10–15 min, 65-70% MHR), strength training (15–20 min, circuit of 8 exercises, 1 set, 8–10 reps), and flexibility exercises (10 min, 8–9 stations, 1 set, 3 reps for 30 sec), for 24 wks	BDI
Schachter *et al*., [26]	Canada	143 women with FM (20–55 yrs of age) assigned to a short bout (n = 56, $\overline{x}$ ± SD = 41.9 ± 8.6 yrs of age), long bout (n = 51, $\overline{x}$ ± SD = 41.3 ± 8.7 yrs of age), or control group (n = 36, $\overline{x}$ ± SD = 42.5 ± 6.7 yrs of age)	Home-based, videotape-based, low impact aerobic exercise to music. Short bout group: 2 x/d, 7.1 x/wk, 12.3 min/session, 60% HRR; Long-bout group: 1 x/d, 3.6 x/wk, 25.5 min/session, 60% HRR, for 16 wks	FIQ (depression)
Sencan *et al*. [27]	Turkey	40 women with FM assigned to an aerobic exercise (n = 20, $\overline{x}$ ± SD = 35.4 ± 9.6 yrs of age) or control TENS treatment group (n = 20, $\overline{x}$ ± SD = 35.55 ± 7.9 yrs of age)	3 x/wk, 40 min/session, aerobic exercise (cycle ergometer for 30 min, plus 5 min warm-up and 5 min cool-down) for 6 wks	BDI
Tomas-Carus *et al*. [28]	Portugal	34 women with FM assigned to aquatic exercise (n = 17, $\overline{x}$ ± SD = 51 ± 10 yrs of age) or control group (n = 17, $\overline{x}$ ± SD = 51 ± 9 yrs of age)	3 x/wk, pool exercises performed in warm water, 20 min aerobic, 60-65% MHR, 20 min strength exercises, 4 sets, 10 reps, for 12 wks	FIQ (depression)
Tomas-Carus *et al*. [29]	Portugal	33 women with FM assigned to aquatic exercise (n = 17, $\overline{x}$ ± SD = 50.7 ± 10.6 yrs of age) or control group (n = 16, $\overline{x}$ ± SD = 50.9 ± 6.7 yrs of age)	3 x/wk, pool exercises performed in warm water, 20 min aerobic, 60-65% MHR, 20 min strength exercises, 4 sets, 10 reps, for 8 months	FIQ (depression)
Valim *et al*. [30]	Brazil	76 women with FM assigned to an exercise (n = 38, $\overline{x}$ ± SD = 47 ± 10 yrs of age) or stretching group (n = 38, $\overline{x}$ ± SD = 44 ± 11 yrs of age)	3 x/wk, 45 min/session, aerobic exercise group walked, for 20 wks. Stretching (control) group 3 x/wk, 45 min/session, 17 exercises for flexibility but without raising HR, for 20 wks	BDI
Valkeinen *et al*. [31]	Finland	26 women with FM assigned to a strength training (n = 13, $\overline{x}$ ± SD = 60.2 ± 2.5 yrs of age, range 57–64 yrs) or control group (n = 13, $\overline{x}$ ± SD = 59.1 ± 3.5 yrs of age, range 54–65 yrs)	2 x/wk, 60–90 min/session, strength training, 7–8 exercises, 3–5 sets, 5–20 reps, 40-70% 1 RM, for 21 wks	BDI
Wang *et al*. [32]	United States	40 men and women with knee OA assigned to a Tai Chi (n = 20, $\overline{x}$ ± SD = 63 ± 8.1 yrs of age) or attention control group (n = 20, $\overline{x}$ ± SD = 68 ± 7 yrs of age)	2 d of supervised Tai Chi for 30 min; Tai Chi every day at home for at least 20 min; 10 forms of classic Yang style Tai Chi, for 12 wks	CES-D
Wang *et al*. [33]	United States	66 men and women with FM assigned to a Tai Chi (n = 33, $\overline{x}$ ± SD = 49.7 ± 11.8 yrs of age) or attention control group (n = 33, $\overline{x}$ ± SD = 50.5 ± 10.5 yrs of age)	2 d of supervised Tai Chi (Yang Style-10 forms) for 60 min; Tai Chi every day at home for at least 20 min, for 12 wks	CES-D
Wigers *et al*. [34]	Norway	40 men and women with FM assigned to an aerobic exercise (n = 20, $\overline{x}$ ± SD = 43 ± 9 yrs of age, range 23–62 yrs) or treatment-as-usual group (n = 20, $\overline{x}$ ± SD = 46 ± 9 yrs of age, range 29–73 yrs)	3 x/wk, 18–20 min/session of aerobic exercise with 1) music session, 2) aerobic games (tag, ball games) at 60-70% MHR, for 14 wks	VAS

Description of groups limited to those that met the inclusion criteria for the current meta-analysis. Number of subjects based on initial versus final number of subjects included in the studies. AIMS, Arthritis Impact and Measurement Scale for depression; BDI, Beck Depression Inventory; CES-D, Center for Epidemiologic Studies Depression Scale; d, day(s); DASS, Depression, Anxiety and Stress Scale; FIQ, Fibromyalgia Impact Scale for depression; FM, fibromyalgia; h, hour(s); HAD, Hospital Anxiety and Depression Scale; HR, heart rate; HRR, heart rate reserve; MHI, Mental Health Inventory; MHR, maximum heart rate; min, minute(s); OA, osteoarthritis; POMS, Profile of Moods States; RA, rheumatoid arthritis; reps, repetitions; RM, repetition maximum; ROM, range of motion; RPE, rate of perceived exertion; sec, second(s); SLE, systemic lupus erythematosus; VAS, Visual Analog Scale; wk, week(s); x, times; $\overline{x}$ ± SD; mean ± standard deviation; yr, year(s).

### Participant characteristics

A description of the participant characteristics is shown in Tables 1 and 2. The majority of participants were women; fourteen studies were limited to women [6,9,13-15,18,24-31] while fifteen included both men and women [7,8,10-12,16,17,19-23,32-34]. No study was limited to men. With respect to race/ethnicity and as reported by the original study authors, the majority of participants were non-Hispanic Whites for the fifteen studies in which data were reported [6,8,10,15,16,19,20,22-26,31-33]. Twelve of the fifteen studies included multiple races/ethnicities [8,10,15,16,19,20,22-24,26,32,33]. The other three studies were limited to non-Hispanic Whites [31] or other Hispanics [6,25]. Groups represented in the studies included Whites, African-Americans, Hispanics, Native-Americans and Aboriginals. Two studies reported that some of the participants smoked cigarettes [8,26] while one reported that some participants consumed alcohol [8]. Another two studies reported that exercise participants increased their exercise outside the actual intervention [10,25]. With respect to cardiovascular disease risk factors, one study was limited to overweight and obese participants [25] while twelve others included some participants who were overweight or obese [6,10,11,14,16,23,24,29-33]. Two studies reported that none of the exercise or control group participants had type 1 or type 2 diabetes [12,27], while one reported that none of the exercise group participants had type 2 diabetes [32]. Two studies reported that none of the participants were hypertensive [12,16] while six included some who were hypertensive [6,9,19,23,32,33].

**Table 2:** **Initial physical characteristics of participants**

	**Exercise**					**Control**		
**Variable**	**Groups (Number)**	$\overline{\boldsymbol{x}}\pm SD$	**Mdn**	**Range**	**Groups (Number)**	$\overline{\boldsymbol{x}}\pm SD$	**Mdn**	**Range**
Age (yrs)	33	52.4 ± 9.7	51	35 - 71	28	52.2 ± 9.6	51	36 – 70
Height (cm)	7	160.3 ± 2.9	161	157 - 165	6	160.9 ± 2.0	161	158 – 164
Body weight (kg)	9	70.1 ± 4.9	69	63 - 77	8	71.4 ± 4.0	72	64 – 76
BMI (kg^.^m^2^)	15	28.8 ± 2.5	29	24 - 34	13	28.6 ± 2.9	29	25 – 32
Body fat (%)	3	34.9 ± 0.8	35	34 – 35	3	35.1 ± 2.9	36	32 – 38
Symptom duration (yrs)	14	12.2 ± 5.2	11	5 – 24	13	11.7 ± 4.2	10	5 – 19
Diagnosis (yrs)	10	5.5 ± 2.5	5	3 - 10	8	6.2 ± 2.8	6	3 - 11

Groups, number of groups in which data were available. BMI, body mass index; Mdn, median; $\overline{x}\pm SD$, mean ± standard deviation; yrs, years.

For type of AORC, 20 of 29 studies (69.0%) were limited to participants with fibromyalgia [6,7,9,10,12-16,18,24-31,33,34], five with osteoarthritis [11,21-23,32], and two with rheumatoid arthritis [17,20]. One study included participants with either rheumatoid arthritis or systemic lupus erythematosus [8] while another included participants with osteoarthritis or rheumatoid arthritis [19]. For the 14 exercise (40%) and 13 control (44.8%) groups in which sufficient data were provided, the number of years in which rheumatic symptoms were present ranged from 4.7 to 24.0 years in the exercisers ($\overline{x}$ ± SD = 12.2 ± 5.2, Mdn = 10.7) and 5.1 to 19.4 years in the controls ($\overline{x}$ ± SD = 11.7 ± 4.2, Mdn = 10.0). With respect to years since diagnosis, exercise groups ranged from 2.8 to 9.8 years ($\overline{x}$ ± SD = 5.5 ± 2.5, Mdn = 5.5) for the ten (28.6%) groups in which data were available while the control groups ranged from 2.5 to 10.5 years ($\overline{x}$ ± SD = 6.2 ± 2.8, Mdn = 6.3) for the eight (27.6%) groups in which data were provided.

### Exercise intervention characteristics

A description of the exercise interventions for each included study is shown in Table 1. As can be seen, the exercise interventions varied considerably. Length of training ranged from 4 to 78 weeks ($\overline{x}$ ± SD = 19 ± 16, Mdn = 16), frequency (34 groups reporting) from 1 to 9 times per week ($\overline{x}$ ± SD = 4 ± 2, Mdn = 3) and duration (31 groups reporting) from 12 to 83 minutes per session ($\overline{x}$ ± SD = 34 ± 17, Mdn = 30). For the eighteen groups (51.4%) in which data were provided, intensity of training was classified as low for three groups, moderate for eleven and high for four. Fifteen of the groups focused on aerobic exercise, five on strength training and eleven on both. Another four groups participated in meditative movement therapies that included either tai chi (three groups) or qigong (one group). Meditative movement therapies were considered as including both aerobic and strength training components. With respect to supervision, eighteen groups participated in supervised exercise, seven in unsupervised exercise and ten in both. The setting in which the groups exercised mirrored supervision, with eighteen taking place in a facility-based environment, seven in a home-based environment, and ten in both. For the 20 groups (57.1%) in which data were available, compliance, defined as the percentage of exercise sessions attended, ranged from 38% to 97% ($\overline{x}$ ± SD = 74 ± 13, Mdn = 75). Total minutes per week of exercise for the 30 groups (85.7%) in which data could be calculated, ranged from 30 to 360 ($\overline{x}$ ± SD = 108 ± 67, Mdn = 90). When adjusted for compliance (20 groups or 57.1% of all groups) total minutes per week ranged from 25 to 277 ($\overline{x}$ ± SD = 72 ± 60, Mdn = 57). Total minutes of training over the entire length of the interventions (30 groups) ranged from 360 to 9,360 ($\overline{x}$ ± SD = 2,080 ± 2,186, Mdn = 1,404). When adjusted for compliance (20 groups) total minutes ranged from 331 to 5,887 ($\overline{x}$ ± SD = 1,532 ± 1,609, Mdn = 839).

### Risk of bias assessment

**Figure 2:**
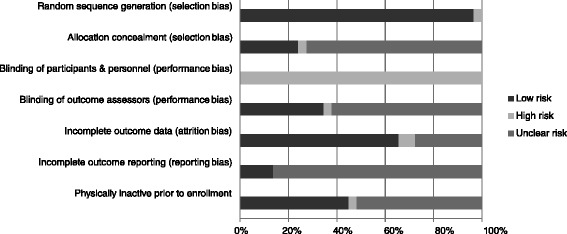
**Risk of bias.** Pooled risk of bias results using the Cochrane Risk of Bias Assessment Instrument.

Risk of bias results are shown in Figure 2 while results for each item from each study are shown in Additional file 3. Twenty-eight of twenty-nine studies [6-25,27-34] were considered to be at low risk with respect to sequence generation while one was considered to be at high risk [26]. Allocation concealment was poorly reported; seven studies were considered to be at low risk [15,21,24,25,29,32,33], one at high risk [26] and the remaining twenty-one at unclear risk [6-14,16-20,22,23,27,28,30,31,34]. Since it is impossible to blind participants in exercise intervention studies, all included trials were considered to be at a high risk of bias for the blinding of participants and personnel category [6-34]. Blinding of outcome assessment was also poorly reported, with ten studies considered being at low risk [6,11,12,15,17,20,24,25,29,30], one at high risk [21], and eighteen at unclear risk [7-10,13,14,16,18,19,22,23,26-28,31-34]. For incomplete data, that is, attrition bias, nineteen studies were considered to be at low risk for bias [6,7,10-13,15-18,22,23,25,26,29,31-34], two at high risk [20,30] and eight at unclear risk [8,9,14,19,21,24,27,28]. For selective outcome reporting, twenty-five studies were considered to be at an unclear risk [6-10,12-23,25-31,34] while four were considered low risk [11,24,32,33]. With regard to participants not exercising regularly prior to enrollment, thirteen studies were considered to be at low risk [7,9-11,16,19,20,22,23,26,28-30], one at high risk [8], and the remaining fifteen at unclear risk [6,12-15,17,18,21,24,25,27,31-34].

### Primary outcome

#### Depressive symptoms

**Figure 3:**
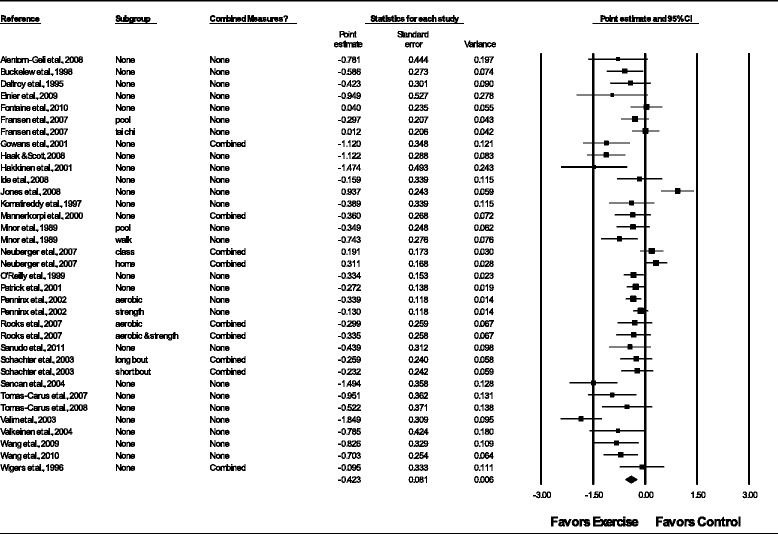
**Forest plot for changes in depressive symptoms.** Forest plot for point estimate changes in depressive symptoms. The black squares represent the mean difference while the left and right extremes of the squares represent the corresponding 95% confidence intervals. The middle of the black diamond represents the overall mean difference while the left and right extremes of the diamond represent the corresponding 95% confidence intervals. Combined measures represent those studies in which multiple assessment instruments for depression and/or per-protocol and intention-to-treat analyses were merged.

**Figure 4:**
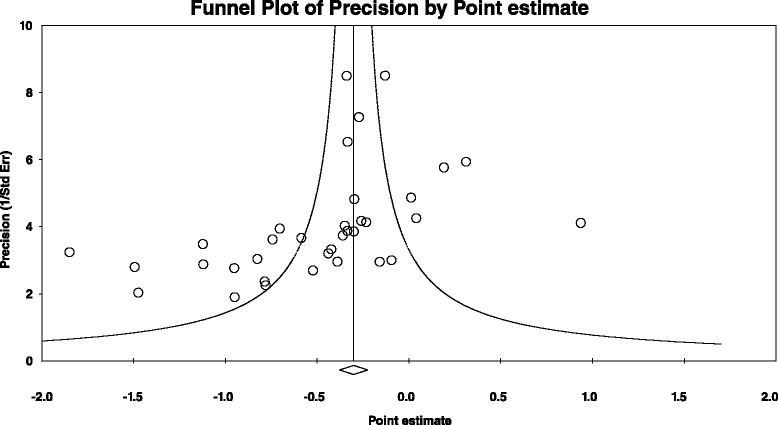
**Funnel plot for changes in depressive symptoms.** Small-study effects are apparently present given the lack of results in the lower right section of the funnel plot.

**Figure 5:**
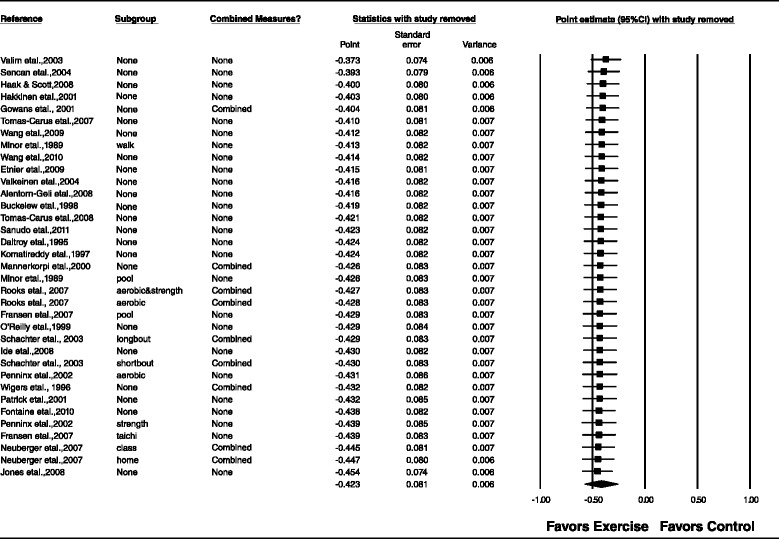
**Influence analysis for changes in depressive symptoms.** Influence analysis for point estimate changes in depressive symptoms with each corresponding study deleted from the model once. The black squares represent the mean difference while the left and right extremes of the squares represent the corresponding 95% confidence intervals. The middle of the black diamond represents the overall mean difference while the left and right extremes of the diamond represent the corresponding 95% confidence intervals. Results are ordered from smallest to largest reductions*.* Combined measures represent those studies in which multiple assessment instruments for depression and/or per-protocol and intention-to-treat analyses were merged.

**Figure 6:**
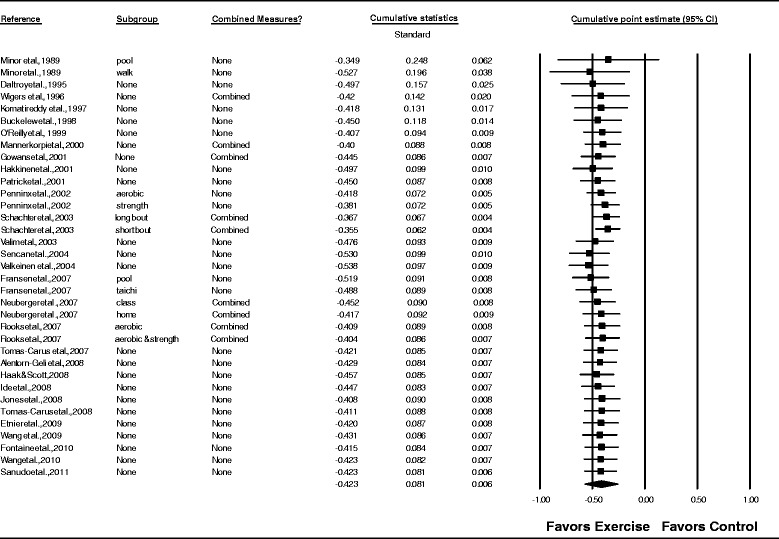
**Cumulative meta-analysis for changes in depressive symptoms.** Cumulative meta-analysis, ordered by year, for point estimate changes in depressive symptoms. The black squares represent the mean difference while the left and right extremes of the squares represent the corresponding 95% confidence intervals. The results of each corresponding study are pooled with all studies preceding it. The middle of the black diamond represents the overall mean difference while the left and right extremes of the diamond represent the corresponding 95% confidence intervals. Combined measures represent those studies in which multiple assessment instruments for depression and/or per-protocol and intention-to-treat analyses were merged.

Overall, there was a statistically significant reduction in depressive symptoms as well as a statistically significant and moderate amount of heterogeneity (Table 3 and Figure 3). In addition, 95% PIs were non-significant. Statistically significant small-study effects were observed as indicated by funnel plot asymmetry (Figure 4) as well as overlapping 95% CI based on Egger’s regression intercept test (β_0_, −2.4, 95% CI, −3.7 to −1.1) [62]. Reductions in depressive symptoms remained statistically significant when data were collapsed so that only one g represented each study ($\overline{x}$, −0.48, 95% CI, −0.67 to −0.30; Q = 164.4, *P* <0.001; *I*^*2*^ = 83.0%). When four outliers were deleted from the model [16,20,27,31], results remained statistically significant ($\overline{x}$, −0.44, 95% CI, −0.58 to −0.31; Q = 73.8, *P* <0.001; *I*^*2*^ = 59.4%). With each group deleted from the model once, results remained statistically significant across all deletions (Figure 5). The difference between the largest and smallest values with each group deleted was 0.05 (11.8%). Cumulative meta-analysis, ranked by year, demonstrated that results have been statistically significant since the first included study was published in 1989 (Figure 6) [19]. The NNT was 7 (95% CI, 6 to 11) with an estimated 3.1 million (95% CI, 2.0 to 3.7) US adults not currently meeting physical activity guidelines improving their depressive symptoms if they began and maintained a regular exercise program. Using Cohen’s U_3_ Index, the percentile reduction was 16.4% (95% CI, 10.4% to 21.9%).

**Table 3:** **Changes in primary and secondary outcomes**

**Variable**	**Studies (Number)**	**ES (Number)**	**Participants (Number)**	$\overline{\mathbf{x}}$ **(95% CI)**	**Q(** ***P*** **)**	***I*** ^***2***^ ***(%)***	**95% PI**
Primary							
- Depressive symptoms (*g)*	29	35	2449	−0.42 (−0.58, −0.26)*	126.9 (<0.001)**	73.2	−1.23, 0.38
Secondary							
- Body weight (kg)	3	3	226	−0.75 (−3.25, 1.74)	3.1 (0.21)	35.2	--
- BMI (kg^.^m^2^)	5	5	266	0.08 (−0.19, 0.36)	3.0 (0.55)	0	--
- Body fat (%)	3	3	121	−0.05 (−0.72, 0.62)	0.9 (0.65)	0	--
- Physical function (*g*)	21	26	1513	0.58 (0.46, 0.70)*	33.0 (0.13)	24.3	0.26, 0.90*
- Pain (*g*)	25	30	1971	−0.57 (−0.76, −0.38)*	119.6 (<0.001)**	75.7	−1.51, 0.36
- Quality-of-life (*g*)	18	21	1276	0.73 (0.53, 0.92)*	54.0 (<0.001)**	62.9	−0.03, 1.48
- Anxiety (*g)*	13	16	976	−0.63 (−0.86, −0.40)*	47.8 (<0.001)**	68.6	−1.49, 0.23
- VO_2max_ (ml^.^kg^.-1^ min^−1^)	7	10	590	1.73 (0.87, 2.59)*	40. 9 (<0.001)**	78.0	−1.21, 4.67
- Upper body strength (*g)*	6	9	530	0.51 (0.31, 0.71)*	**11.8 (0.16)**	32.3	0.04, 0.98*
- Lower body strength (g)	9	10	584	0.83 (0.49, 1.12)*	32.1 (<0.001)**	72.0	−0.28, 1.95
- Balance (*g)*	3	3	147	0.49 (−0.21, 1.19)	**8.1 (0.02)**	75.5	--

**Boldfaced** items indicate statistical significance. *statistically significant, non-overlapping 95% confidence intervals; **statistically significant (*P* ≤0.10). BMI, body mass index; ES, effect size; *g*, Hedges standardized mean difference, adjusted for small-sample bias; *I* ^*2*^ *(%)*, I-squared; Q(*P*), Cochran’s Q statistic and alpha value for Q; VO_2max_, maximum oxygen consumption; $\overline{x}$ (95% CI), mean and 95% confidence interval; 95% PI, 95% prediction intervals; --, insufficient data to calculate.

*Exploratory* moderator (categorical) analyses for changes in depressive symptoms are shown in Additional file 4. As can be seen, statistically significant within-group reductions in depressive symptoms were observed for the majority of analyses. However, between-group differences were limited to gender, with women only groups experiencing greater reductions in depressive symptoms than mixed groups (no study was limited to men) and type of AORC (reductions in depressive symptoms greater in fibromyalgia versus rheumatoid arthritis participants). However, as can be seen, the results for those groups comprised of those with rheumatoid arthritis, osteoarthritis or rheumatoid arthritis and rheumatoid arthritis or systemic lupus erythematosus, were limited to three, two and one results, respectively.

*Exploratory* meta-regression analyses for changes in depressive symptoms and selected continuous covariates are shown in Additional file 5. As can be seen greater reductions in depressive symptoms were associated with increases in BMI (R^2^ = 0.90), greater reductions in pain (R^2^ = 0.21), improvements in quality-of-life (R^2^ = 0.46) and decreases in static balance (R^2^ = 0.81). However, the finding for static balance was limited to three effect sizes.

### Secondary outcomes

Changes in secondary outcomes are shown in Table 3. As can be seen, there were no statistically significant changes in body weight, BMI in kg^.^m^2^, percent body fat or static balance. In contrast, a statistically significant improvement was observed for physical function. Heterogeneity was considered to be very low and non-significant. In addition, PI were statistically significant. Changes were equivalent to a percentile improvement of 21.9%. However, statistically significant small-study effects were observed (95% CI, 0.16 to 2.72). With each study deleted from the model once, improvements remained statistically significant, ranging from 0.55 to 0.60. Results were similar when data were collapsed so that only one result represented each study ($\overline{x}$, 0.57, 95% CI, 0.45 to 0.70; Q = 27.8, *P* = 0.11; *I*^*2*^ = 28.1%). Cumulative meta-analysis showed that improvements in physical function have been statistically significant since the year 1989, the year that the first included study was conducted [19].

Statistically significant decreases in pain were found with percentile reductions equivalent to 21.5%. However, heterogeneity was both statistically significant and large. In addition, PI were non-significant and small-study effects were observed (95% CI, −4.76 to −1.17). With each study deleted from the model once, reductions remained statistically significant, ranging from −0.52 to −0.60. With three outliers deleted from the model [14,26,27], results remained statistically significant ($\overline{x}$, −0.52, 95% CI, −0.67 to −0.36; Q = 66.6, *P* <0.001; *I*^*2*^ = 61.0%). Results were similar when data were collapsed so that only one result represented each study ($\overline{x}$, −0.63, 95% CI, −0.83 to −0.44; Q = 98.2, *P* <0.001; *I*^*2*^ = 75.6%). Cumulative meta-analysis showed that results have been statistically significant since the year 1999.

Statistically significant increases in quality-of-life were also observed with percentile improvements of 26.6%. Heterogeneity was considered to be statistically significant but moderate. Prediction intervals were non-significant while statistically significant small-study effects were observed (95% CI, 1.67 to 4.90). With each study deleted from the model once, increases remained statistically significant, ranging from 0.67 to 0.76. With two outliers deleted from the model [14,28], results remained statistically significant ($\overline{x}$, 0.61, 95% CI, 0.47 to 0.76; Q = 27.0, *P* = 0.08; *I*^*2*^ = 33.3%). Heterogeneity was reduced by 29.6%, from moderate to low. Cumulative meta-analysis showed that improvements in quality-of-life have been statistically significant since the year 2000.

For anxiety, statistically significant decreases were observed along with statistically significant and moderate heterogeneity. Prediction intervals were not statistically significant and significant small-study effects were observed (95% CI, −6.98 to −2.72). Decreases in anxiety were equivalent to a percentile reduction of 23.5%. With each study deleted from the model once, deceases remained statistically significant, ranging from −0.56 to −0.67. Results were similar when data were collapsed so that only one result represented each study ($\overline{x}$, −0.64, 95% CI, −0.90 to −0.38; Q = 46.9, *P* <0.001; *I*^*2*^ = 74.4%). Cumulative meta-analysis showed that decreases in anxiety have been statistically significant since 1989, the year that the first included study was conducted [19].

Increases in aerobic fitness, as assessed by VO_2max_ in ml^.^kg^-1.^min^−1^, were statistically significant and equivalent to a percentile improvement of 24.5%. Heterogeneity was statistically significant and large. In addition, PI were non-significant. Small-study effects were not statistically significant (95% CI, −5.49 to 4.33). With each study deleted from the model once, increases remained statistically significant, ranging from 1.51 to 2.01 ml^.^kg^-1.^min^−1^. Results were similar when data were collapsed so that only one finding represented each study ($\overline{x}$, 1.62 ml^.^kg^-1.^min^−1^, 95% CI, 0.57 to 2.67; Q = 37.9, *P* <0.001; *I*^*2*^ = 84.2%). Cumulative meta-analysis showed that increases in VO_2max_ in ml^.^kg^-1.^min^−1^ have been statistically significant since the year 2003.

Changes in both upper and lower body strength were statistically significant. For upper body strength, increases were equivalent to a percentile increase of 19.5%. Heterogeneity was non-significant and low. In addition, no statistically significant small-study effects were observed (95% CI, −1.56 to 4.11) and PI were statistically significant. With each study deleted from the model once, increases remained statistically significant, ranging from 0.44 to 0.58. Results were similar when data were collapsed so that only one result represented each study ($\overline{x}$, 0.50, 95% CI, 0.33 to 0.67; Q = 5.5, *P* = 0.36; *I*^*2*^ = 9.0%). Cumulative meta-analysis showed that increases in upper body strength have been statistically significant since the year 1989, the year that the first included study was conducted [19]. For lower body strength, increases were statistically significant and equivalent to a percentile improvement of 29.7%. Heterogeneity was statistically significant but moderate. Statistically significant small-study effects were observed (95% CI, 1.36 to 5.82) while PI were non-significant. With each study deleted from the model once, increases remained statistically significant, ranging from a *g* of 0.70 to 0.91. Results were similar when data were collapsed so that only one result represented each study ($\overline{x}$, 0.81, 95% CI, 0.46 to 1.16; Q = 28.9, *P* <0.001; *I*^*2*^ = 72.3%). Cumulative meta-analysis showed that increases in lower body strength have been statistically significant since the year 1999.

## Discussion

### Findings

The primary purpose of this study was to use the aggregate data meta-analytic approach to determine the effects of exercise (aerobic, strength training or both) on depressive symptoms in adults with AORC: fibromyalgia, osteoarthritis, rheumatoid arthritis and systemic lupus erythematosus. The overall findings suggest that exercise is associated with important reductions in depressive symptoms among selected adults with AORC. This interpretation is supported by: (1) non-overlapping 95% CI for overall results, (2) consistency with overall results when each study was deleted from the model once (influence analysis), (3) consistency with overall results when outliers were deleted from the model (outlier analysis), (4) consistency with overall results when data were collapsed so that one result represented each study (independence analysis), (5) significance of results over the entire time period that included studies were conducted (cumulative meta-analysis), (6) low NNT and (7) number of people who could potentially benefit by initiating and maintaining a regular exercise program. Alternatively, confidence in the overall findings for depressive symptoms may be weakened by one or more of the following five factors. First, while a random-effects model that incorporates heterogeneity into the analysis was used, a moderate amount of heterogeneity, based on a fixed-effect model, was observed. This suggests that selected but unknown factors may be associated with the magnitude of change, if any, in depressive symptoms among adults with AORC. Importantly, heterogeneity in meta-analysis is not only common [71], but also relevant, as there is no need to combine studies exactly alike since their findings, within statistical error, would be the same [72]. Second, statistically significant small-study effects were observed. While publication bias is one possible explanation for this finding, other potential factors may be at play here. These include: (1) other reporting biases (selective outcome and/or analysis reporting), (2) poor methodological quality leading to inflated effects in smaller studies, (3) true heterogeneity, (4) sampling variation leading to an association between the intervention effect and standard error and (5) chance [42]. Third, overlapping PI were observed. However, PI should not be confused with CI since PI are based on a random mean effect while CI are not [60]. Nevertheless, non-overlapping PI would give one more confidence in any overall findings observed. Fourth, many of the studies were at a high or unclear risk of bias for several items from the Cochrane Risk of Bias Assessment Instrument. These include: (1) allocation concealment, (2) blinding of outcome assessors, (3) attrition, including reasons, according to each group and (4) physical activity levels of the participants prior to study enrollment. While all of the included studies were also considered to be at a high risk of bias for the blinding of participants and personnel category, it is probably impossible to blind participants to group assignment in exercise intervention studies. Therefore, the best that might be expected is to blind personnel to group assignment. Fifth, some of the differences observed for the *exploratory* moderator and meta-regression results suggest that selected factors may affect any overall conclusions drawn. These include, but are not necessarily limited to: (1) method used to assess depressive symptoms, (2) type of AORC, (3) exercise delivery and (4) observed associations between reductions in depressive symptoms and BMI, pain, quality of life and static balance. However, the moderator and meta-regression results need to be viewed cautiously for at least three reasons. First, because of missing data for different variables from different studies, a common occurrence in meta-analysis, multiple meta-regression analysis was not feasible. Thus, the inability to control for relevant variables may have resulted in spurious findings for the separate analyses conducted. Second, the small sample sizes for many of the categorical analyses as well as some of the meta-regression analyses, for example, static balance, may have yielded spurious results. Third, studies are not randomly assigned to covariates in meta-analysis. Thus, they are considered to be observational in nature. Consequently, the results of moderator and meta-regression analyses conducted in any meta-analysis does not support causal inferences and should be viewed as nothing more than exploratory [66]. Large, well-designed randomized controlled trials would be needed to address this issue adequately. Given this, future randomized controlled trials may want to address some of the differences and associations observed in the current meta-analysis.

The direction of effect for reductions in depressive symptoms found in the current meta-analysis (SMD, −0.42, 95% CI, −0.58 to −0.26) are consistent with previous meta-analytic work by Busch *et al*. (SMD, −0.61, 95% CI, −0.99 to −0.23) [37], Hauser *et al*. (SMD, −0.32, 95% CI, −0.53 to −0.12) [38] and Herring *et al*. (SMD, 0.29, 95% CI, −0.16 to −0.43) [39] in participants with fibromyalgia. While the overall magnitude of effect varies between all four meta-analyses, the 95% CI for all studies overlap, suggesting no statistically significant difference between them. The former notwithstanding, one possible reason for the differences in the overall magnitude of effect may have to do with the fact that the exact same studies were not included in any of these meta-analyses.

The overall magnitude of reduction for depressive symptoms observed in the current study (*g = −*0.42) is approximately 38.1% greater than that reported in a previous meta-analysis of pharmacologic interventions limited to participants with fibromyalgia [73]. Hauser *et al*. examined the effects of tricyclic and tetracyclic antidepressants, selective serotonin reuptake inhibitors, serotonin and noradrenaline reuptake inhibitors and monoamine oxidase inhibitors on depressed mood in 18 studies representing 1,427 participants [73]. Overall, a statistically significant decrease in depressed mood was observed, (standardized mean difference reduction, −0.26; (95% CI, −0.39 to −0.12) [73]. However, as opposed to the current meta-analysis, no statistical heterogeneity was observed (*I*^*2*^ = 0%) [73]. In addition, the confidence intervals between the current meta-analysis and the Hauser *et al*. [73] meta-analysis are overlapping.

In addition to statistically significant reductions in depressive symptoms, improvements were also observed for physical function, pain, quality-of-life, anxiety, VO_2max_ in ml^.^kg^-1.^min^−1^, and upper and lower body strength. As opposed to pharmacologic interventions that generally target one outcome, these findings provide evidence to support the use of exercise for improving multiple outcomes. This notwithstanding, the findings for these secondary outcomes need to be interpreted with caution given that they were only included if depressive symptoms was included as an outcome. Consequently, the data from which these results were derived may represent a biased sample.

### Implications for research

The results of the current systematic review with meta-analysis have at least seven implications for the reporting and conduct of future randomized controlled trials. First, based on the Cochrane Risk of Bias Assessment Instrument [49], it is recommended that future randomized controlled trials on the effects of exercise in adults with AORC improve their reporting with respect to several potential sources of bias. These include; (1) allocation concealment, (2) blinding of outcome assessors, (3) attrition, including reasons, according to each group and (4) the physical activity levels of the participants prior to study enrollment. While all of the included studies were also considered to be at a high risk of bias for the blinding of participants and personnel category, it is probably impossible to blind participants to group assignment in exercise intervention studies. Therefore, the best that might be expected is to blind personnel to group assignment.

Second, only four studies used both the per-protocol and intention-to-treat approach in the analysis of their data [12,24,26,34]. Given this, it is suggested that future studies include both in order to gain a better understanding regarding the efficacy and effectiveness of exercise for improving depressive symptoms in adults with AORC [74].

Third, very little data were available for adverse events as well as the cost-effectiveness of the interventions employed. Since these are important factors to consider when making decisions regarding the choice of one intervention over another, it is suggested that future studies collect and report this information.

Fourth, complete information should be collected and reported on the exercise intervention(s) employed. For example, in the current meta-analysis, data on the intensity of exercise as well as compliance with the exercise intervention were underreported. More specifically, future studies should report complete information on the length, frequency, intensity and duration of exercise, mode(s) used, and compliance with the exercise protocol. Also, information on the setting in which exercise takes place as well as supervision status should be reported. Because of the potential for physical activity compensation to occur [75], data should also be collected and reported on total physical activity for all groups included in the study. The rationale for this suggestion is based on the possibility that physical activity levels beyond any intervention(s) may increase or decrease in the intervention and/or control groups. For example, two of the included studies reported that exercise participants increased their physical activity beyond the exercise intervention [10,25]. Increases or decreases such as these may negatively impact the results of one or more outcomes and may be especially problematic when trying to address the issue of dose–response.

Fifth, since the dose–response effects of exercise on depressive symptoms in adults with AORC are not known, it is suggested that future randomized controlled trials address this issue. The determination of such is critical for the development of optimal exercise programs for improving depressive symptoms in adults with AORC. Along those lines and as previously mentioned, it would appear plausible to suggest that an examination of some of the differences and associations observed in the current meta-analysis would be appropriate. For example, if feasible, a multi-arm, randomized controlled exercise intervention trial that includes participants with different types of AORC may be appropriate.

Sixth, while fourteen of the included studies were limited to women [6,9,13-15,18,24-31] and fifteen were mixed [7,8,10-12,16,17,19-23,32-34], none were limited to men. Given that the age-adjusted prevalence of doctor-diagnosed arthritis in the US has been reported to be 18.6% in men and that the biology between men and women differs, future studies limited to men or that include both men and women with results partitioned according to sex, seem appropriate.

Seventh, since no study was limited to participants with systemic lupus erythematosus and only two were limited to adults with rheumatoid arthritis [17,20], future exercise intervention studies may want to focus on these populations. Such a focus may allow one to draw more definitive conclusions regarding the effects of exercise on depressive symptoms in these specific populations.

In addition to the reporting and conduct of future randomized controlled trials, the current meta-analysis provides at least three implications for future systematic reviews with meta-analysis. First, the *a priori* plan of the current study was to conduct a one-step IPD meta-analysis [51]. Nevertheless, because of: (1) the ability to obtain IPD from only 24.1% of eligible studies, (2) the inability to resolve discrepancies between the IPD provided and data reported in the published studies, for example, final sample sizes, and (3) the potential loss of power with fewer included studies, a *post hoc* decision was made to conduct an aggregate data meta-analysis, an approach similar to conducting a two-step meta-analysis with IPD [51]. While IPD meta-analysis is considered by some to be the gold standard [51,76], primarily because of the potential to conduct covariate analyses at the participant level, this has to be considered with respect to obtaining IPD from all eligible studies. In addition, causal inferences based on covariate analyses, whether conducted using IPD or aggregate data, cannot be made given that experiments are never randomly assigned to covariates [66,77]. Furthermore, the time and costs associated with conducting an IPD meta-analysis have been shown to be substantially greater than conducting an aggregate data meta-analysis. For example, in 1997, Steinberg *et al*. estimated the cost for 12 ovarian cancer studies to be $259,300 for conducting an IPD meta-analysis versus $48,665 for an aggregate data meta-analysis [78]. However, this 5.3 times greater cost has been suggested by others to be 8 times greater since the research team continued to work on the project after funding ended [77]. Importantly, the use of IPD is not always well established. To illustrate, when examining overall effects, the primary purpose of meta-analysis [72], studies claiming the superiority of IPD have been based on comparisons of a different number of studies between IPD and aggregate data [79,80]. However, when an indistinguishable or a nearly indistinguishable number of studies were included, the overall results were found to be analogous [78,81,82]. Finally, despite the increased use of IPD in recent years [83], the aggregate data approach is still the most frequently used when conducting a meta-analysis. Given this, future investigators planning a meta-analysis should think very carefully about the feasibility and potential gain derived from conducting an IPD versus aggregate data meta-analysis.

Second, given that the secondary outcomes included in the current meta-analysis may represent a biased sample, future meta-analytic work that includes one or more of these as a primary outcome might be important. For example, recent research has shown that the prevalence of anxiety among US adults was approximately twice as high as depression (30.5% versus 17.5%), with US population estimates of 11.5 million for anxiety and 6.6 million for depression [4]. However, a previous systematic review by the first two authors did not identify any previous systematic reviews with meta-analysis that met their inclusion criteria with respect to the effects of exercise on anxiety in adults with AORC (unpublished results). Given the reductions observed for anxiety in the current meta-analysis, it is suggested that a full systematic review with meta-analysis that includes anxiety as a primary outcome is warranted.

Third, the ultimate goal in the treatment of any condition is the identification of what treatments work best, that is, comparative effectiveness research. However, it is highly unlikely that any large multi-arm randomized controlled trial will ever be conducted that includes all possible pharmacologic and non-pharmacologic interventions that address the effects of depressive symptoms in adults with AORC. Alternatively, one cost-effective approach is the use of network meta-analysis, an increasingly popular method that allows one to incorporate both direct and indirect evidence in decisions about which treatment works best [84,85]. To the best of the investigative team’s knowledge, no such study exists with respect to depressive symptoms in adults with AORC.

### Implications for practice

The results of the current meta-analysis in adults with AORC have relevant implications for practice. Overall, it appears that exercise may improve depressive symptoms as well as a number of other outcomes (physical function, pain, quality-of-life, anxiety, VO_2max_ in ml^.^kg^-1.^min^−1^ and strength) in selected adults with AORC. Given this and despite the fact that no dose–response effects of exercise on depressive symptoms were identified and there was a lack of reporting for adverse events and cost-effectiveness, it would appear plausible to suggest that exercise might be a valuable addition to the treatment of adults with AORC. While such programs may need to be individually tailored to each person’s specific condition, following the general guidelines recommended by the US Centers for Disease Control and Prevention in adults with arthritis may be an appropriate starting point, especially if viewed from a community-based, public health perspective [86]. This includes 150 minutes per week of moderate-intensity aerobic activity, for example, brisk walking, 75 minutes per week of vigorous-intensity aerobic activity, for example, water aerobics, or some equivalent combination of both moderate and vigorous-intensity activity [86]. In addition, muscle strengthening exercises using equipment such as resistance bands is recommended on two or more days per week as well as balance exercises, for example, standing on one foot, at least three days per week [86]. When initiating an exercise program, it is suggested that participants with AORC: (1) start with activity performed over a short duration and at a low intensity, (2) modify one’s activity when arthritis symptoms increase, (3) participate in activities that do not place undue stress on the joints, for example, swimming versus running, (4) exercise in environments that are safe, and (5) seek guidance from a healthcare professional or certified exercise specialist [86].

### Strengths and limitations of current study

#### Strengths

There are least four *potential* strengths of the current meta-analysis. First, to the best of the investigative team’s knowledge, this is the first meta-analysis to examine the effects of exercise on depressive symptoms as a primary outcome in adults with AORC beyond those with fibromyalgia [36]. Thus, this adds important information as well as direction for future research and practical application regarding the effects of exercise on depressive symptoms in adults with AORC. Second, the inclusion of the NNT provides practical information to aid decision-makers in deciding what treatments to recommend or prioritize over others when attempting to reduce depressive symptoms in adults with AORC. Third, gross estimates of the number of US adults with AORC who might reduce their depressive symptoms by participating in a regular exercise program can help aid decision-makers and others in allocating the resources necessary for increasing exercise in this population. Fourth, the calculation and inclusion of PI can aid researchers when planning future randomized controlled trials on this topic.

#### Limitations

The results of the current meta-analysis should be viewed with respect to the following five *potential* limitations. First, a large number of statistical tests were conducted but no adjustments were made for multiple testing because of the concern about missing possibly important findings that could be pursued in future randomized controlled trials [87]. While this may be viewed by some as a ‘fishing expedition’ , the investigative team felt that these pre-planned analyses were important for providing investigators with potential direction for future randomized controlled trials. Nevertheless, some statistically significant results observed may have been chance findings. Second, the sample sizes for many of the analyses were small and, thus, probably underpowered to find a true effect or difference. In addition, the generalizability of results based on these small sample sizes is questionable. Third, given the different assessment instruments used as well as a lack of information provided on the severity of depressive symptoms upon study entry, the investigative team was unable to assess accurately whether greater decreases might be achieved by those with greater depressive symptoms at baseline. Fourth, the weaknesses and limitations of the studies included in any meta-analysis are inherited by the meta-analysis itself and, thus, may have a deleterious effect on any findings and conclusions drawn. Fifth, like any meta-analysis, the results of the current investigation may be prone to ecological fallacy and/or Simpson’s Paradox [77].

## Conclusions

Exercise is associated with decreases in depressive symptoms among selected adults with AORC. A need exists for additional, well-designed and reported randomized controlled trials on this topic.

## Additional files

Additional file 1:
**Search strategy for Scopus database.** This file provides the search strategy used for the Scopus database. Additional file 2:
**Excluded studies, including reasons.** This file provides a reference list of all excluded studies, including the reasons for exclusion. Additional file 3:
**Cochrane risk of bias results for each item from each included study.** This file provides an item analysis, by study, of the risk for bias using the Cochrane Risk of Bias Assessment Instrument. Additional file 4:
**Table of categorical analyses results for changes in depressive symptoms.** This file provides a table of all moderator analyses conducted. Additional file 5:
**Mixed effects meta-regression results for changes in depressive symptoms.** This file provides a table of all meta-regression analyses conducted.

## Abbreviations

AORC: arthritis and other rheumatic conditions
BMI: body mass index
CI: confidence intervals
CINAHL: Cumulative Index of Nursing and Allied Health Literature
NNR: number needed to read
NNT: number-needed-to-treat
PEDro: Physiotherapy evidence database
PI: prediction intervals
SMD: standardized mean difference
US: United States
VO_2max_: maximum oxygen consumption
