# Supplementary material for: Effects of exercise on depression in adults with arthritis: a systematic review with meta-analysis of randomized controlled trials
Source: Arthritis Res Ther. 2015 Feb 3;17(1):21. doi: 10.1186/s13075-015-0533-5 (PMC4467075; doi:10.1186/s13075-015-0533-5)
Supplement: Additional file 1: — Search strategy for Scopus database. This file provides the search strategy used for the Scopus database. [file 13075_2015_533_MOESM1_ESM.docx]

Additional File 1. Search Strategy for Scopus Database.

SCOPUS Database Search

(((((TITLE-ABS-KEY((exercising OR exercised OR exercise OR exertion OR aerobic* OR walk* OR jogging OR jogger OR runner OR runs OR bicycle* OR dancing OR dancer OR dances OR soccer OR rugby OR baseball* OR basketball* OR swim* OR hopscotch OR football)) AND PUBYEAR > 1980) OR (TITLE-ABS-KEY((activit* OR educat* OR fitness OR therapy OR therapies OR therapeutic*) W/3 physical) AND PUBYEAR > 1980) OR (TITLE-ABS-KEY("physical exertion" OR " physical therapy modalities") AND PUBYEAR > 1980) OR (TITLE-ABS-KEY((weight W/3 lift*)) AND PUBYEAR > 1980) OR (TITLE-ABS-KEY((strength OR resistance OR circuit OR enduran* OR aerob* OR physical OR fit OR fitness) W/6 train*) AND PUBYEAR > 1980) OR (TITLE-ABS-KEY(train* W/3 cross) AND PUBYEAR > 1980)) OR (TITLE-ABS-KEY("Exercise Therapy" OR water OR sports) AND PUBYEAR > 1980)) AND (((TITLE-ABS-KEY("rheumatorid arthritis" OR osteoarthritis OR fibromyalgia) AND PUBYEAR > 1980) AND ((TITLE-ABS-KEY(senior OR elder* OR older OR geriatric OR geriatrics OR aged OR adult OR adults) AND PUBYEAR > 1980) OR (TITLE-ABS-KEY("middle age" OR "middle aged" OR "young adult") AND PUBYEAR > 1980))) AND ((TITLE-ABS-KEY(depression OR depressed OR depressing OR depressive OR "mental depression" OR "depressive disorder") AND PUBYEAR > 1980) OR (TITLE-ABS-KEY("Dysthymic Disorder") AND PUBYEAR > 1980)))) AND (TITLE-ABS-KEY((human OR humans)) AND PUBYEAR > 1980)) AND (((KEY("Randomized Controlled Trials as Topic" OR "randomized controlled trial") AND PUBYEAR > 1980) OR (KEY("Random Allocation" OR "Double Blind Method") AND PUBYEAR > 1980) OR (KEY("single blind Method" OR "clinical trial*") AND PUBYEAR > 1980) OR (KEY("multicenter study") AND PUBYEAR > 1980) OR (KEY("Clinical Trials as topic") AND PUBYEAR > 1980)) OR ((TITLE-ABS-KEY((placebo* OR (clinical W/1 trial*))) AND PUBYEAR > 1980) OR (TITLE-ABS-KEY((allocat* W/2 random*)) AND PUBYEAR > 1980) OR (TITLE-ABS-KEY((randomly W/3 allocat*)) AND PUBYEAR > 1980) OR (TITLE-ABS-KEY((singl* OR doubl* OR treb* OR tripl*) W/1 trial*) AND PUBYEAR > 1980) OR (TITLE-ABS-KEY((singl* OR doubl* OR treb* OR tripl*) W/1 blind*) AND PUBYEAR > 1980) OR (TITLE-ABS-KEY((singl* OR doubl* OR treb* OR tripl*) W/1 mask*) AND PUBYEAR > 1980)))))
