# Supplementary material for: Effects of exercise on depression in adults with arthritis: a systematic review with meta-analysis of randomized controlled trials
Source: Arthritis Res Ther. 2015 Feb 3;17(1):21. doi: 10.1186/s13075-015-0533-5 (PMC4467075; doi:10.1186/s13075-015-0533-5)
Supplement: Additional file 2: — Excluded studies, including reasons. This file provides a reference list of all excluded studies, including the reasons for exclusion. [file 13075_2015_533_MOESM2_ESM.docx]

Additional file 2. Excluded studies, including reasons.

(1) ORAL PAPERS. Disability & Rehabilitation 2000 October 2;22:2-18. Not a randomized controlled trial (RCT)

(2) Free Communications. Journal of Sport & Exercise Psychology 2002 June 2;24:S25-S137. Abstract

(3) Annual Meeting Program Abstracts. Headache: The Journal of Head & Face Pain 2003 May;43(5):509-92. Abstract

(4) Posters Monday 2 August Drug Discovery & Development (PO-001 - PO-011). Clinical & Experimental Pharmacology & Physiology 2004 August 2;31:A51-A202. Abstract

(5) Abstracts. Psychology & Health 2004 June 2;19:s7-s192. Abstract

(6) Abstracts for the International Society for Aging and Physical Activity's 6th World Congress on Aging and Physical Activity: From Research to Action for an Aging Society London, Ontario, Canada, August 3-7, 2004. Journal of Aging & Physical Activity 2004 July;12(3):246-460. Abstract

(7) Ottawa Panel Evidence-Based Clinical Practice Guidelines for Therapeutic Exercises and Manual Therapy in the Management of Osteoarthritis. Physical Therapy 2005 September;85(9):907-71. Review article

(8) CAM Health Services and Policy Research in Canada - New Directions: Abstracts from the First Annual IN-CAM Symposium, December 4&5, 2004, Toronto, Canada. Journal of Complementary & Integrative Medicine 2005 January;2(1):1-57. Abstract

(9) AAPM Annual Meeting Abstracts. Pain Med (USA) 2005 April;6(2):165-97. Abstract

(10) Program Abstracts. Headache: The Journal of Head & Face Pain 2006 May;46(5):833-904. Abstract

(11) Abstracts/Poster Presentations. Nutrition & Dietetics 2006 March 2;63:A25-A56. Abstract

(12) ABSTRACTS. Applied Physiology, Nutrition & Metabolism 2007 September;32(S1):S1-S97. Abstract

(13) 2007 CSEP Annual Scientific Conference / 2007 Conference Scientifique Annuel de la SCPE. Applied Physiology, Nutrition & Metabolism 2007 September 2;32:S1-S98. Abstract

(14) Poster Presentations. Journal of Aging & Physical Activity 2008 July 2;16:S20-S61. Abstract

(15) Monday 21st July 2008. International Journal of Psychology 2008 June;43(3/4):1-167. Abstract

(16) AMERICAN ACADEMY OF PAIN MEDICINE ANNUAL MEETING ABSTRACTS 2009. Pain Med (USA) 2009 January;10(1):199-278. Abstract

(17) Abstracts of the Canadian Obesity Network's 1st National Obesity Summit. Applied Physiology, Nutrition & Metabolism 2009 April;34(2):235-305. Abstract

(18) SCIENTIFIC ABSTRACTS. JGIM: Journal of General Internal Medicine 25, 205-567. 6-2-2010. Abstract,

(19) Other complementary therapies. Focus Altern Complement Ther 2010;15(2):175-9. Review article

(20) Management of fibromyalgia. Drug Ther Bull 2010;48(8):89-93. Review article

(21) AAPM 2010 ANNUAL MEETINGS ABSTRACTS. Pain Med (USA) 2010 February;11(2):284-335. Abstract

(22) Exercise Comes of Age as Medicine for Older Adults. President's Council on Physical Fitness & Sports Research Digest 2010 June;11(2):1-13. Review article

(23) Free Papers. Knee Surgery, Sports Traumatology, Arthroscopy 2010 June 2;1-73. Abstract

(24) Wednesday 15th August 2012 Day Theme: Falls and Fractures/Balance and Bone Health: Plenary Keynotes. Journal of Aging & Physical Activity 2012 August 2;20:S102-S201. Abstract

(25) Tuesday 14th August 2012 Day Theme: Weil-Being, Quality of Life, and Cognitive Function: Plenary Keynotes. Journal of Aging & Physical Activity 2012 August 2;20:S7-S101. Abstract

(26) Cidem M, Rezvani A, Karacan I. Assessment of affective temperament in knee osteoarthritis patients and its effects on physical therapy response. Turk Klinikleri J Med Sci 2011;31(6):1372-6. Not a randomized controlled trial (RCT)

(27) Özkurt S, Dönmez A, Karagülle MZ, Uzunoglu E, Turan M, Erdogan N. Balneotherapy in fibromyalgia: A single blind randomized controlled clinical study. Rheumatol Int 2012;32(7):1949-54. Study less than 4 weeks

(28) Abdollahipour R. EGREPA 2010 Conference. European Reviews of Aging & Physical Activity 2010 September;7(2):71-104. Off topic

(29) Abou-Raya S, Abou-Raya A, Helmii M. Duloxetine for the management of pain in older adults with knee osteoarthritis: randomised placebo-controlled trial. Age and Ageing 2012;41(5):646-52. Drug intervention study

(30) Achterberg TJ, Wind H, De Boer AGEM, Frings-Dresen MHW. Factors that Promote or Hinder Young Disabled People in Work Participation: A Systematic Review. Journal of Occupational Rehabilitation 2009;19(2):129-41. Review article

(31) Adie JW, Duda JL, Ntoumanis N. FREE COMMUNICATIONS. Journal of Sport & Exercise Psychology 2006 June 2;S23-S198. Abstract

(32) Adler PA. The effects of Tai Chi on pain and function in older adults with osteoarthritis (dissertation) [Dissertation]. Cleveland, Ohio: Case Western Reserve University; 2007. Dissertation or Thesis

(33) Ahern M, Nicholls E, Simionato E, Clark M, Bond M. Clinical and psychological effects of hydrotherapy in rheumatic diseases. Clin Rehabil 1995 August 1;9(3):204-12. Subjects were active, NOT sedentary

(34) Ahsin S, Saleem S, Bhatti AM, Iles RK, Aslam M. Clinical and endocrinological changes after electro-acupuncture treatment in patients with osteoarthritis of the knee. Pain 2009 December 15;147(1-3):60-6. Off topic

(35) Akyol Y, Durmus D, Alayli G, Tander B, Bek Y, Canturk F, Sakarya ST. Does short-wave diathermy increase the effectiveness of isokinetic exercise on pain, function, knee muscle strength, quality of life, and depression in the patients with knee osteoarthritis?: A randomized controlled clinical study. Eur J Phys Rehabil Med 2010;46(3):325-36. Both groups exercised, No non-intervention control group

(36) Alayli G. Diz osteoartritinde aerobik egzersiz ve ev egzersiz programimn agri ve dizabilite uzerine etkileri (The effects of aerobic exercise and home exercise on pain and disability in patients with knee osteoarthritis) [Turkish]. Journal of Rheumatology and Medical Rehabilitation 2007. Met criteria but could not retrieve data

(37) Alexander G, Innes KE, Bourguignon C, Bovbjerg VE, Kulbok P, Taylor AG. Patterns of Yoga Practice and Physical Activity Following a Yoga Intervention for Adults With or at Risk for Type 2 Diabetes. Journal of Physical Activity & Health 2012 January;9(1):53-61. Longitudinal Study

(38) Ali A, Njike VY, Northrup V, Sabina AB, Williams AL, Liberti LS, Perlman AI, Adelson H, Katz DL. Intravenous micronutrient therapy (Myers' Cocktail) for fibromyalgia: A placebo-controlled pilot study. J Altern Complement Med 2009;15(3):247-57. Drug intervention study

(39) Allen KD, Bosworth HB, Brock DS, Chapman JG, Chatterjee R, Coffman CJ, Datta SK, Dolor RJ, Jeffreys AS, Juntilla KA, Kruszewski J, Marbrey LE, McDuffie J, Oddone EZ, Sperber N, Sochacki MP, Stanwyck C, Strauss JL, Yancy WS. Patient and provider interventions for managing osteoarthritis in primary care: protocols for two randomized controlled trials. BMC Musculoskeletal Disorders 2012;13. Description versus conduct of study

(40) Allen SM, Ciambrone D, Welch LC. Stage of life course and social support as a mediator of mood state among persons with disability. Journal of Aging and Health 2000;12(3):318-41. Not a randomized controlled trial (RCT)

(41) Alnigenis MNY, Bradley JD, Wallick. Massage therapy in the management of fibromyalgia: a pilot study. J MUSCULOSKELET PAIN 2001. Not an exercise intervention study

(42) Altan L, Bingol U, Aykac M, Koc Z, Yurtkuran M. Investigation of the effects of pool-based exercise on fibromyalgia syndrome. Rheumatol Int 2004 September;24(5):272-7. No non-intervention control group

(43) Altan L, Korkmaz N, Bingol U, Gunay B. Effect of pilates training on people with fibromyalgia syndrome: a pilot study. Arch Phys Med Rehabil 2009 December;90(12):1983-8. Primary outcome(s) not assessed

(44) Altay F. Effects of TENS on pain, disability, quality of life and depression in patients with knee osteoarthritis. Turkish Journal of Rheumatology 2010. No non-intervention control group

(45) Analay Y, Ozcan E, Karan A, Diracoglu D, Aydin R. The effectiveness of intensive group exercise on patients with ankylosing spondylitis. Clin Rehabil 2003;17(6):631-6. ankylosing spondylitis participants

(46) Andersson M, Bagby JR, Dyrehag LE, Gottfries CG. Effects of staphylococcus toxoid vaccine on pain and fatigue in patients with fibromyalgia/chronic fatigue syndrome. Eur J Pain 1998;2(2):133-42. Drug intervention study

(47) Ang D, Kesavalu R, Lydon JR, Lane KA, Bigatti S. Exercise-based motivational interviewing for female patients with fibromyalgia: a case series. Clin Rheumatol 2007;26(11):1843-9. Behavior Modification Intervention

(48) Ang DC, Kaleth AS, Bigatti S, Mazzuca S, Saha C, Hilligoss J, Lengerich M, Bandy R. Research to Encourage Exercise for Fibromyalgia (REEF): Use of motivational interviewing design and method. Contemp Clin Trials 2011;32(1):59-68. Description versus conduct of study

(49) Annesi JJ. Effects of Cardiovascular Exercise Frequency and Duration on Depression and Tension Changes Over 10 weeks. European Journal of Sport Science 2003 August;3(4):1-12. Subjects (some or all) did not have arthritis or other disease of interest

(50) Apovian CM, Gokce N. Obesity and cardiovascular disease. Circulation 2012;125(9):1178-82. Case-Control / Case Study

(51) Arcos C, I, Castro-Sánchez AM, Matarán-Peñarrocha GA, Gutiérrez-Rubio AB, Ramos GE, Moreno LC. [Effects of aerobic exercise program and relaxation techniques on anxiety, quality of sleep, depression, and quality of life in patients with fibromyalgia: a randomized controlled trial]. Medicina clínica 2011;137:398-401. No exercise only group

(52) Ardic F, Ozgen M, Aybek H, Rota S, Cubukcu D, Gokgoz A. Effects of balneotherapy on serum IL-1, PGE2 and LTB4 levels in fibromyalgia patients. Rheumatol Int 2007 March;27(5):441-6. Not an exercise intervention study

(53) Arnold LM, Crofford LJ, Mease PJ, Burgess SM, Palmer SC, Abetz L, Martin SA. Patient perspectives on the impact of fibromyalgia. Patient Education & Counseling 2008 October;73(1):114-20. Not a randomized controlled trial (RCT)

(54) Arnold LM, Clauw D, Wang FJ, Ahl J, Gaynor PJ, Wohlreich MM. Flexible Dosed Duloxetine in the Treatment of Fibromyalgia: A Randomized, Double-blind, Placebo-controlled Trial. J Rheumatol 2010;37(12):2578-86. Drug intervention study

(55) Arnold LM, Chatamra K, Hirsch I, Stoker M. Safety and efficacy of esreboxetine in patients with fibromyalgia: An 8-week, multicenter, randomized, double-blind, placebo-controlled study. Clin Ther 2010;32(9):1618-32. Drug intervention study

(56) Arnold LM, Zlateva G, Sadosky A, Emir B, Whalen E. Correlations between Fibromyalgia Symptom and Function Domains and Patient Global Impression of Change: A Pooled Analysis of Three Randomized, Placebo-Controlled Trials of Pregabalin. Pain Med (USA) 2011;12(2):260-7. Secondary analysis

(57) Arnold LM, Wang F, Ahl J, Gaynor PJ, Wohlreich MM. Improvement in multiple dimensions of fatigue in patients with fibromyalgia treated with duloxetine: Secondary analysis of a randomized, placebo-controlled trial. Arthritis Res Ther 2011;13(3). Secondary analysis

(58) Arvold DS, Odean MJ, Dornfeld MP, Regal RR, Arvold JG, Karwoski GC, Mast DJ, Sanford PB, Sjoberg RJ. Correlation of Symptoms with Vitamin D Deficiency and Symptom Response to Cholecalciferol Treatment: A Randomized Controlled Trial. Endocrine Practice 2009;15(3):203-12. Off topic

(59) Ashworth NL, Chad KE, Harrison EL, Reeder BA, Marshall SC. Home versus center based physical activity programs in older adults. Cochrane Database of Systematic Reviews 2005. Review article

(60) Assis MR, Silva LE, Alves AM, Pessanha AP, Valim V, Feldman D, Neto TL, Natour J. A randomized controlled trial of deep water running: clinical effectiveness of aquatic exercise to treat fibromyalgia. Arthritis Rheum 2006 February 15;55(1):57-65. No non-intervention control group

(61) Astin JA, Berman BM, Bausell B, Lee WL, Hochberg M, Forys KL. The efficacy of mindfulness meditation plus Qigong movement therapy in the treatment of fibromyalgia: A randomized controlled trial. J Rheumatol 2003;30(10):2257-62. No exercise only group

(62) Atienza AA, Oliveira B, Fogg BJ, King AC. Using Electronic Diaries to Examine Physical Activity and Other Health Behaviors of Adults Age 50+. Journal of Aging & Physical Activity 2006 April;14(2):192-202. Off topic

(63) Axford J. Management of knee osteoarthritis in primary care: pain and depression are the major obstacles. J Psychosom Res 2008. Educational intervention

(64) Baker J, Meisner BA, Logan AJ, Kungl AM, Weir P. Physical Activity and Successful Aging in Canadian Older Adults. Journal of Aging & Physical Activity 2009 April;17(2):223-35. Survey or questionnaire

(65) Barlow JH, Wright CC, Williams B, Keat A. Work disability among people with ankylosing spondylitis. Arthritis & Rheumatism-Arthritis Care & Research 2001;45(5):424-9. Cross-sectional study

(66) Barlow JH, Turner AP, Wright CC. A randomized controlled study of the Arthritis Self-Management Programme in the UK. Health Educ Res 2000 December;15(6):665-80. Longitudinal Study

(67) Bartels EM, Lund H, Hagen KB, Dagfinrud H, Christensen R, Danneskiold SB. Aquatic exercise for the treatment of knee and hip osteoarthritis. Cochrane Database of Systematic Reviews 2007. Review article

(68) Bateham A. Functional social support and well-being in adults with and without fibromyalgia. United States -- California: California State University, Fullerton; 2011. Not a randomized controlled trial (RCT)

(69) Bayliss EA, Ellis JL, Steiner JF. Barriers to self-management and quality-of-life outcomes in seniors with multimorbidities. Annals of Family Medicine 2007;5(5):395-402. Cross-sectional study

(70) Bayliss EA, Edwards AE, Steiner JF, Main DS. Processes of care desired by elderly patients with multimorbidities. Family practice 2008;25(4):287-93. Not a randomized controlled trial (RCT)

(71) Bedard AM. The experiences of older adults with chronic physical impairments in a six-month exercise program: Effects on quality of life, self-esteem and self-efficacy. Canada: The University of Manitoba (Canada); 1998. Case-Control / Case Study

(72) Beissner K, Parker SJ, Henderson J, Pal A, Iannone L, Reid MC. A Cognitive-Behavioral Plus Exercise Intervention for Older Adults With Chronic Back Pain: Race/Ethnicity Effect? Journal of Aging & Physical Activity 2012 April;20(2):246-65. Not a randomized controlled trial (RCT)

(73) Bellometti S, Galzigna L. Function of the hypothalamic adrenal axis in patients with fibromyalgia syndrome undergoing mud-pack treatment. International Journal of Clinical Pharmacology Research 1999;19(1):27-33. Off topic

(74) Belza B, Topolski T, Kinne S, Patrick DL, Ramsey SD. Does adherence make a difference? Results from a community-based aquatic exercise program. Nursing Research 2002 September;51(5):285-91. Same subjects as another study already included

(75) Bennett R, Russell IJ, Choy E, Spaeth M, Mease P, Kajdasz D, Walker D, Wang F, Chappell A. Evaluation of Patient-Rated Stiffness Associated With Fibromyalgia: A Post-Hoc Analysis of 4 Pooled, Randomized Clinical Trials of Duloxetine. Clin Ther 2012;34(4):824-37. Drug intervention study

(76) Bennett RM, Burckhardt CS, Clark SR, O'Reilly CA, Wiens AN, Campbell SM. Group treatment of fibromyalgia: A 6 month outpatient program. J Rheumatol 1996;23(3):521-8. Behavior Modification Intervention

(77) Berger A, Dukes EM, Oster G. Clinical characteristics and economic costs of patients with painful neuropathic disorders. J Pain 2004;5(3):143-9. Off topic

(78) Bieber C. (Shared decision making (SDM) with chronic pain patients. The patient as a partner in the medical decision making process) [German]. Gesundheitsschutz 2004. Not an exercise intervention study

(79) Bircan C, Karasel SA, Akgun B, El O, Alper S. Effects of muscle strengthening versus aerobic exercise program in fibromyalgia. Rheumatol Int 2008 April;28(6):527-32. No non-intervention control group

(80) Bishop MD, Meuleman J, Robinson M, Light KE. Influence of pain and depression on fear of falling, mobility, and balance in older male veterans. Journal of Rehabilitation Research & Development 2007 September;44(5):675-83. Off topic

(81) Blumenthal JA, Babyak MA, Moore KA, Craighead E, Herman S, Khatri P, Waugh R, Napolitano MA, Forman LM, Appelbaum M, Doraiswamy PM, Krishnan KR. Effects of exercise training on older patients with major depression. Archives of Internal Medicine 1999;159(19):2349-56. No non-intervention control group

(82) Bosch PR, Traustadottir T, Howard P, Matt KS. Functional and physiological effects of yoga in women with rheumatoid arthritis: a pilot study. Alternative Therapies in Health & Medicine 2009 July;15(4):24-31. Not a randomized controlled trial (RCT)

(83) Brattberg G. Connective tissue massage in the treatment of fibromyalgia. Eur J Pain 1999. Not an exercise intervention study

(84) Brazzelli M, Saunders DH, Greig CA, Mead GE. Physical fitness training for stroke patients. Cochrane Database of Systematic Reviews 2011. Review article

(85) Brenes GA, Williamson JD, Messier SP, Rejeski WJ, Pahor M, Ip E, Penninx BWJH. Treatment of minor depression in older adults: A pilot study comparing sertraline and exercise. Aging & Mental Health 2007 January;11(1):61-8. Subjects (some or all) did not have arthritis or other disease of interest

(86) Broderick JE, Junghaenel DU, Schneider S, Bruckenthal P, Keefe FJ. Treatment expectation for pain coping skills training: Relationship to osteoarthritis patients baseline psychosocial characteristics. Clin J Pain 2011;27(4):315-22. Survey or questionnaire

(87) Brosse AL, Sheets ES, Lett HS, Blumenthal JA. Exercise and the treatment of clinical depression in adults: recent findings and future directions. Sports Medicine 2002 September;32(12):741-60. Review article

(88) Brosseau L, MacLeay L, Welch V, Tugwell P, Wells GA. Intensity of exercise for the treatment of osteoarthritis. Cochrane Database of Systematic Reviews 2003. Review article

(89) Brosseau L, Wells GA, Tugwell P, Egan M, Wilson KG, Dubouloz CJ, Casimiro L, Robinson VA, McGowan J, Busch A, Poitras Sp, Moldofsky H, Harth M, Finestone HM, Nielson W, Haines-Wangda A, Russell-Doreleyers M, Lambert K, Marshall AD, Veilleux L. Ottawa Panel Evidence-Based Clinical Practice Guidelines for Strengthening Exercises in the Management of Fibromyalgia: Part 2. Physical Therapy 2008 July;88(7):873-86. Review article

(90) Brosseau L, Wells GA, Tugwell P, Egan M, Wilson KC, Dubouloz CJ, Casimiro L, Robinson VA, McGowan J, Busch A, Poitras Sp, Moldofsky H, Harth M, Finestone HM, Nielson W, Haines-Wangda A, Russell-Doreleyers M, Lambert K, Marshall AD, Veilleux L. Ottawa Panel Evidence-Based Clinical Practice Guidelines for Aerobic Fitness Exercises in the Management of Fibromyalgia: Part 1. Physical Therapy 2008 July;88(7):857-71. Review article

(91) Brosseau L, Wells GA, Tugwell P, Egan M, Dubouloz CJ, Casimiro L, Bugnariu N, Welch VA, De Angelis G, Francoeur L, Milne S, Loew L, McEwan J, Messier SP, Doucet E, Kenny GP, Prud'homme D, Lineker S, Bell M, Poitras Sp. Ottawa Panel Evidence-Based Clinical Practice Guidelines for the Management of Osteoarthritis in Adults Who Are Obese or Overweight. Physical Therapy 2011 June;91(6):843-61. Review article

(92) Brown AK, Liu-Ambrose T, Tate R, Lord SR. The effect of group-based exercise on cognitive performance and mood in seniors residing in intermediate care and self-care retirement facilities: a randomised controlled trial. British Journal of Sports Medicine 2009 August;43(8):608-14. Subjects (some or all) did not have arthritis or other disease of interest

(93) Brunton S, Wang FJ, Edwards SB, Crucitti AS, Ossanna MJ, Walker DJ, Robinson MJ. Profile of Adverse Events with Duloxetine Treatment A Pooled Analysis of Placebo-Controlled Studies. Drug Safety 2010;33(5):393-407. Drug intervention study

(94) Brus HL, Taal E, van de Laar MA, Rasker JJ, Wiegman O. Patient education and disease activity: a study among rheumatoid arthritis patients. Arthritis Care & Research 1997 October;10(5):320-4. Retrospective study

(95) Buckelew SP, Parker JC, Keefe FJ, Deuser WE, Crews TM, Conway R, Kay DR, Hewett JE. Self-efficacy and pain behavior among subjects with fibromyalgia. Pain 1994 December;59(3):377-84. Review article

(96) Busch AJ, Overend TJ, Schachter CL. Fibromyalgia treatment: the role of exercise and physical activity. International Journal of Clinical Rheumatology 2009. Review article

(97) Busch AJ, Schachter CL, Overend TJ, Peloso PM, Barber KA. Exercise for fibromyalgia: a systematic review. J Rheumatol 2008 June;35(6):1130-44. Review article

(98) Busch AJ, Barber-Karen AR, Overend TJ, Peloso-Paul MJ, Schachter CL. Exercise for treating fibromyalgia syndrome. Cochrane Database of Systematic Reviews 2007. Review article

(99) Buszewicz M, Rait G, Griffin M, Nazareth I, Patel A, Atkinson A, Barlow J, Haines A. Self management of arthritis in primary care: randomised controlled trial. British Medical Journal 2006;333(7574):879-882A. No exercise only group

(100) Cadmus L, Patrick MB, Maciejewski ML, Topolski T, Belza B, Patrick DL. Community-based aquatic exercise and quality of life in persons with osteoarthritis. Medicine & Science in Sports & Exercise 42[1], 8-15. 2010. 2010. Same subjects as another study already included,

(101) Calandre EP, Rodriguez-Claro ML, Rico-Villademoros F, Vilchez JS, Hidalgo J, Delgado-Rodriguez A. Effects of pool-based exercise in fibromyalgia symptomatology and sleep quality: a prospective randomised comparison between stretching and Ai Chi. Clinical & Experimental Rheumatology 2009 September;27(5:Suppl 56):S21-S28. No non-intervention control group

(102) Calandre EP, Rico-Villademoros F. The Role of Antipsychotics in the Management of Fibromyalgia. CNS Drugs 2012 February;26(2):135-53. Review article

(103) Calfas KJ, Kaplan RM, Ingram RE. One-year evaluation of cognitive-behavioral intervention in osteoarthritis. Arthritis Care & Research 1992 December;5(4):202-9. Behavior Modification Intervention

(104) Cameron ID, Murray GR, Gillespie LD, Robertson MC, Hill KD, Cumming RG, Kerse N. Interventions for preventing falls in older people in nursing care facilities and hospitals. Cochrane Database of Systematic Reviews 2010. Review article

(105) Cameron KA. Healthy aging: Programs that make a difference-Part 1 - First of a two-part series. Consult Pharm 2012;27(4):239-53. Review article

(106) Cancelliere C. Are workplace health promotion/wellness programs effective at improving presenteeism (on-the-job productivity) in workers? A systematic review and best evidence synthesis of the literature. Canada: Lakehead University (Canada); 2011. Off topic

(107) Cannon CP, Kumar A. Treatment of overweight and obesity: Lifestyle, pharmacologic, and surgical options. Clin Cornerstone 2009;9(4):55-71. Review article

(108) Cao H. Traditional Chinese medicine for treatment of fibromyalgia: a systematic review of randomized controlled trials. Journal of Alternative & Complementary Medicine 2010. Review article

(109) Carbonell-Baeza A, Aparicio VA, Martins-Pereira CM, Gatto-Cardia CM, Ortega FB, Huertas FJ, Tercedor P, Ruiz JR, Delgado-Fernandez M. Efficacy of Biodanza for treating women with fibromyalgia. Journal of Alternative & Complementary Medicine 2010 November;16(11):1191-200. Not a randomized controlled trial (RCT)

(110) Carbonell-Baeza A, Aparicio VA, Chillon P, Femia P, Delgado-Fernandez M, Ruiz JR. Effectiveness of multidisciplinary therapy on symptomatology and quality of life in women with fibromyalgia. Clinical & Experimental Rheumatology 2011 November;29(6:Suppl 69):Suppl-103. Not a randomized controlled trial (RCT)

(111) Carbonell-Baeza A, Aparicio VA, Ortega FB, Cuevas AM, Alvarez IC, Ruiz JR, Delgado-Fernandez M. Does a 3-month multidisciplinary intervention improve pain, body composition and physical fitness in women with fibromyalgia? British Journal of Sports Medicine 2011 December;45(15):1189-95. Not a randomized controlled trial (RCT)

(112) Carson JW, Carson KM, Jones KD, Bennett RM, Wright CL, Mist SD. A pilot randomized controlled trial of the Yoga of Awareness program in the management of fibromyalgia. Pain 2010;151:530-9. Multiple interventions

(113) Castro-Sanchez AM, Mataran-Penarrocha GA, Sanchez-Labraca N, Quesada-Rubio JM, Granero-Molina J, Moreno-Lorenzo C. A randomized controlled trial investigating the effects of craniosacral therapy on pain and heart rate variability in fibromyalgia patients. Clinical Rehabilitation 25[1], 25-35. 2011. 2011. Not an exercise intervention study,

(114) Cazzola M. Which kind of exercise is best in fibromyalgia therapeutic programmes? A practical review. Clinical and Experimental Rheumatology 2010. Review article

(115) Cedraschi C, Desmeules J, Rapiti E, Baumgartner E, Cohen P, Finckh A, Allaz AF, Vischer TL. Fibromyalgia: a randomised, controlled trial of a treatment programme based on self management. Annals of the Rheumatic Diseases 2004 March;63(3):290-6. No exercise only group

(116) Cella M, Sharpe M, Chalder T. Measuring disability in patients with chronic fatigue syndrome: reliability and validity of the Work and Social Adjustment Scale. J Psychosom Res 2011 September;71(3):124-8. Cohort Study

(117) Chai E, Horton JR. Managing pain in the elderly population: Pearls and pitfalls. Curr Pain Headache Rep 2010;14(6):409-17. Review article

(118) Chanou K, Gerodimos V, Karatrantou K, Jamurtas A. Whole-body vibration and rehabilitation of chronic diseases: A review of the literature. Journal of Sports Science & Medicine 2012 June;11(2):187-200. Review article

(119) Che S.Zin, Nissen LM, Smith MT, O'Callaghan JP, Moore BJ. An Update on the Pharmacological Management of Post-Herpetic Neuralgia and Painful Diabetic Neuropathy. CNS Drugs 2008 March;22(5):417-42. Review article

(120) Cheema BS, Lassere M, Shnier R, Singh MAF. Rotator Cuff Tear in an Elderly Woman Performing Progressive Resistance Training: Case Report from a Randomized Controlled Trial. Journal of Physical Activity & Health 2007 January;4(1):113-20. Case-Control / Case Study

(121) Chen KW, Hassett AL, Hou F, Staller J, Lichtbroun AS. A pilot study of external qigong therapy for patients with fibromyalgia. Journal of Alternative & Complementary Medicine 2006 November;12(9):851-6. Study less than 4 weeks

(122) Chen KW, Perlman A, Liao JG, Lam A, Staller J, Sigal LH. Effects of external qigong therapy on osteoarthritis of the knee. A randomized controlled trial. Clin Rheumatol 2008 December;27(12):1497-505. Study less than 4 weeks

(123) CHIUNG-JU LIU, LATHAM NANC. Can progressive resistance strength training reduce physical disability in older adults? A meta-analysis study. Disability & Rehabilitation 2011 January 15;33(2):87-97. Review article

(124) Cho HJ, Hotopf M, Wessely S. The placebo response in the treatment of chronic fatigue syndrome: A systematic review and meta-analysis. Psychosom Med 2005;67(2):301-13. Review article

(125) Chou KL, Chi I. Reciprocal relationship between pain and depression in elderly Chinese primary care patients. Int J Geriatr Psychiatry 2005;20(10):945-52. Not an exercise intervention study

(126) Christensen FB. Lumbar spinal fusion: Outcome in relation to surgical methods, choice of implant and postoperative rehabilitation. Acta Orthopaedica Scandinavica 2004 October 2;75:1-43. Off topic

(127) Clark DI, Downing N, Mitchell J, Coulson L, Syzpryt EP, Doherty M. Physiotherapy for anterior knee pain: a randomised controlled trial. Annals of the Rheumatic Diseases 2000;59(9):700-4. Subjects (some or all) did not have arthritis or other disease of interest

(128) Clauw DJ, Mease P, Palmer RH, Gendreau RM, Wang Y. Milnacipran for the Treatment of Fibromyalgia in Adults: A 15-Week, Multicenter, Randomized, Double-Blind, Placebo-Controlled, Multiple-Dose Clinical Trial. Clin Ther 2008;30(11):1988-2004. Drug intervention study

(129) Clauw DJ. Pharmacotherapy for patients with fibromyalgia. J Clin Psychiatry 2008;69(SUPPL. 2):25-9. Review article

(130) Collins E, O'Connell S, Jelinek C, Miskevics S, Budiman-Mak E. Evaluation of psychometric properties of Walking Impairment Questionnaire in overweight patients with osteoarthritis of knee. Journal of Rehabilitation Research & Development 2008 July;45(4):559-66. Inappropriate Outcomes

(131) Cotter KA, Sherman AM. Love Hurts: The Influence of Social Relations on Exercise Self-Efficacy for Older Adults With Osteoarthritis. Journal of Aging & Physical Activity 2008 October;16(4):465-83. Longitudinal Study

(132) Cotter KA, Lachman ME. No Strain, No Gain: Psychosocial Predictors of Physical Activity Across the Adult Lifespan. Journal of Physical Activity & Health 2010 September;7(5):584-94. Cross-sectional study

(133) Crawford-Faucher A. Exercise reduces depressive symptoms in patients with chronic illness. Am Fam Phys 2012;86(3):291. Review article

(134) Creavin ST, Dunn KM, Mallen CD, Nijrolder I, van der Windt DAWM. Co-occurrence and associations of pain and fatigue in a community sample of Dutch adults. Eur J Pain 2010;14(3):327-34. Survey or questionnaire

(135) Crosby J. Osteoarthritis: Managing without surgery. J Fam Pract 2009;58(7):354-61. Review article

(136) Crotty M, Prendergast J, Battersby MW, Rowett D, Graves SE, Leach G, Giles LC. Self-management and peer support among people with arthritis on a hospital joint replacement waiting list: a randomised controlled trial. Osteoarthritis & Cartilage 2009 November;17(11):1428-33. Not an exercise intervention study

(137) Da Costa D., Abrahamowicz M, Lowensteyn I, Bernatsky S, Dritsa M, Fitzcharles MA, Dobkin PL. A randomized clinical trial of an individualized home-based exercise programme for women with fibromyalgia. Rheumatology (Oxford) 2005 November;44(11):1422-7. Primary outcome(s) not assessed

(138) Da Costa D, Bernatsky S, Dritsa M, Clarke AE, Dasgupta K, Keshani A, Pineau C. Determinants of sleep quality in women with systemic lupus erythematosus. Arthritis & Rheumatism-Arthritis Care & Research 2005;53(2):272-8. Survey or questionnaire

(139) Da Costa D, Zummer M, Fitzcharles MA. Biopsychosocial determinants of physical and mental fatigue in patients with spondyloarthropathy. Rheumatol Int 2011;31(4):473-80. Survey or questionnaire

(140) da Silva TFG, Suda EY, Marculo CA, Paes FHS, Pinheiro GT. Comparacao dos efeitos da estimulacao eletrica nervosa transcutanea e da hidroterapia na dor, flexibilidade e qualidade de vida de pacientes com fibromialgia (Comparison of transcutaneous electrical nerve stimulation and hydrotherapy effects on pain, flexibility and quality of life in patients with fibromyalgia) [Portuguese]. Fisioterapia e Pesquisa [Physical Therapy and Research] 2008. No non-intervention control group

(141) da Silva GD, Lorenzi-Filho G, Lage LV. Effects of yoga and the addition of Tui Na in patients with fibromyalgia. J Altern Complement Med 2007 December;13(10):1107-13. No non-intervention control group

(142) Dagfinrud H, Kvien TK, Hagen KB. Physiotherapy interventions for ankylosing spondylitis. Cochrane Database of Systematic Reviews 2008;(1). Review article

(143) Dannecker EA, Knoll V, Robinson ME. Sex differences in muscle pain: self-care behaviors and effects on daily activities. J Pain 2008 March;9(3):200-9. Survey or questionnaire

(144) de Andrade SC, de Carvalho RF, Soares AS, de Abreu Freitas RP, de Medeiros Guerra LM, Vilar MJ. Thalassotherapy for fibromyalgia: a randomized controlled trial comparing aquatic exercises in sea water and water pool. Rheumatol Int 2008 December;29(2):147-52. Both groups exercised

(145) Der Ananian C, Wilcox S, Watkins K, Saunders RP, Evans AE. Factors Associated With Exercise Participation in Adults With Arthritis. Journal of Aging & Physical Activity 2008 April;16(2):125-43. Cross-sectional study

(146) di BP. Clinical evaluation of S-adenosyl-L-methionine versus transcutaneous electrical nerve stimulation in primary fibromyalgia. Current Therapeutic Research, Clinical and Experimental 1993. CT

(147) Diamond S, Borenstein D. Chronic low back pain in a working-age adult. Best Pract Res Clin Rheumatol 2006;20(4):707-20. Review article

(148) Digas G. New polymer materials in total hip arthroplasty. Acta Orthopaedica 2005 February 2;76:4-82. Off topic

(149) Diracoglu D, Baskent A, Celik A, Issever H, Aydin R. Long-term effects of kinesthesia/balance and strengthening exercises on patients with knee osteoarthritis: a one-year follow-up study. Journal of Back & Musculoskeletal Rehabilitation 21[4], 253-262. 2008. Both groups exercised,

(150) Donald F, Esdaile JM, Kimoff JR, Fitzcharles MA. Musculoskeletal complaints and fibromyalgia in patients attending a respiratory sleep disorders clinic. J Rheumatol 1996;23(9):1612-6. Off topic

(151) Donaldson MS, Speight N, Loomis S. Fibromyalgia syndrome improved using a mostly raw vegetarian diet: an observational study. BMC Complementary & Alternative Medicine 2001;1:7. Diet Intervention Study

(152) Donmez A, Karagulle MZ, Tercan N, Dinler M, Issever H, Karagulle M, Turan M. SPA therapy in fibromyalgia: a randomised controlled clinic study. Rheumatol Int 2005 December;26(2):168-72. Study less than 4 weeks

(153) Dunlop DD, Song J, Semanik PA, Sharma L, Chang RW. Physical Activity Levels and Functional Performance in the Osteoarthritis Initiative A Graded Relationship. Arthritis and Rheumatism 2011;63(1):127-36. Cross-sectional study

(154) Durmus D. Diz osteoartritli hastalarda biofeedback yardimli izometrik egzersiz ve elektrik stimulasyon programinin agri, anksiyete ve depresyon uzerine etkisi (Effects of biofeedback assisted isometric exercise and electrical stimulation on pain, anxiety and depression scores in knee osteoarthritis) [Turkish]. Turkiye Fiziksel Tip ve Rehabilitasyon Dergisi [Turkish Journal of Physical Medicine and Rehabilitation] 2005. No non-intervention control group

(155) Ekici G. Fibromiyaljili kadinlarda Pilates egzersizleri ve konnektif doku manipulasyonunun agri ve depresyon uzerine etkileri: rastgele kontrollu calisma (Effects of Pilates exercises and connective tissue manipulation on pain and depression in females with fibromyalgia: a randomized controlled trial) [Turkish]. Fizyoterapi Rehabilitasyon [Turkish Journal of Physiotherapy Rehabilitation] 2008. No non-intervention control group

(156) Ettinger WH, Burns R, Messier SP, Applegate W, Rejeski WJ, Morgan T, Shumaker S, Berry MJ, O'Toole M, Monu J, Craven T. A randomized trial comparing aerobic exercise and resistance exercise with a health education program in older adults with knee osteoarthritis. The Fitness Arthritis and Seniors Trial (FAST). JAMA : the journal of the American Medical Association 1997;277:25-31. Primary outcome(s) not assessed

(157) Evans S, Moieni M, Taub R, Subramanian SK, Tsao JC, Sternlieb B, Zeltzer LK. Iyengar yoga for young adults with rheumatoid arthritis: results from a mixed-methods pilot study. Journal of Pain & Symptom Management 2010 May;39(5):904-13. No control group (NC)

(158) Evcik D, Kizilay B, Gokcen E. The effects of balneotherapy on fibromyalgia patients. Rheumatol Int 2002 June;22(2):56-9. Study less than 4 weeks

(159) Evcik D, Yigit I, Pusak H, Kavuncu V. Effectiveness of aquatic therapy in the treatment of fibromyalgia syndrome: a randomized controlled open study. Rheumatol Int 2008 July;28(9):885-90. No non-intervention control group

(160) Evers AW, Kraaimaat FW, van Riel PL, de Jong AJ. Tailored cognitive-behavioral therapy in early rheumatoid arthritis for patients at risk: a randomized controlled trial. Pain 2002. Not an exercise intervention study

(161) Falcao DM. Cognitive behavioral therapy for the treatment of fibromyalgia syndrome: a randomized controlled trial. J MUSCULOSKELET PAIN 2008. Not an exercise intervention study

(162) Felson DT. Osteoarthritis of the knee. New Engl J Med 2006;354(8):841-8. Review article

(163) Ferraccioli G. EMG-biofeedback training in fibromyalgia syndrome. The Journal of Rheumatology 1987. Not an exercise intervention study

(164) Field T. Fibromyalgia pain and substance P decrease and sleep improves after massage therapy. Journal of Clinical Rheumatology 2002. Not an exercise intervention study

(165) Field T. Movement and massage therapy reduce fibromyalgia pain. Journal of Bodywork and Movement Therapies 2003. Study less than 4 weeks

(166) Finset A, Wigers SH, Gotestam KG. Depressed mood impedes pain treatment response in patients with fibromyalgia. J Rheumatol 2004 May;31(5):976-80. Same subjects as another study already included

(167) Fontaine KR, Conn L, Clauw DJ. Effects of lifestyle physical activity in adults with fibromyalgia: results at follow-up. JCR: Journal of Clinical Rheumatology 2011 March;17(2):64-8. Follow-up Study

(168) Fontaine KR, Haaz S. Effects of Lifestyle Physical Activity on Health Status, Pain, and Function in Adults with Fibromyalgia Syndrome. J MUSCULOSKELET PAIN 2007 March;15(1):3-9. Primary outcome(s) not assessed

(169) Forbes D, Chalmers A. Fibromyalgia: revisiting the literature. Journal of the Canadian Chiropractic Association 2004 June;48(2):119-31. Review article

(170) Fox M. The association between self-efficacy for exercise and physical activity level on people age 50 and over with fibromyalgia. United States -- California: California State University, Fullerton; 2009. Cross-sectional study

(171) Francisco Filipe Marmeleira J, de Melo FMS, Tlemcani M, Adriano Bandeira Godinho M. Exercise Can Improve Speed of Behavior in Older Drivers. Journal of Aging & Physical Activity 2011 January;19(1):48-61. Primary outcome(s) not assessed

(172) FREEDMAN VA, HODGSON NANC, LYNN JOAN, SPILLMAN BC, WAIDMANN TIMO, WILKINSON AM, WOLF DA. Promoting Declines in the Prevalence of Late-Life Disability: Comparisons of Three Potentially High-Impact Interventions. Milbank Quarterly 2006 September;84(3):493-520. Review article

(173) French HP, Cusack T, Brennan A, White B, Gilsenan C, Fitzpatrick M, O'Connell P, Kane D, Fitzgerald O, McCarthy GM. Exercise and manual physiotherapy arthritis research trial (EMPART): a multicentre randomised controlled trial. BMC Musculoskeletal Disorders 2009;10:9. Description versus conduct of study

(174) French SL. The self-management of osteoarthritis in older women: A study of the efficacy of the Arthritis Self-Management Program and of the factors influencing outcomes. US: ProQuest Information & Learning; 1996. Not a randomized controlled trial (RCT) Educational intervention

(175) Friedberg F, Sohl SJ. Longitudinal change in chronic fatigue syndrome: what home-based assessments reveal. Journal of Behavioral Medicine 2009;32(2):209-18. Prospective Study

(176) Friedberg MW. Pilot study suggests temporary efficacy of pilates for treating fibromyalgia: Commentary. J Clin Outcomes Manage 2010;17(8): 348-349. Review article

(177) Friedrich M, Hahne J, Wepner F. A Controlled Examination of Medical and Psychosocial Factors Associated With Low Back Pain in Combination With Widespread Musculoskeletal Pain. Physical Therapy 2009;89(8):786-803. Off topic

(178) Frost SS, Goins RT, Hunter RH, Hooker SP, Bryant LL, Kruger J, Pluto D. Effects of the Built Environment on Physical Activity of Adults Living in Rural Settings. American Journal of Health Promotion 2010 March;24(4):267-83. Review article

(179) Ga H, Koh HJ, Choi JH, Kim CH. Intramuscular and nerve root stimulation vs lidocaine injection to trigger points in myofascial pain syndrome. Journal of Rehabilitation Medicine 2007 May;39(5):374-8. Drug intervention study

(180) Gamber RG, Shores JH, Russo DP, Jimenez C, Rubin BR. Osteopathic manipulative treatment in conjunction with medication relieves pain associated with fibromyalgia syndrome: results of a randomized clinical pilot project. Journal of the American Osteopathic Association 2002 June;102(6):321-5. Not an exercise intervention study

(181) Gao Y, Ning GA, Jia WP, Zhou ZG, Xu ZR, Liu ZM, Liu C, Ma JH, Li QA, Cheng LL, Wen CY, Zhang SY, Zhang Q, Desaiah D, Skljarevski V. Duloxetine versus placebo in the treatment of patients with diabetic neuropathic pain in China. Chinese Medical Journal 2010;123(22):3184-92. Drug intervention study

(182) Garcia-Campayo J, Magdalena J, Magallon R, Fernandez-Garcia E, Salas M, Andres E. A meta-analysis of the efficacy of fibromyalgia treatment according to level of care. Arthritis Research & Therapy 2008;10(4). Review article

(183) Gardiner RL. Psychological and physiological responses to prescribed versus preferred exercise intensity in clients with fibromyalgia. US: ProQuest Information & Learning; 1998. Not a randomized controlled trial (RCT)

(184) Gillespie LD, Robertson MC, Gillespie WJ, Sherrington C, Gates S, Clemson LM, Lamb SE. Interventions for preventing falls in older people living in the community. Cochrane Database of Systematic Reviews 2012. Review article

(185) Gleberzon BJ. Chiropractic care of the older person: developing and evidence-based approached. Journal of the Canadian Chiropractic Association 2001 September;45(3):156-71. Review article

(186) Gowans SE, Dehueck A, Voss S, Richardson M. A randomized, controlled trial of exercise and education for individuals with fibromyalgia. Arthritis Care Res 1999;12(2):120-8. Multiple interventions

(187) Gowans SE, Dehueck A, Abbey SE. Measuring exercise-induced mood changes in fibromyalgia: a comparison of several measures. Arthritis Rheum 2002 December 15;47(6):603-9. Off topic

(188) Gowans SE, Dehueck A, Voss S, Silaj A, Abbey SE. Six-month and one-year followup of 23 weeks of aerobic exercise for individuals with fibromyalgia. Arthritis Rheum 2004 December 15;51(6):890-8. Follow-up Study

(189) Grace SL, Krepostman S, Brooks D, Arthur H, Scholey P, Suskin N, Jaglal S, Abramson BL, Stewart DE. Illness perceptions among cardiac patients: Relation to depressive symptomatology and sex. J Psychosom Res 2005;59(3):153-60. Subjects (some or all) did not have arthritis or other disease of interest

(190) Grassetto M, Varotto A. Primary fibromyalgia is responsive to S-adenosyl-L-methionine. CURR THER RES CLIN EXP 1994;55(7):797-806. Drug intervention study

(191) Gregg EW, Mangione CM, Cauley JA, Thompson TJ, Schwartz AV, Ensrud KE, Nevitt MC, Study of Osteoporotic Fractures Research Group. Diabetes and incidence of functional disability in older women. Diabetes Care 2002 January;25(1):61-7. Cohort Study

(192) Gusi N, Tomas-Carus P, Hakkinen A, Hakkinen K, Ortega-Alons A. Exercise in waist-high warm water decreases pain and improves health-related quality of life and strength in the lower extremities in women with fibromyalgia. Arthritis & Rheumatism-Arthritis Care & Research 2006;55(1):66-73. Primary outcome(s) not assessed [Scale used (EQ-5D), assessed anxiety or depression, not just depression)

(193) Gusi N, Reyes MC, Gonzalez-Guerrero JL, Herrera E, Garcia JM. Cost-utility of a walking programme for moderately depressed, obese, or overweight elderly women in primary care: a randomised controlled trial. BMC Public Health 2008;8:231. Primary outcome(s) not assessed, Inappropriate Outcomes

(194) Gustafsson M, Ekholm J, Broman L. Effects of a multiprofessional rehabilitation programme for patients with fibromyalgia syndrome. J Rehabil Med 2002 May;34(3):119-27. Not a randomized controlled trial (RCT)

(195) Hauser W, Bernardy K, Arnold B, Offenbacher M, Schiltenwolf M. Efficacy of multicomponent treatment in fibromyalgia syndrome: A meta-analysis of randomized controlled clinical trials. Arthritis Care Res 2009;61(2):216-24. Review article

(196) Hagg O, Fritzell P, Nordwall A. Characteristics of patients with chronic low back pain selected for surgery - A comparison with the general population reported from the Swedish lumbar spine study. Spine 2002;27(11):1223-30. Off topic

(197) Hagg O, Burckhardt C, Firtzell P, Nordwall A. Quality of life in chronic low back pain: A comparison with fibromyalgia and the general population. J MUSCULOSKELET PAIN 2003;11(1):31-8. Survey or questionnaire

(198) Hakkinen K, Pakarinen A, Hannonen P, Hakkinen A, Airaksinen O, Valkeinen H, Alen M. Effects of strength training on muscle strength, cross-sectional area, maximal electromyographic activity, and serum hormones in premenopausal women with fibromyalgia. J Rheumatol 2002 June;29(6):1287-95. Primary outcome(s) not assessed

(199) Hammond A, Bryan J, Hardy A. A lifestyle management for arthritis programme: short-term outcomes for people with inflammatory arthritis. Rheumatology 43, ii47. 2004. Abstract,

(200) Hammond A, Freeman K. Community patient education and exercise for people with fibromyalgia: a parallel group randomized controlled trial. Clin Rehabil 2006 October;20(10):835-46. Educational intervention

(201) Hanumanthu VS. Assessment of an exercise program on population with chronic medical conditions. United States -- Nebraska: University of Nebraska at Omaha; 2008. Not a randomized controlled trial (RCT)

(202) Hargrove JB, Bennett RM, Simons DG, Smith SJ, Nagpal S, Deering DE. A randomized placebo-controlled study of noninvasive cortical electrostimulation in the treatment of fibromyalgia patients. Pain Med (USA) 2012 January;13(1):115-24. Electrical Stimulation

(203) Harper A. Obesity: Is it important in the elderly? CME J Geriatr Med 2003;5(1):24-7. Review article

(204) Harrison SA, Hamzeh FM, Han J, Pandya PK, Sheikh MY, Vierling JM. Chronic hepatitis C genotype 1 patients with insulin resistance treated with pioglitazone and peginterferon alpha-2a plus ribavirin. Hepatology 2012;56(2):464-73. Drug intervention study

(205) Hassett AL, Radvanski DC, Vaschillo EG, Vaschillo B, Sigal LH, Karavidas MK, Buyske S, Lehrer PM. A pilot study of the efficacy of heart rate variability (HRV) biofeedback in patients with fibromyalgia. Appl Psychophysiol Biofeedback 2007;32(1):1-10. Not an exercise intervention study

(206) Haupt M, Millen S, Janner M, Falagan D, Fischer-Betz R, Schneider M. Improvement of coping abilities in patients with systemic lupus erythematosus: a prospective study. Annals of the Rheumatic Diseases 2005;64(11):1618-23. Educational intervention

(207) Hauser W, Schmutzer G, Brahler E, Glaesmer H. A Cluster Within the Continuum of Biopsychosocial Distress Can Be Labeled "Fibromyalgia Syndrome" - Evidence from a Representative German Population Survey. J Rheumatol 2009;36(12):2806-12. Survey or questionnaire

(208) Hauser W, Bernardy K, Arnold B, Offenbacher M, Schiltenwolf M. Efficacy of multicomponent treatment in fibromyalgia syndrome: a meta-analysis of randomized controlled clinical trials. Arthritis Rheum 2009;61(2):216-24. Review article

(209) Hauser W. Efficacy of different types of aerobic exercise in fibromyalgia syndrome: a systematic review and meta-analysis of randomised controlled trials. Arthritis Research & Therapy 2010. Review article

(210) Haworth J, Young C, Thornton E. The effects of an 'exercise and education' programme on exercise self-efficacy and levels of independent activity in adults with acquired neurological pathologies: an exploratory, randomized study. Clin Rehabil 2009 April;23(4):371-83. Subjects (some or all) did not have arthritis or other disease of interest

(211) Hegerl U, Mergl R, Quail D, Schneider E, Hundemer HP, Linden M. Does Pain Improve Earlier than Mood in Depressed Patients with Painful Physics Symptoms Treated with Duloxetine? Pharmacopsychiatry 2012;45(3):114-8. Drug intervention study

(212) Hennard J. A protocol and pilot study for managing fibromyalgia with yoga and meditation. International Journal of Yoga Therapy (21):109-21, 2011 2011;(21):109-21. Not a randomized controlled trial (RCT)

(213) Henness S, Perry CM. Orlistat: A Review of its Use in the Management of Obesity. Drugs 2006 June 15;66(12):1625-56. Review article

(214) Hergenroeder AL, Wert DM, Hile ES, Studenski SA, Brach JS. Association of Body Mass Index With Self-Report and Performance-Based Measures of Balance and Mobility. Physical Therapy 2011 August;91(8):1223-34. Cross-sectional study

(215) Horven Wigers S, Stiles TC, Vogel PA. Effects of aerobic exercise versus stress management treatment in fibromyalgia. SCAND J RHEUMATOL 1996;25(2):77-86. Duplicate

(216) Howe TE, Rochester L, Neil F, Skelton DA, Ballinger C. Exercise for improving balance in older people. Cochrane Database of Systematic Reviews 2011. Review article

(217) Hughes SL, Seymour RB, Campbell RT, Desai P, Huber G, Chang HJ. Fit and Strong!: bolstering maintenance of physical activity among older adults with lower-extremity osteoarthritis. American Journal of Health Behavior 2010 November;34(6):750-63. No comparative control group

(218) Huiskes C. Het effect van gedragstherapie en ergotherapie bij patienten met reumatoide arthritis (The effect of cognitive behavior therapy and occupational therapy in patients with rheumatoid arthritis) [Dutch]. Gedragstherapie 1991. Behavior Modification Intervention

(219) Hurley BF, Hanson ED, Sheaff AK. Strength Training as a Countermeasure to Aging Muscle and Chronic Disease. Sports Medicine 2011 April;41(4):289-306. Review article

(220) Hurley MV, Walsh NE, Mitchell HL, Pimm TJ, Patel A, Williamson E, Jones RH, Dieppe PA, Reeves BC. Clinical effectiveness of a rehabilitation program integrating exercise, self-management, and active coping strategies for chronic knee pain: a cluster randomized trial. Arthritis Rheum 2007 October 15;57(7):1211-9. Inappropriate Population

(221) Jacobi EM. The efficacy of the bonny method of Guided Imagery and Music as experiential therapy in the primary care of persons with rheumatoid arthritis. US: ProQuest Information & Learning; 1995. Not an exercise intervention study

(222) Jacobs JW, Geenen R, Evers AW, van Jaarsveld CH, Kraaimaat FW, Bijlsma JW. Short term effects of corticosteroid pulse treatment on disease activity and the wellbeing of patients with active rheumatoid arthritis. Annals of the Rheumatic Diseases 2001 January;60(1):61-4. Drug intervention study

(223) Jang Z-Y. (Combination of acupuncture, cupping and medicine for treatment of fibromyalgia syndrome: a multi-central randomized controlled trial) [Chinese - simplified characters]. Zhongguo Zhen Jiu [Chinese Acupuncture & Moxibustion] 2010. Not an exercise intervention study

(224) Jenkinson CM, Doherty M, Avery AJ, Read A, Taylor MA, Sach TH, Silcocks P, Muir KR. Effects of dietary intervention and quadriceps strengthening exercises on pain and function in overweight people with knee pain: Randomised controlled trial. BMJ (Online) 2009;339(7721):606-9. Subjects (some or all) did not have arthritis or other disease of interest

(225) Jenkinson CM, Doherty M, Avery AJ, Read A, Taylor MA, Sach TH, Silcocks P, Muir KR. Effects of dietary intervention and quadriceps strengthening exercises on pain and function in overweight people with knee pain: randomised controlled trial.[Erratum appears in BMJ. 2010;340:c2088]. BMJ 2009;339:b3170. Duplicate

(226) Jentoft ES, Kvalvik AG, Mengshoel AM. Effects of pool-based and land-based aerobic exercise on women with fibromyalgia/chronic widespread muscle pain. Arthritis Care Res 2001;45(1):42-7. No control group (NC)

(227) Jessep SA, Walsh NE, Ratcliffe J, Hurley MV. Long-term clinical benefits and costs of an integrated rehabilitation programme compared with outpatient physiotherapy for chronic knee pain. Physiotherapy 2009 June;95(2):94-102. No control group (NC)

(228) Johannesson E, Simren M, Strid H, Bajor A, Sadik R. Physical activity improves symptoms in irritable bowel syndrome: a randomized controlled trial. American Journal of Gastroenterology 2011 May;106(5):915-22. Subjects (some or all) did not have arthritis or other disease of interest

(229) Jones KD, Burckhardt CS, Clark SR, Bennett RM, Potempa KM. A Randomized controlled trial of muscle strengthening versus flexibility training in fibromyalgia. J Rheumatol 2002;29(5):1041-8. No comparative control group, Both groups exercised

(230) Jones KD, Deodhar AA, Burckhardt CS, Perrin NA, Hanson GC, Bennett RM. A combination of 6 months of treatment with pyridostigmine and triweekly exercise fails to improve insulin-like growth factor-I levels in fibromyalgia, despite improvement in the acute growth hormone response to exercise. J Rheumatol 2007 May;34(5):1103-11. Drug intervention study

(231) Jordan JL, Holden MA, Mason-Elizabeth EJ, Foster NE. Interventions to improve adherence to exercise for chronic musculoskeletal pain in adults. Cochrane Database of Systematic Reviews 2010. Review article

(232) Jorge LL, Tomikawa LCO, Juca SSH. Effects of a multidisciplinary rehabilitation program for men with fibromyalgia: controlled randomized study. Acta Fisiatrica 2007. Multiple interventions

(233) Joyce J, Hotopf M, Wessely S. The prognosis of chronic fatigue and chronic fatigue syndrome: A systematic review. Qjm-Monthly Journal of the Association of Physicians 1997;90(3):223-33. Review article

(234) Kang JH, Lin HC. Comorbidities in Patients with Primary Sjogren's Syndrome: A Registry-based Case-control Study. J Rheumatol 2010;37(6):1188-94. Case-Control / Case Study

(235) Kanning M, Schlicht W. Be Active and Become Happy: An Ecological Momentary Assessment of Physical Activity and Mood. Journal of Sport & Exercise Psychology 2010 April;32(2):253-61. Cross-sectional study

(236) Kaplan S, Kozin. A controlled study of group counseling in rheumatoid arthritis. The Journal of Rheumatology 1981. Counseling Intervention

(237) Karmisholt K, Gotzsche PC. Physical activity for secondary prevention of disease. Systematic reviews of randomised clinical trials. Dan Med Bull 2005;52(2):90-4. Review article

(238) Katalinic OM, Harvey LA, Herbert RD, Moseley AM, Lannin NA, Schurr K. Stretch for the treatment and prevention of contractures. Cochrane Database of Systematic Reviews 2010. Review article

(239) Katz JD, Shah T. Persistent pain in the older adult: What should we do now in light of the 2009 American Geriatrics Society Clinical Practice Guideline? Pol Arch Med Wewn 2009;119(12):795-800. Review article

(240) Kesiktas N, Karagulle Z, Erdogan N, Yazicioglu K, Yilmaz H, Paker N. The efficacy of balneotherapy and physical modalities on the pulmonary system of patients with fibromyalgia. Journal of Back & Musculoskeletal Rehabilitation 2011;24(1):57-65. Study less than 4 weeks

(241) Khan FM, Williams PI. Double-blind comparison of etodolac SR and diclofenac SR in the treatment of patients with degenerative joint disease of the knee. Current Medical Research & Opinion 1992;13(1):1-12. Drug intervention study

(242) King SJ, Wessel J, Bhambhani Y, Sholter D, Maksymowych W. The effects of exercise and education, individually or combined, in women with fibromyalgia. J Rheumatol 2002 December;29(12):2620-7. Primary outcome(s) not assessed

(243) Kingsley JD, Panton LB, Toole T, Sirithienthad P, Mathis R, McMillan V. The effects of a 12-week strength-training program on strength and functionality in women with fibromyalgia. Arch Phys Med Rehabil 2005 September;86(9):1713-21. Primary outcome(s) not assessed

(244) Kivitz A, Ma C, Ahdieh H, Galer BS. A 2-week, multicenter, randomized, double-blind, placebo-controlled, dose-ranging, phase III trial comparing the efficacy of oxymorphone extended release and placebo in adults with pain associated with osteoarthritis of the hip or knee. Clin Ther 2006;28(3):352-64. Study less than 4 weeks

(245) Korszun A, Young EA, Engleberg NC, Brucksch CB, Greden JF, Crofford LA. Use of actigraphy for monitoring sleep and activity levels in patients with fibromyalgia and depression. J Psychosom Res 2002;52(6):439-43. Not an exercise intervention study

(246) Kravitz HM, Katz RS, Helmke N, Jeffriess H, Bukovsky J, Fawcett J. Alprazolam and ibuprofen in the treatment of fibromyalgia - Report of a double-blind placebo-controlled study. J MUSCULOSKELET PAIN 1994;2(1):3-27. Drug intervention study

(247) Kreider RB, Almada AL, Antonio J, Broeder C, Earnest C, Greenwood L, Greenwood M, Incledon T, Kalman DS, Kerksick C, Kleiner SM, Lowery LM, Leutholtz B, Mendel R, Rasmussen CJ, Stout JR, Weir JP, Willoughby DS, Ziegenfuss TN. Exercise and Sport Nutrition: A Balanced Perspective for Exercise Physiologists. Professionalization of Exercise Physiology 2003 August;6(8):1. Review article

(248) Kreider RB, Wilborn CD, Taylor L, Campbell B, Almada AL, Collins R, Cooke M, Earnest CP, Greenwood M, Kalman DS, Kerksick CM, Kleiner SM, Leutholtz B, Lopez H, Lowery LM, Mendel R, Smith A, Spano M, Wildman R, Willoughby DS. ISSN exercise & sport nutrition review: research & recommendations. Journal of the International Society of Sports Nutrition 2010 January;7:7-49. Review article

(249) Kujala UM. Evidence on the effects of exercise therapy in the treatment of chronic disease. British Journal of Sports Medicine 2009 August;43(8):550-5. Review article

(250) Kurzeja R. (Fibromyalgia: comparison of whole-body-cryotherapy with two classical thermotherapy methods) [German]. Aktuelle Rheumatologie 2003. Not an exercise intervention study

(251) Lange AK, Fiatarone Singh MA, Smith RM, Foroughi N, Baker MK, Shnier R, Vanwanseele B. Degenerative meniscus tears and mobility impairment in women with knee osteoarthritis. Osteoarthritis Cartilage 2007;15(6):701-8. Cross-sectional study

(252) Lange AK, Vanwanseele B, Fiatarone-Singh MA. Strength training for treatment of osteoarthritis of the knee: a systematic review (Structured abstract). Arthritis and Rheumatism 2008;59:1488-94. Review article

(253) Lange AK, Vanwanseele B, Foroughi N, Baker MK, Shnier R, Smith RM, Singh MAF. Resistive Exercise for Arthritic Cartilage Health (REACH): A randomized double-blind, sham-exercise controlled trial. BMC Geriatr 2009;9(1). Description versus conduct of study

(254) Lange G, Janal MN, Maniker A, Fitzgibbons J, Fobler M, Cook D, Natelson BH. Safety and efficacy of vagus nerve stimulation in fibromyalgia: a phase I/II proof of concept trial. Pain Med (USA) 2011 September;12(9):1406-13. Not a randomized controlled trial (RCT)

(255) Lange M. Mittelfristige effekte einer multimodalen behandlung bei patienten mit fibromyalgiesyndrom: ergebnisse einer kontrollierten wirksamkeitsstudie (Medium-term effects of a multimodal therapy on patients with fibromyalgia. Results of a controlled efficacy study) [German]. Der Schmerz 2011. Behavior Modification Intervention

(256) Latimer AE, Martin Ginis KA, Hicks AL, McCartney N. An examination of the mechanisms of exercise-induced change in psychological well-being among people with spinal cord injury. Journal of Rehabilitation Research & Development 2004 September;41(5):643-51. Subjects (some or all) did not have arthritis or other disease of interest

(257) Lawrence VJ, Kopelman PG. Medical consequences of obesity. Clin Dermatol 2004;22(4 SPEC. ISS.):296-302. Review article

(258) Lee E.N. [Effects of a tai-chi program on pain, sleep disturbance, mood and fatigue in rheumatoid arthritis patients] (in Korean). Journal of Muscle and Joint Health 12[1], 57-68. 2005. Primary outcome(s) not assessed,

(259) Lee MS, Pittler MH, Ernst. Tai Chi for rheumatoid arthritis: systematic review [with consumer summary]. Rheumatology 2007. Review article

(260) Lee D. Adrenal fatigue syndrome: A project report. United States -- California: California State University, Long Beach; 2009. Review article

(261) Leibing E, Pfingsten. Cognitive-behavioral treatment in unselected rheumatoid arthritis outpatients. The Clinical Journal of Pain 1999. Behavior Modification Intervention

(262) Lempp H, Ibrahim F, Shaw T, Hofmann D, Graves H, Thornicroft G, Scott I, Kendrick T, Scott DL. Comparative quality of life in patients with depression and rheumatoid arthritis. International Review of Psychiatry 2011;23(1):118-24. Review article

(263) Lemstra M, Olszynski WP. The effectiveness of multidisciplinary rehabilitation in the treatment of fibromyalgia: A randomized controlled trial. Clin J Pain 2005;21(2):166-74. Multiple interventions

(264) Li JX, Hong Y, Chan KM. Tai chi: physiological characteristics and beneficial effects on health. / Tai chi: caracteristiques physiologiques et effets benefiques sur la sante. British Journal of Sports Medicine 2001 June;35(3):148-56. Review article

(265) Lim HJ, Moon YI, Lee MS. Effects of home-based daily exercise therapy on joint mobility, daily activity, pain, and depression in patients with ankylosing spondylitis. Rheumatol Int 2005;25(3):225-9. Subjects (some or all) did not have arthritis or other disease of interest

(266) Lin EHB, Katon W, Von Korff M, Tang LQ, Williams JW, Kroenke K, Hunkeler E, Harpole L, Hegel M, Arean P, Hoffing M, Della Penna R, Langston C, Unutzer J. Effect of improving depression care on pain and functional outcomes among older adults with arthritis - A randomized controlled trial. Jama-Journal of the American Medical Association 2003;290(18):2428-34. Drug intervention study

(267) Lin EHB, Tang LQ, Katon W, Hegel MT, Sullivan MD, Unutzer J. Arthritis pain and disability: response to collaborative depression care. General Hospital Psychiatry 2006;28(6):482-6. Drug intervention study

(268) Lin EHB. Depression and Osteoarthritis. AM J MED 2008;121(11):16-9. Review article

(269) Linder JA, Singer DE. Health-related quality of life of adults with upper respiratory tract infections. Journal of General Internal Medicine 2003;18(10):802-7. Subjects (some or all) did not have arthritis or other disease of interest

(270) Liu Cj, Latham NK. Progressive resistance strength training for improving physical function in older adults. Cochrane Database of Systematic Reviews 2009. Review article

(271) Lobo ED, Quinlan T, O'Brien L, Knadler MP, Heathman M. Population Pharmacokinetics of Orally Administered Duloxetine in Patients Implications for Dosing Recommendation. Clinical Pharmacokinetics 2009;48(3):189-97. Drug intervention study

(272) Lobstein T, Baur L, Uauy R. Obesity in children and young people: A crisis in public health. Obes Rev Suppl 2004;5(1):4-104. Off topic

(273) Lopopolo RB, Greco M, Sullivan D, Craik RL, Mangione KK. Effect of Therapeutic Exercise on Gait Speed in Community-Dwelling Elderly People: A Meta-analysis. Physical Therapy 2006 April;86(4):520-40. Review article

(274) Lord SR, Menz HB, Tiedemann A. A physiological profile approach to falls risk assessment and prevention. Physical Therapy 2003 March;83(3):237-51. Off topic

(275) Lund I, Lundeberg T, Carleson J, Sonnerfors H, Uhrlin B, Svensson E. Corticotropin releasing factor in urine--a possible biochemical marker of fibromyalgia. Responses to massage and guided relaxation. Neuroscience Letters 2006 July 31;403(1-2):166-71. Not an exercise intervention study

(276) Macario A, Lipman AG. Ketorolac in the Era of Cyclo-Oxygenase-2 Selective Nonsteroidal Anti-Inflammatory Drugs: A Systematic Review of Efficacy, Side Effects, and Regulatory Issues. Pain Med (USA) 2001 December;2(4):336-51. Drug intervention study

(277) Malaguarnera M. Carnitine derivatives: Clinical usefulness. Curr Opin Gastroenterol 2012;28(2):166-76. Review article

(278) Mancuso CA, Rincon M, Sayles W, Paget SA. Comparison of energy expenditure from lifestyle physical activities between patients with rheumatoid arthritis and healthy controls. Arthritis & Rheumatism-Arthritis Care & Research 2007;57(4):672-8. Longitudinal Study, Subjects (some or all) did not have arthritis or other disease of interest

(279) Mangione KK, Craik RL, Tomlinson SS, Palombaro KM. Can Elderly Patients Who Have Had a Hip Fracture Perform Moderate- to High-Intensity Exercise at Home? Physical Therapy 2005 August;85(8):727-39. Subjects (some or all) did not have arthritis or other disease of interest

(280) Mangione KK, Craik RL, McCormick AA, Blevins HL, White MB, Sullivan-Marx EM, Tomlinson JD. Detectable Changes in Physical Performance Measures in Elderly African Americans. Physical Therapy 2010 June;90(6):921-7. Observational study

(281) Mannerkorpi K, Arndorw M. Efficacy and feasibility of a combination of body awareness therapy and qigong in patients with fibromyalgia: a pilot study. J Rehabil Med 2004 November;36(6):279-81. Multiple interventions

(282) Mannerkorpi K, Rivano-Fischer M, Ericsson A, Nordeman L, Gard G. Experience of physical activity in patients with fibromyalgia and chronic widespread pain. Disability & Rehabilitation 2008;30(3):213-21. Survey or questionnaire

(283) Mannerkorpi K, Nordeman L, Ericsson A, Arndorw M, GAU Study Group. Pool exercise for patients with fibromyalgia or chronic widespread pain: a randomized controlled trial and subgroup analyses. Journal of Rehabilitation Medicine 2009 September;41(9):751-60.

(284) Mao JJ, Bruner DW, Stricker C, Farrar JT, Xie SX, Bowman MA, Pucci D, Han X, DeMichele A. Feasibility trial of electroacupuncture for aromatase inhibitor--related arthralgia in breast cancer survivors. Integrative Cancer Therapies 2009 June;8(2):123-9. Off topic

(285) Margolis DJ, Knauss J, Bilker W. Medical conditions associated with venous leg ulcers. British Journal of Dermatology 2004;150(2):267-73. Off topic

(286) Martin DP, Sletten CD, Williams BA, Berger IH. Improvement in fibromyalgia symptoms with acupuncture: Results of a randomized controlled trial. Mayo Clin Proc 2006;81(6):749-57. Not an exercise intervention study

(287) Martin L, Nutting A, MacIntosh BR, Edworthy SM, Butterwick D, Cook J. An exercise program in the treatment of fibromyalgia. J Rheumatol 1996 June;23(6):1050-3. Primary outcome(s) not assessed

(288) Martire LM. Couple-oriented education and support intervention: effects on individuals with osteoarthritis and their spouses. Rehabilitation Psychology 2007. Educational intervention

(289) Mayorga Buiza MJ, Fernandez Muonz. Impacto de un programa de educacion sanitaria en pacientes con fibromialgia (Impact of a health education program on patients with fibromyalgia) [Spanish]. Revista de la Sociedad Espanola del Dolor 2010. Educational intervention

(290) McBeth J, Pye SR, O'Neill TW, Macfarlane GJ, Tajar A, Bartfai G, Boonen S, Bouillon R, Casanueva F, Finn JD, Forti G, Giwercman A, Han TS, Huhtaniemi IT, Kula K, Lean ME, Pendleton N, Punab M, Silman AJ, Vanderschueren D, Wu FC, EMAS Group. Musculoskeletal pain is associated with very low levels of vitamin D in men: results from the European Male Ageing Study. Annals of the Rheumatic Diseases 2010 August;69(8):1448-52. Survey or questionnaire

(291) McCain GA, Bell DA, Mai FM, Halliday PD. A controlled study of the effects of a supervised cardiovascular fitness training program on the manifestations of primary fibromyalgia. Arthritis Rheum 1988 September;31(9):1135-41. Primary outcome(s) not assessed

(292) McGeeney BE. Pharmacological Management of Neuropathic Pain in Older Adults: An Update on Peripherally and Centrally Acting Agents. J Pain Symptom Manage 2009;38(2 SUPPL.):S15-S27. Drug intervention study

(293) McGough EL, Kelly VE, Logsdon RG, McCurry SM, Cochrane BB, Engel JM, Teri L, Liu-Ambrose TY. Associations Between Physical Performance and Executive Function in Older Adults With Mild Cognitive Impairment: Gait Speed and the Timed "Up & Go" Test. Physical Therapy 2011 August;91(8):1198-210. Cross-sectional study

(294) McNeill O, Pile K. 'My knees can tell the weather': Effective management of osteoarthritis. Med Today 2011;12(1):28-39. Review article

(295) McVeigh JG, McGaughey H, Hall M, Kane P. The effectiveness of hydrotherapy in the management of fibromyalgia syndrome: a systematic review (Structured abstract). Rheumatol Int 2008;29:119-30. Review article

(296) Mease PJ, Arnold LM, Crofford LJ, Williams DA, Russell IJ, Humphrey L, Abetz L, Martin SA. Identifying the clinical domains of fibromyalgia: contributions from clinician and patient Delphi exercises. Arthritis Rheum 2008;59(7):952-60. Not an exercise intervention study

(297) Mengshoel AM, Komnaes HB, Forre O. The effects of 20 weeks of physical fitness training in female patients with fibromyalgia. Clin Exp Rheumatol 1992 July;10(4):345-9. Primary outcome(s) not assessed

(298) Messier SP, Loeser RF, Miller GD, Morgan TM, Rejeski WJ, Sevick MA, Ettinger WH, Jr., Pahor M, Williamson JD. Exercise and dietary weight loss in overweight and obese older adults with knee osteoarthritis: the Arthritis, Diet, and Activity Promotion Trial. Arthritis Rheum 2004 May;50(5):1501-10. Primary outcome(s) not assessed

(299) Meyer BB, Lemley KJ. Utilizing exercise to affect the symptomology of fibromyalgia: A pilot study. Med Sci Sports Exerc 2000 October;32(10):1691-7. Not a randomized controlled trial (RCT)

(300) Meyer CL, Hawley DJ. Characteristics of participants in water exercise programs compared to patients seen in a rheumatic disease clinic. Arthritis Care & Research 1994 June;7(2):85-9. Not an exercise intervention study

(301) Mhalla A, Baudic S, De Andrade DC, Gautron M, Perrot S, Teixeira MJ, Attal N, Bouhassira D. Long-term maintenance of the analgesic effects of transcranial magnetic stimulation in fibromyalgia. Pain 2011;152(7):1478-85. Electrical Stimulation

(302) Miculis CP. Efeito do exercicio fisico em condicoes osteomioarticulares: revisao de literatura (Effects of the physical exercise on main joint, bone and muscle pathological conditions: a review) [Portuguese]. Fisioterapia em Movimento [Physical Therapy in Movement 2009. Review article

(303) Mikesky AE, Mazzuca SA, Brandt KD, Perkins SM, Damush T, Lane KA. Effects of strength training on the incidence and progression of knee osteoarthritis. Arthritis & Rheumatism-Arthritis Care & Research 2006;55(5):690-9. No comparative control group, Both groups exercised

(304) Mingo CA, McIlvane JM, Haley WE. Impact of the diagnostic label of osteoarthritis on perceptions of disability and willingness to help a parent in African Americans and whites. Arthritis & Rheumatism-Arthritis Care & Research 2006;55(6):913-9. Not an exercise intervention study

(305) Minor MA, Brown JD. Exercise maintenance of persons with arthritis after participation in a class experience. Health Education Quarterly 1993;20(1):83-95. Review article

(306) Morley JE. Developing novel therapeutic approaches to frailty. Curr Pharm Des 2009;15(29):3384-95. Review article

(307) Morris SL, Dodd KJ, Morris ME. Outcomes of progressive resistance strength training following stroke: a systematic review. Clin Rehabil 2004 February;18(1):27-39. Review article

(308) Moseley AM, Stark A, Cameron ID, Pollock A. Treadmill training and body weight support for walking after stroke. Cochrane Database of Systematic Reviews 2005. Review article

(309) Mueser KT, Bartels SJ, Santos M, Pratt SI, Riera EG. Integrated illness management and recovery: A program for integrating physical and psychiatric illness self-management in older persons with severe mental illness. Am J Psychiatr Rehabil 2012;15(2):131-56. Not a randomized controlled trial (RCT)

(310) Mullen PD, Laville EA, Biddle AK, Lorig. Efficacy of psychoeducational interventions on pain, depression, and disability in people with arthritis: a meta-analysis. The Journal of Rheumatology 1987. Review article

(311) Munguia-Izquierdo D, Legaz-Arrese A. Assessment of the effects of aquatic therapy on global symptomatology in patients with fibromyalgia syndrome: a randomized controlled trial. Arch Phys Med Rehabil 2008 December;89(12):2250-7. Primary outcome(s) not assessed

(312) Munguia-Izquierdo D, Legaz-Arrese A. Determinants of sleep quality in middle-aged women with fibromyalgia syndrome. Journal of Sleep Research 2012;21(1):73-9. Survey or questionnaire

(313) Nueesch E, Dieppe P, Reichenbach S, Williams S, Iff S, Juni P. All cause and disease specific mortality in patients with knee or hip osteoarthritis: Population based cohort study. BMJ 2011;342(7798):638. Cohort Study

(314) Nagtegaal JE, Laurant MW, Kerkhof GA, Smits MG, van der Meer YG, Coenen AML. Effects of melatonin on the quality of life in patients with delayed sleep phase syndrome. J Psychosom Res 2000;48(1):45-50. Diet Intervention or Supplement Study

(315) Nees F, Ruddel H, Mussgay L, Kuehl LK, Romer S, Schachinger H. Alteration of Delay and Trace Eyeblink Conditioning in Fibromyalgia Patients. Psychosom Med 2010;72(4):412-8. Not an exercise intervention study

(316) Nicassio PM. A comparison of behavioral and educational interventions for fibromyalgia. The Journal of Rheumatology 1997. Behavior Modification Intervention

(317) Nichols AW. Sports medicine clinical trial research publications in academic medical journals between 1996 and 2005: an audit of the PubMed MEDLINE database. British Journal of Sports Medicine 2008 November;42(11):609-21. Off topic

(318) Nichols DS, Glenn TM. Effects of Aerobic Exercise on Pain Perception, Affect, and Level of Disability in Individuals With Fibromyalgia. Physical Therapy 1994 April 1;74(4):327-32. Primary outcome(s) not assessed

(319) Nicolson SE, Caplan JP, Williams DE, Stern TA. Comorbid pain, depression, and anxiety: Multifaceted pathology allows for multifaceted treatment. Harv Rev Psychiatry 2009;17(6):407-20. Review article

(320) Nieman DC. Chapter 21: THE ELDERLY. Exercise-Health Connection 1998 January;289-300. Not a randomized controlled trial (RCT)

(321) Nilsson H. Resilient appliance therapy of temporomandibular disorders. Subdiagnoses, sense of coherence and treatment outcome. Swedish Dental Journal 2010. Off topic

(322) Noreau L, Martineau H, Roy L, Belzile M. Effects of a modified dance-based exercise on cardiorespiratory fitness, psychological state and health status of persons with rheumatoid arthritis. American Journal of Physical Medicine & Rehabilitation 1995 January;74(1):19-27. CT

(323) Norregaard J. Exercise training in treatment of fibromyalgia. J MUSCULOSKELET PAIN 1997. Inappropriate Comparison Group

(324) Nosse LJ, Sagiv L. Theory-Based Study of the Basic Values of 565 Physical Therapists. Physical Therapy 2005 September;85(9):834-971. Off topic

(325) Nour K, Laforest S, Gignac M, Gauvin L. Appreciating the predicament of housebound older adults with arthritis: Portrait of a population. Canadian Journal on Aging-Revue Canadienne du Vieillissement 2005;24(1):57-69. Not a randomized controlled trial (RCT)

(326) Nour K, Laforest S, Gauvin L, Gignac M. Behavior change following a self-management intervention for housebound older adults with arthritis: An experimental study. Int J Behav Nutr Phys Act 2006;3. Behavior Modification Intervention

(327) Oh H, Seo W. Decreasing pain and depression in a health promotion program for people with rheumatoid arthritis. J Nurs Scholarsh 2003;35(2):127-32. Not a randomized controlled trial (RCT)

(328) Oja P, Titze S, Bauman A, de Geus B, Krenn P, Reger-Nash B, Kohlberger T. Health benefits of cycling: a systematic review. Scandinavian Journal of Medicine & Science in Sports 2011 August;21(4):496-509. Review article

(329) Oliver K. Effects of social support and education on health care costs for patients with fibromyalgia. The Journal of Rheumatology 2001. Not an exercise intervention study

(330) Ormseth MJ, Eyler AE, Hammonds CL, Boomershine CS. Milnacipran for the management of fibromyalgia syndrome. J Pain Res 2010;3:15-24. Drug intervention study

(331) Ortega E, Garcia JJ, Bote ME, Martin-Cordero L, Escalante Y, Saavedra JM, Northoff H, Giraldo E. Exercise in fibromyalgia and related inflammatory disorders: Known effects and unknown chances. Exerc Immunol Rev 2009;15:42-65. Review article

(332) Overlack A, Adamczak M, Bachmann W, Bonner G, Bretzel RG, Derichs R, Krone W, Lederle RM, Reimann HJ, Zschiedrich H, Stumpe KO, Cyran J, Diehm C, Fuchs G, Gotzen R, Heimsoth V, Knauf H, Kronig B, Maier KE, Middeke M, Sanden HV, Sholze J. ACE-inhibition with perindopril in essential hypertensive patients with concomitant diseases. AM J MED 1994;97(2):126-34. Drug intervention study

(333) Ozgocmen S, Catal SA, Ardicoglu O, Kamanli A. Effect of omega-3 fatty acids in the management of fibromyalgia syndrome. Int J Clin Pharmacol Ther 2000;38(7):362. Diet Intervention or Supplement Study

(334) Page MJ, O'Connor D, Pitt V, Massy WN. Exercise and mobilisation interventions for carpal tunnel syndrome. Cochrane Database of Systematic Reviews 2012. Review article

(335) Park J. Managing osteoarthritis: comparisons of chair yoga, Reiki, and education (pilot study). Holistic Nursing Practice 2011. Not a randomized controlled trial (RCT)

(336) Parker JC, Singsen BH, Hewett JE, Walker SE, Hazelwood SE, Hall PJ, Holsten DJ, Rodon CM. Educating patients with rheumatoid arthritis: a prospective analysis. Arch Phys Med Rehabil 1984. Educational intervention

(337) Passard A, Attal N, Benadhira R, Brasseur L, Saba G, Sichere P, Perrot S, Januel D, Bouhassira D. Effects of unilateral repetitive transcranial magnetic stimulation of the motor cortex on chronic widespread pain in fibromyalgia. Brain 2007 October;130(Pt:10):10-70. Electrical Stimulation

(338) Patten SB, Williams JVA, Wang JL. Mental disorders in a population sample with musculoskeletal disorders. BMC Musculoskeletal Disorders 2006;7. Cross-sectional study

(339) Pearl SJ. The effects of bright light treatment on the symptoms of fibromyalgia. The Journal of Rheumatology 1996. Not an exercise intervention study

(340) Pedersen BK. Evidence for prescribing exercise as therapy in chronic disease. Scandinavian Journal of Medicine & Science in Sports 2006. Review article

(341) Persson AL. Relaxation as treatment for chronic musculoskeletal pain -- a systematic review of randomised controlled studies. Physical Therapy Reviews 2008. Review article

(342) Peters S, Stanley I, Rose M, Kaney S, Salmon P. A randomized controlled trial of group aerobic exercise in primary care patients with persistent, unexplained physical symptoms. Family practice 2002;19:665-74. Subjects (some or all) did not have arthritis or other disease of interest

(343) Possley D, Budiman-Mak E, O'Connell S, Jelinek C, Collins EG. Relationship between depression and functional measures in overweight and obese persons with osteoarthritis of the knee. J Rehabil Res Dev 2009;46(9):1091-7. Cross-sectional study

(344) Potocnik P, Acklin YP, Sommer C. Operative strategy in postero-medial fracture-dislocation of the proximal tibia. Injury 2011;42(10):1060-5. Off topic

(345) Pyhi R, Da Costa D, Fitzcharles MA. Pain and pain relief in fibromyalgia patients followed for three years. Arthritis Care Res 2001;45(4):355-61. Prospective Study

(346) Ramsay C, Moreland J, Ho M, Joyce S, Walker S, Pullar T. An observer-blinded comparison of supervised and unsupervised aerobic exercise regimens in fibromyalgia. Rheumatology 2000;39(5):501-5. No comparative control group, Both groups exercised

(347) Raspe HH, Deck R, Mattussek S. The outcome of traditional or comprehensive outpatient care for rheumatoid arthritis (RA). Results of an open, non-randomized, 2-year prospective study. Zeitschrift fur Rheumatologie 1992;51:Suppl-6. Not a randomized controlled trial (RCT)

(348) Redondo JR, Justo CM, Moraleda FV, Velayos YG, Puche JJ, Zubero JR, Hernandez TG, Ortells LC, Pareja MA. Long-term efficacy of therapy in patients with fibromyalgia: a physical exercise-based program and a cognitive-behavioral approach. Arthritis and Rheumatism 2004. No non-intervention control group

(349) Reid MC, Otis J, Barry LC, Kerns RD. CognitiveΓÇôBehavioral Therapy for Chronic Low Back Pain in Older Persons: A Preliminary Study. Pain Med (USA) 2003 September;4(3):223. Not a randomized controlled trial (RCT)

(350) Reid MC, Papaleontiou M, Ong A, Breckman R, Wethington E, Pillemer K. Self-Management Strategies to Reduce Pain and Improve Function among Older Adults in Community Settings: A Review of the Evidence. Pain Med (USA) 2008 May;9(4):409-24. Review article

(351) Rhudy JL, Dubbert PM, Kirchner KA, Williams AE. Efficacy of a program to encourage walking in VA elderly primary care patients: The role of pain. Psychology, Health & Medicine 2007 May;12(3):289-98. Subjects (some or all) did not have arthritis or other disease of interest

(352) Richards SC, Scott DL. Prescribed exercise in people with fibromyalgia: parallel group randomised controlled trial. BMJ 2002 July 27;325(7357):185. No non-intervention control group, Primary outcome(s) not assessed

(353) Riddle DL, Kong X, Fitzgerald GK. Psychological health impact on 2-year changes in pain and function in persons with knee pain: data from the Osteoarthritis Initiative. Osteoarthritis & Cartilage 2011 September;19(9):1095-101. Cohort Study

(354) Riemsma RP, Kirwan JR, Taal E, Rasker JJ. Patient education for adults with rheumatoid arthritis (Cochrane review) [with consumer summary]. Cochrane Database of Systematic Reviews 2003. Review article

(355) Riley D, Zagon A. Clinical homeopathic use of RNA: Evidence from two provings. Homeopathy 2005;94(1):33-6. Drug intervention study

(356) Robb-Nicholson LC, Daltroy L, Eaton H, Gall V, Wright E, Hartley LH, Schur PH, Liang MH. Effects of aerobic conditioning in lupus fatigue: a pilot study. British Journal of Rheumatology 1989 December;28(6):500-5. Subjects (some or all) did not have arthritis or other disease of interest

(357) Rolhstein JM. PT 2000: The Annual Conference and Exposition of the APTA Abstracts of Papers Accepted for Presentation. Physical Therapy 2000 May;80(5):S1-S71. Abstract

(358) Romero-Zurita A, Carbonell-Baeza A, Aparicio VA, Ruiz JR, Tercedor P, Delgado-Fernandez M. Effectiveness of a Tai-Chi training and detraining on functional capacity, symptomatology and psychological outcomes in women with fibromyalgia. Evid -Based Complement Altern Med 2012;2012. Not a randomized controlled trial (RCT)

(359) Rooks DS, Silverman CB, Kantrowitz FG. The effects of progressive strength training and aerobic exercise on muscle strength and cardiovascular fitness in women with fibromyalgia: a pilot study. Arthritis Rheum 2002 February;47(1):22-8. No control group (NC)

(360) Rosemann T, Kuehlein T, Laux G, Szecsenyi J. Osteoarthritis of the knee and hip: a comparison of factors associated with physical activity. Clin Rheumatol 2007;26(11):1811-7. Survey or questionnaire

(361) Rosemann T, Joos S, Laux G, Gensichen J, Szecsenyi J. Case management of arthritis patients in primary care: a cluster-randomized controlled trial. Arthritis Rheum 2007 December 15;57(8):1390-7. Educational intervention

(362) Rosemann T, Backenstrass M, Joest K, Rosemann A, Szecsenyi J, Laux G. Predictors of depression in a sample of 1,021 primary care patients with osteoarthritis. Arthritis & Rheumatism-Arthritis Care & Research 2007;57(3):415-22. Survey or questionnaire

(363) Rothstein JM. PT 2001: The Annual Conference and Exposition of the APTA Abstracts of Papers Accepted for Presentation. Physical Therapy 2001 May;81(5):A1-A86. Abstract

(364) Russell IJ, Mease PJ, Smith TR, Kajdasz DK, Wohlreich MM, Detke MJ, Walker DJ, Chappell AS, Arnold LM. Efficacy and safety of duloxetine for treatment of fibromyalgia in patients with or without major depressive disorder: Results from a 6-month, randomized, double-blind, placebo-controlled, fixed-dose trial. Pain 2008 June;136(3):432-44. Drug intervention study

(365) Rutledge DN, Jones CJ. Effects of topical essential oil on exercise volume after a 12-week exercise program for women with fibromyalgia: a pilot study. Journal of Alternative & Complementary Medicine 2007 December;13(10):1099-106. No non-intervention control group

(366) Sampson SM, Rome JD, Rummans TA. Slow-frequency rTMS reduces fibromyalgia pain. Pain Med (USA) 2006 March;7(2):115-8. Electrical Stimulation

(367) Santamato A, Solfrizzi V, Panza F, Tondi G, Frisadi V, Leggin BG, Ranieri M, Fiore P. Short-term Effects of High-Intensity Laser Therapy Versus Ultrasound Therapy in the Treatment of People With Subacromial Impingement Syndrome: A Randomized Clinical Trial. Physical Therapy 2009 July;89(7):643-52. Off topic

(368) Santana JS, Almeida AP, Brandao PM. [The effect of Ai Chi method in fibromyalgic patients]. Cien Saude Colet 2010 June;15 Suppl 1:1433-8. Primary outcome(s) not assessed

(369) Sanudo B, Galiano D, Carrasco L, Blagojevic M, de HM, Saxton J. Aerobic exercise versus combined exercise therapy in women with fibromyalgia syndrome: a randomized controlled trial. Archives of Physical Medicine & Rehabilitation 2010 December;91(12):1838-43. Same subjects as another study already included

(370) Sanudo B. Effects of exercise training and detraining in patients with fibromyalgia syndrome: a 3-yr longitudinal study. American Journal of Physical Medicine & Rehabilitation 2012. Same subjects as another study already included

(371) Sarzi-Puttini P, Atzeni F, Lanata L, Bagnasco M, Colombo M, Fischer F, D'Imporzano M. Pain and ketoprofen: What is its role in clinical practice? Reumatismo 2010;62(3):172-88. Drug intervention study

(372) Savage R. Cyclo-oxygenase-2 inhibitors: When should they be used in the elderly? Drugs Aging 2005;22(3):185-200. Drug intervention study

(373) Schaafsma F, Schonstein E, Whelan KM, Ulvestad E, Kenny DT, Verbeek JH. Physical conditioning programs for improving work outcomes in workers with back pain. Cochrane Database of Systematic Reviews 2010. Subjects (some or all) did not have arthritis or other disease of interest

(374) Schilling ML. Pain management in older adults. Curr Psychiatry Rep 2003;5(1):55-61. Review article

(375) Schmidt S, Grossman P, Schwarzer B, Jena S, Naumann J, Walach H. Treating fibromyalgia with mindfulness-based stress reduction: results from a 3-armed randomized controlled trial. Pain 2011 February;152(2):361-9. Not an exercise intervention study

(376) Scholten C, Brodowicz T, Graninger W, Gardavsky I, Pils K, Pesau B, Eggl-Tyl E, Wanivenhaus A, Zielinski CC. Persistent functional and social benefit 5 years after a multidisciplinary arthritis training program. Archives of Physical Medicine & Rehabilitation 1999 October;80(10):1282-7. Study less than 4 weeks

(377) Scholz BA, Hammonds CL, Boomershine CS. Duloxetine for the management of fibromyalgia syndrome. J Pain Res 2009;2:99-108. Drug intervention study

(378) Schulte PA, Wagner GR, Blanciforti LA, Cutlip RG, Krajnak KM, Luster M, Munson AE, O'Callaghan JP, Parks CG, Simeonova PP, Miller DB, Ostry A. Work, Obesity, and Occupational Safety and Health. American Journal of Public Health 2007 March;97(3):428-36. Off topic

(379) Scopaz KA, Piva SR, Wisniewski S, Fitzgerald GK. Relationships of Fear, Anxiety, and Depression With Physical Function in Patients With Knee Osteoarthritis. Arch Phys Med Rehabil 2009;90(11):1866-73. Cross-sectional study

(380) Scudds RA, Janzen V, Delaney G, Heck C, McCain GA, Russell AL, Teasell RW, Varkey G, Gail Woodbury M. The use of topical 4% lidocaine in spheno-palatine ganglion blocks for the treatment of chronic muscle pain syndromes: A randomized, controlled trial. Pain 1995;62(1):69-77. Drug intervention study

(381) Sebro B. Sponatano koristenje aktivnih pasivnih strategija za borbu s bolovima u bolesnika s reumatoidnim artritisom (Spontaneous use of active and passive coping strategies for pain in patients with rheumatoid arthritis) [Croatian]. Reumatizam 1993. Not an exercise intervention study

(382) Seltzer EG, Gerber MA, Cartter ML, Freudigman K, Shapiro ED. Long-term outcomes of persons with Lyme disease. Jama-Journal of the American Medical Association 2000;283(5):609-16. Subjects (some or all) did not have arthritis or other disease of interest

(383) Sephton SE. Mindfulness meditation alleviates depressive symptoms in women with fibromyalgia: results of a randomized clinical trial. Arthritis and Rheumatism 2007. Not an exercise intervention study

(384) Sharpe L. A blind, randomized, controlled trial of cognitive-behavioural intervention for patients with recent onset rheumatoid arthritis: preventing psychological and physical morbidity. Pain 2001. Behavior Modification Intervention

(385) Sharpe L. Long-term efficacy of a cognitive behavioural treatment from a randomized controlled trial for patients recently diagnosed with rheumatoid arthritis. Rheumatology 2003. Behavior Modification Intervention

(386) Shearn MA. Stress management and mutual support groups in rheumatoid arthritis. The American Journal of Medicine 1985. Not an exercise intervention study

(387) Shephard RJ. Physical activity, fitness, and health: the current consensus. Quest (00336297) 1995 August;47(3):288-303. Review article

(388) Sherman AM, Shumaker SA, Rejeski. Social support, social integration, and health-related quality of life over time: results from the Fitness and Arthritis in Seniors Trial (FAST). Psychology & Health 2006. Review article

(389) Skelton DA, Beyer N. Exercise and injury prevention in older people. Scandinavian Journal of Medicine & Science in Sports 2003 February;13(1):77-85. Review article

(390) Skljarevski V, Desaiah D, Liu-Seifert H, Zhang Q, Chappell AS, Detke MJ, Iyengar S, Atkinson JH, Backonja M. Efficacy and Safety of Duloxetine in Patients With Chronic Low Back Pain. Spine 2010;35(13):E578-E585. Off topic

(391) Slatkovska L, Alibhai SMH, Beyene J, Hu H, Demaras A, Cheung AM. Effect of 12 months of whole-body vibration therapy on bone density and structure in postmenopausal women:A randomized trial. Ann Intern Med 2011;155(10):668-79. Off topic

(392) Smeets RJEM, Beelen S, Goossens MEJB, Schouten EGW, Knottnerus JA, Vlaeyen JWS. Treatment expectancy and credibility are associated with the outcome of both physical and cognitive-behavioral treatment in chronic low back pain. Clin J Pain 2008;24(4):305-15. Subjects (some or all) did not have arthritis or other disease of interest

(393) Staud R, Robinson ME, Price DD. Isometric exercise has opposite effects on central pain mechanisms in fibromyalgia patients compared to normal controls. Pain 2005 November;118(1-2):176-84. Subjects (some or all) did not have arthritis or other disease of interest

(394) Stephens S, Feldman BM, Bradley N, Schneiderman J, Wright V, Singh-Grewal D, Lefebvre A, Benseler SM, Cameron B, Laxer R, O'Brien C, Schneider R, Silverman E, Spiegel L, Stinson J, Tyrrell PN, Whitney K, Tse SM. Feasibility and effectiveness of an aerobic exercise program in children with fibromyalgia: results of a randomized controlled pilot trial. Arthritis Rheum 2008 October 15;59(10):1399-406. Study limited to children and/or adolescents

(395) Stern M, Sorkin L, Milton K, Sperber K. Aging with multiple sclerosis. Phys Med Rehabil Clin North Am 2010;21(2):403-17. Review article

(396) Strine TW, Hootman JM, Okoro CA, Balluz L, Moriarty DG, Owens M, Mokdad A. Frequent mental distress status among adults with arthritis age 45 years and older, 2001. Arthritis & Rheumatism-Arthritis Care & Research 2004;51(4):533-7. Survey or questionnaire

(397) Strine TW, Hootman JM, Chapman DP, Okoro CA, Balluz L. Health-Related Quality of Life, Health Risk Behaviors, and Disability Among Adults With Pain-Related Activity Difficulty. American Journal of Public Health 2005 November;95(11):2042-8. Cross-sectional study

(398) Sullivan M, Bentley S, Fan MY, Gardner G. A single-blind placebo run-in study of venlafaxine XR for activity-limiting osteoarthritis pain. Pain Med (USA) 2009;10(5):806-12. Drug intervention study

(399) Sullivan MD, Bentley S, Fan MY, Gardner G. A Single-Blind, Placebo Run-in Study of Duloxetine for Activity-Limiting Osteoarthritis Pain. J Pain 2009;10(2):208-13. Drug intervention study

(400) Sullivan SS, Guilleminault C. Emerging drugs for insomnia: New frontiers for old and novel targets. Expert Opin Emerg Drugs 2009;14(3):411-22. Drug intervention study

(401) Sultan A, Gaskell H, Derry S, Moore RA. Duloxetine for painful diabetic neuropathy and fibromyalgia pain: systematic review of randomised trials. Bmc Neurology 2008;8. Drug intervention study

(402) Suman AL, Biagi B, Biasi G, Carli G, Gradi M, Prati E, Bonifazi M. One-year efficacy of a 3-week intensive multidisciplinary non-pharmacological treatment program for fibromyalgia patients. Clinical & Experimental Rheumatology 2009 January;27(1):7-14. Study less than 4 weeks

(403) Sunshine W. Fibromyalgia benefits frommassage therapy and transcutaneous electrical stimulation. Journal of Clinical Rheumatology 1996. Not an exercise intervention study

(404) Sutbeyaz ST. Low-frequency pulsed electromagnetic field therapy in fibromyalgia: a randomized, double-blind, sham-controlled clinical study. The Clinical Journal of Pain 2009. Electrical Stimulation

(405) Sverdrup B. Use less cosmetics - Suffer less from fibromyalgia? J Women's Health 2004;13(2):187-94. Off topic

(406) Szoeke CEI, Dennerstein L, Wluka AE, Guthrie JR, Taffe J, Clark MS, Cicuttini FM. Physician diagnosed arthritis, reported arthritis and radiological non-axial osteoarthritis. Osteoarthritis Cartilage 2008;16(7):846-50. Longitudinal Study

(407) Takiguchi RS, Fukuhara VS, Sauer JF, Assumpcao. Efeito da acupuntura na melhora da dor, sono e qualidade de vida em pacientes fibromialgicos: estudo preliminar (Effect of acupuncture on pain, sleep and quality of life improvement in fibromyalgia patients: preliminary study) [Portuguese]. Fisioterapia e Pesquisa [Physical Therapy and Research] 2008. Inappropriate Intervention

(408) Targino RA. A randomized controlled trial of acupuncture added to usual treatment for fibromyalgia. Journal of Rehabilitation Medicine 2008. No non-intervention control group

(409) Taulbee C. The association between fibromyalgia symptoms and irritable bowel syndrome subtypes. United States -- California: California State University, Fullerton; 2011. Off topic

(410) Taylor-Piliae RE, Newell KA, Cherin R, Lee MJ, King AC, Haskell WL. Effects of Tai Chi and Western Exercise on Physical and Cognitive Functioning in Healthy Community-Dwelling Older Adults. Journal of Aging & Physical Activity 2010 July;18(3):261-79. Primary outcome(s) not assessed

(411) Taylor-Piliae RE, Coull BM. Community-based Yang-style Tai Chi is safe and feasible in chronic stroke: a pilot study. Clin Rehabil 2012 February;26(2):121-31. Subjects (some or all) did not have arthritis or other disease of interest

(412) Taylor AH, Cable NT, Faulkner G, Hillsdon M, Narici M, van Der Bij AK. Physical activity and older adults: a review of health benefits and the effectiveness of interventions. Journal of Sports Sciences 2004 August;22(8):703-25. Review article

(413) Tektonidou MG, Dasgupta A, Ward MM. Suicidal Ideation Among Adults With Arthritis: Prevalence and Subgroups at Highest Risk. Data From the 2007-2008 National Health and Nutrition Examination Survey. Arthritis Care & Research 2011;63(9):1322-33. Cross-sectional study

(414) The GKH, Prins J, Bleijenberg G, van der Meer JWM. The effect of granisetron, a 5-HT3 receptor antagonist, in the treatment of chronic fatigue syndrome patients - a pilot study. Netherlands Journal of Medicine 2003;61(9):285-9. Drug intervention study

(415) Theis KA, Helmick CG, Hootman JM. Arthritis burden and impact are greater among US women than men: Intervention opportunities. Journal of Womens Health 2007;16(4):441-53. Review article

(416) Thomas KS, Muir KR, Doherty. Home based exercise programme for knee pain and knee osteoarthritis: randomised controlled trial [with consumer summary]. BMJ 2002. Subjects (some or all) did not have arthritis or other disease of interest

(417) Thomas EN. Aerobic exercise in fibromyalgia: a practical review. Rheumatology Internationa 2010. Review article

(418) Toda Y, Kobayashi T. The usefulness of walking for preventing sarcopenia in dieting postmenopausal women complaining of knee pain. Annals of the New York Academy of Sciences 2000;904:610-3. Primary outcome(s) not assessed

(419) Tomas-Carus P, Hakkinen A, Gusi N, Leal A, Hakkinen K, Ortega-Alonso A. Aquatic training and detraining on fitness and quality of life in fibromyalgia. Med Sci Sports Exerc 2007 July;39(7):1044-50. Primary outcome(s) not assessed

(420) Trojan DA, Arnold D, Collet JP, Shapiro S, Bar-Or A, Robinson A, Le Cruguel JP, Ducruet T, Narayanan S, Arcelin K, Wong AN, Tartaglia MC, Lapierre Y, Caramanos Z, Costa D. Fatigue in multiple sclerosis: association with disease-related, behavioural and psychosocial factors. Multiple Sclerosis 2007;13(8):985-95. Subjects (some or all) did not have arthritis or other disease of interest

(421) Turk DC, Vierck CJ, Scarbrough E, Crofford LJ, Rudin NJ. Fibromyalgia: Combining Pharmacological and Nonpharmacological Approaches to Treating the Person, Not Just the Pain. J Pain 2008;9(2):99-104. Review article

(422) Turner JA, Ersek M, Kemp C. Self-efficacy for managing pain is associated with disability, depression, and pain coping among retirement community residents with chronic pain. J Pain 2005;6(7):471-9. Subjects (some or all) did not have arthritis or other disease of interest

(423) Ulus Y, Tander B, Akyol Y, Durmus D, Buyukakincak O, Gul U, Canturk F, Bilgici A, Kuru O. Therapeutic ultrasound versus sham ultrasound for the management of patients with knee osteoarthritis: a randomized double-blind controlled clinical study. International Journal of Rheumatic Diseases 2012 April;15(2):197-206. Electrical Stimulation

(424) Unutzer J, Hantke M, Powers D, Higa L, Lin E, Vannoy D, Thielke S, Fan MY. Care management for depression and osteoarthritis pain in older primary care patients: a pilot study. Int J Geriatr Psychiatry 2008 November;23(11):1166-71. Not a randomized controlled trial (RCT)

(425) Valeikiene V, Ceremnych J, Alekna V, Jumulynasam A. Differences in WHOQOL-100 domain scores in Parkinson's disease and osteoarthritis. Medical Science Monitor 2008;14(4):CR221-CR227. Survey or questionnaire

(426) Valkeinen H, Alen M, Hakkinen A, Hannonen P, Kukkonen-Harjula K, Hakkinen K. Effects of Concurrent Strength and Endurance Training on Physical Fitness and Symptoms in Postmenopausal Women With Fibromyalgia: A Randomized Controlled Trial. Arch Phys Med Rehabil 2008 September;89(9):1660-6. Primary outcome(s) not assessed

(427) Van Abbema R, Van Wilgen CP, Van der Schans CP, Van Ittersum MW. Patients with more severe symptoms benefit the most from an intensive multimodal programme in patients with fibromyalgia. Disability and Rehabilitation 2011;33(9):743-50. Not a randomized controlled trial (RCT)

(428) van den Ende CH, Breedveld FC, Dijkmans BA, Hazes JM. The limited value of the Health Assessment Questionnaire as an outcome measure in short term exercise trials. J Rheumatol 1997 October;24(10):1972-7. Review article

(429) Van Koulil S, Kraaimaat FW, Van Lankveld W, Van Helmond T, Vedder A, Van Hoorn H, Cats H, Van Riel PLCM, Evers AWM. Screening for pain-persistence and pain-avoidance patterns in fibromyalgia. Int J Behav Med 2008;15(3):211-20. Not an exercise intervention study

(430) van Santen M, Bolwijn P, Landewe R, Verstappen F, Bakker C, Hidding A, van der Heijde D, Houben H, van der Linden S. High or low intensity aerobic fitness training in fibromyalgia: Does it matter? J Rheumatol 2002;29(3):582-7. No comparative control group, Both groups exercised

(431) van Santen M, Bolwijn P, Verstappen F, Bakker C, Hidding A, Houben H, van der Heijde D, Landewe R, van der Linden S. A randomized clinical trial comparing fitness and biofeedback training versus basic treatment in patients with fibromyalgia. The Journal of Rheumatology 2002 March 1;29(3):575-81. Primary outcome(s) not assessed

(432) Van DJ, Harlowe D. The efficacy of the ROM Dance Program for adults with rheumatoid arthritis. Am J Occup Ther 1987 February;41(2):90-5. Multiple interventions

(433) van SM, Bolwijn P, Verstappen F, Bakker C, Hidding A, Houben H, van der Heijde D, Landewe R, van der Linden S. A randomized clinical trial comparing fitness and biofeedback training versus basic treatment in patients with fibromyalgia. J Rheumatol 2002 March;29(3):575-81. Educational intervention

(434) Verstappen FTJ, van Santen-Houeft HMS, Bolwin PH, van der Linden S, Kuipers H. Effects of a Group Activity Program for Fibromyalgia Patients on Physical Fitness and Well Being. J MUSCULOSKELET PAIN 1997 January 1;5(4):17-28. Primary outcome(s) not assessed

(435) Verweij LM, Van Schoor NM, Deeg DJH, Dekker J, Visser M. Physical activity and incident clinical knee osteoarthritis in older adults. Arthritis Care Res 2009;61(2):152-7. Longitudinal Study

(436) Victor CR, Triggs E, Ross F, Lord J, Axford JS. Lack of benefit of a primary care-based nurse-led education programme for people with osteoarthritis of the knee. Clin Rheumatol 2005;24(4):358-64. Educational intervention

(437) Vincent KR, Vincent HK. Resistance Exercise for Knee Osteoarthritis. Pm&R 2012;4(5):S45-S52. Review article

(438) Vliet Vlieland TPM, Zwinderman AH, Breedveld FC, Hazes JMW. Goede resultaten van een korte klinische behandeling door een multidisciplinair team bij patienten met reumatoide arthritis; een gerandomiseerd onderzoek (Favourable effect of a short period of inpatient multidisciplinary team core in rheumatoid arthritis: a randomised trial) [Dutch]. Nederlands Tijdschrift voor Geneeskunde 1997. Study less than 4 weeks

(439) von KM. Group interventions for co-morbid insomnia and osteoarthritis pain in primary care: the lifestyles cluster randomized trial design. Contemp Clin Trials 2012. Behavior Modification Intervention

(440) Wagenmakers R, Stevens M, Groothoff JW, Zijlstra W, Bulstra SK, Van Beveren J, Van Raaij JJAM, Akker-Scheek Ivd. Physical Activity Behavior of Patients 1 Year After Primary Total Hip Arthroplasty: A Prospective Multicenter Cohort Study. Physical Therapy 2011 March;91(3):373-80. Cohort Study

(441) Walteros C, Sanchez-Navarro JP, Munoz MA, Martinez-Selva JM, Chialvo D, Montoya P. Altered associative learning and emotional decision making in fibromyalgia. J Psychosom Res 2011;70(3):294-301. Survey or questionnaire

(442) Wang C, Collett JP, Lau J. The effect of tai chi on health outcomes in patients with chronic conditions: a systematic review (Structured abstract). Archives of Internal Medicine 2004;164:493-501. Review article

(443) Wang C, Roubenoff R, Lau J, Kalish R, Schmid CH, Tighiouart H, Rones R, Hibberd PL. Effect of Tai Chi in adults with rheumatoid arthritis. Rheumatology (Oxford) 2005 May;44(5):685-7. Both groups exercised

(444) Wang C. Tai Chi improves pain and functional status in adults with rheumatoid arthritis: results of a pilot single-blinded randomized controlled trial. Medicine & Sport Science 2008;52:218-29. Both groups exercised

(445) Wang CC, Schmid CH, Hibberd PL, Kalish R, Roubenoff R, Rones R, Okparavero A, McAlindon T. Tai Chi for treating knee osteoarthritis: Designing a long-term follow up randomized controlled trial. BMC Musculoskeletal Disorders 2008;9. Description versus conduct of study

(446) Wang CC. Tai Chi and Rheumatic Diseases. Rheumatic Disease Clinics of North America 2011;37(1):19-32. Review article

(447) Wang WC, Zhang AL, Rasmussen B, Lin LW, Dunning T, Kang SW, Park BJ, Lo SK. The effect of Tai Chi on psychosocial well-being: a systematic review of randomized controlled trials (Structured abstract). Journal of Acupuncture and Meridian Studies 2009;2:171-81. Review article

(448) Warburton DER, Katzmarzyk PT, Rhodes RE, Shephard RJ. Evidence-informed physical activity guidelines for Canadian adults. Applied Physiology, Nutrition & Metabolism 2007 December 3;32:S16-S68. Review article

(449) Warburton DER, Gledhill N, Jamnik VK, Bredin SSD, McKenzie DC, Stone J, Charlesworth S, Shephard RJ. Evidence-based risk assessment and recommendations for physical activity clearance: Consensus Document 2011<sup>1. Applied Physiology, Nutrition & Metabolism 2011 July 2;36(S1):S266-S298. Review article

(450) Warren JW, Howard FM, Cross RK, Good JL, Weissman MM, Wesselmann U, Langenberg P, Greenberg P, Clauw DJ. Antecedent Nonbladder Syndromes in Case-Control Study,of Interstitial Cystitis/Painful Bladder Syndrome. Urology 2009;73(1):52-7. Off topic

(451) Wasielewski NJ, Parker TM, KKotsko KM. Evaluation of Electromyographic Biofeedback for the Quadriceps Femoris: A Systematic Review. Journal of Athletic Training 2011 September;46(5):543-54. Review article

(452) Wei CY. Effects of topical 024(TM) essential oils during a 12-week exercise program on functional health and well-being of women with fibromyalgia syndrome. United States -- California: California State University, Fullerton; 2006. Diet Intervention or Supplement Study No non-intervention control group

(453) Weiner D, Pieper C, McConnell E, Martinez S, Keefe F. Pain measurement in elders with chronic low back pain: Traditional and alternative approaches. Pain 1996;67(2-3):461-7. Cross-sectional study

(454) Westcott WL. Strength training for frail older adults. Journal on Active Aging 2009 July;8(4):52-9. Review article

(455) Wigers SH. Fibromyalgia outcome: the predictive values of symptom duration, physical activity, disability pension, and critical life events--a 4.5 year prospective study. J Psychosom Res 1996 September;41(3):235-43. Prospective Study

(456) Wijeratne C, Brodaty H, Hickie I. The neglect of somatoform disorders by old age psychiatry: Some explanations and suggestions for future research. Int J Geriatr Psychiatry 2003;18(9):812-9. Review article

(457) Williams DA, Kuper D, Segar M, Mohan N, Sheth M, Clauw DJ. Internet-enhanced management of fibromyalgia: A randomized controlled trial. Pain 2010 December;151(3):694-702. Behavior Modification Intervention

(458) Wohlreich MM, Sullivan MD, Mallinckrodt CH, Chappell AS, Oakes TM, Watkin JG, Raskin J. Duloxetine for the Treatment of Recurrent Major Depressive Disorder in Elderly Patients: Treatment Outcomes in Patients With Comorbid Arthritis. Psychosomatics 2009;50(4):402-12. Drug intervention study

(459) Wolf S, Foley S, Budiman-Mak E, Moritz T, O'Connell S, Jelinek C, Collins EG. Predictors of weight loss in overweight veterans with knee osteoarthritis who participated in a clinical trial. Journal of Rehabilitation Research & Development 2010;47(3):171-81. Review article

(460) Wolfe F, Michaud K. Fatigue, rheumatoid arthritis, and anti-tumor necrosis factor therapy: an investigation in 24,831 patients. J Rheumatol 2004 November;31(11):2115-20. Not an exercise intervention study

(461) Yamaoka K, Mitsunobu F, Hanamoto K, Mori S, Tanizaki Y, Sugita K. Study on biologic effects of radon and thermal therapy on osteoarthritis. J Pain 2004 February;5(1):20-5. Not an exercise intervention study

(462) Yavuzer G, Kucukdeveci A, Arasil T, Elhan A. Moclobemid treatment in primary fibromyalgia syndrome. Eur J Phys Med Rehabil 1998;8(2):35-8. Drug intervention study

(463) Yeh GY, Roberts DH, Wayne PM, Davis RB, Quilty MT, Phillips RS. Tai Chi Exercise for Patients With Chronic Obstructive Pulmonary Disease: A Pilot Study. Respiratory Care 2010;55(11):1475-82. Subjects (some or all) did not have arthritis or other disease of interest

(464) Yiasemides R, Halaki M, Cathers I, Ginn KA. Does Passive Mobilization of Shoulder Region Joints Provide Additional Benefit Over Advice and Exercise Alone for People Who Have Shoulder Pain and Minimal Movement Restriction? A Randomized Controlled Trial. Physical Therapy 2011 February;91(2):178-89. Off topic

(465) Yohannes AM, Caton S. Management of depression in older people with osteoarthritis: A systematic review. Aging & Mental Health 2010 August;14(6):637-51. Review article

(466) Yu-Kai C, Yu-Hsiang N, Chia-Liang T, Etnier JL. Physical Activity and Cognition in Older Adults: The Potential of Tai Chi Chuan. Journal of Aging & Physical Activity 2010 October;18(4):451-72. Review article

(467) Zachrisson O, Regland B, Jahreskog M, Jonsson M, Kron M, Gottfries CG. Treatment with staphylococcus toxoid in fibromyalgia/chronic fatigue syndrome - A randomised controlled trial. Eur J Pain 2002;6(6):455-66. Drug intervention study

(468) Zanni GR. Diagnosing and treating fibromyalgia. Consult Pharm 2009;24(8):572-89. Review article

(469) Zijlstra TR, van de Laar MA, Bernelot Moens HJ, Taal E, Zakraoui L, Rasker JJ. Spa treatment for primary fibromyalgia syndrome: a combination of thalassotherapy, exercise and patient education improves symptoms and quality of life. Rheumatology 2005 April;44(4):539-46. Multiple interventions, Study less than 4 weeks

(470) Zijlstra TR, Taal E, van de Laar MA, Rasker JJ. Validation of a Dutch translation of the fibromyalgia impact questionnaire. Rheumatology 2007 January;46(1):131-4. Not an exercise intervention study

(471) Zyrianova Y, Kelly BD, Sheehan J, McCarthy C, Dinan TG. The psychological impact of arthritis: the effects of illness perception and coping. Irish Journal of Medical Science 2011;180(1):203-10. Not an exercise intervention study
