# Supplementary material for: Effects of exercise on depression in adults with arthritis: a systematic review with meta-analysis of randomized controlled trials
Source: Arthritis Res Ther. 2015 Feb 3;17(1):21. doi: 10.1186/s13075-015-0533-5 (PMC4467075; doi:10.1186/s13075-015-0533-5)
Supplement: Additional file 3: — Cochrane risk of bias results for each item from each included study. This file provides an item analysis, by study, of the risk for bias using the Cochrane Risk of Bias Assessment Instrument. [file 13075_2015_533_MOESM3_ESM.docx]

Additional File 3. Cochrane risk of bias results for each item from each included study.

| **Study** | **Randomization** | **Allocation Concealment** | **Blinding (Participants & Personnel)** | **Blinding (Outcome Assessors)** | **Incomplete Data** | **Selective Reporting** | **Physically Inactive** |
| --- | --- | --- | --- | --- | --- | --- | --- |
| Alentorn-Geli et al. (2008) [6] | Low | Unclear | High | Low | Low | Unclear | Unclear |
| Buckelew et al. (1998) [7] | Low | Unclear | High | Unclear | Low | Unclear | Low |
| Daltroy et al. (1995) [8] | Low | Unclear | High | Unclear | Unclear | Unclear | High |
| Etnier et al., 2009 [9] | Low | Unclear | High | Unclear | Unclear | Unclear | Low |
| Fontaine et al., 2010 [10] | Low | Unclear | High | Unclear | Low | Unclear | Low |
| Fransen et al., 2007 [11] | Low | Unclear | High | Low | Low | Low | Low |
| Gowans et al., 2001 [12] | Low | Unclear | High | Low | Low | Unclear | Unclear |
| Haak & Scott, 2008 [13] | Low | Unclear | High | Unclear | Low | Unclear | Unclear |
| Hakkinen et al., 2001 [14] | Low | Unclear | High | Unclear | Unclear | Unclear | Unclear |
| Ide et al., 2008 [15] | Low | Low | High | Low | Low | Unclear | Unclear |
| Jones et al., 2008 [16] | Low | Unclear | High | Unclear | Low | Unclear | Low |
| Komatireddy et al., 1997 [17] | Low | Unclear | High | Low | Low | Unclear | Unclear |
| Mannerkorpi et al., 2000 [18] | Low | Unclear | High | Unclear | Low | Unclear | Unclear |
| Minor et al., 1989 [19] | Low | Unclear | High | Unclear | Unclear | Unclear | Low |
| Neuberger et al., 2007 [20] | Low | Unclear | High | Low | High | Unclear | Low |
| O’Reilly et al., 1999 [21] | Low | Low | High | High | Unclear | Unclear | Unclear |
| Patrick et al., 2001 [22] | Low | Unclear | High | Unclear | Low | Unclear | Low |
| Penninx et al., 2002 [23] | Low | Unclear | High | Unclear | Low | Unclear | Low |
| Rooks et al., 2007 [24] | Low | Low | High | Low | Unclear | Low | Unclear |
| Sanudo et al., 2011 [25] | Low | Low | High | Low | Low | Unclear | Unclear |
| Schachter et al., 2003 [26] | High | High | High | Unclear | Low | Unclear | Low |
| Sencan et al. (2004) [27] | Low | Unclear | High | Unclear | Unclear | Unclear | Unclear |
| Tomas-Carus et al. (2007) [28] | Low | Unclear | High | Unclear | Unclear | Unclear | Low |
| Tomas-Carus et al. (2008) [29] | Low | Low | High | Low | Low | Unclear | Low |
| Valim et al. (2003) [30] | Low | Unclear | High | Low | High | Unclear | Low |
| Valkeinen et al. (2004) [31] | Low | Unclear | High | Unclear | Low | Unclear | Unclear |
| Wang et al. (2009) [32] | Low | Low | High | Unclear | Low | Low | Unclear |
| Wang et al. (2010) [33] | Low | Low | High | Unclear | Low | Low | Unclear |
| Wigers et al. (1996) [34] | Low | Unclear | High | Unclear | Low | Unclear | Unclear |

Notes: Low, low risk; High, high risk; Unclear, unclear risk.
