# Supplementary material for: Effects of exercise on depression in adults with arthritis: a systematic review with meta-analysis of randomized controlled trials
Source: Arthritis Res Ther. 2015 Feb 3;17(1):21. doi: 10.1186/s13075-015-0533-5 (PMC4467075; doi:10.1186/s13075-015-0533-5)
Supplement: Additional file 4: — Table of categorical analyses results for changes in depressive symptoms. This file provides a table of all moderator analyses conducted. [file 13075_2015_533_MOESM4_ESM.docx]

Additional File 4. Table of categorical analyses results for changes in depressive symptoms.

| Variable | ES (#) | Participants (#) | $\bar{X}$ $\bar{\times}$(95% CI) |  |
| --- | --- | --- | --- | --- |
| *Study Characteristics*  Country  - US  - Other | 17  18 | 1463  986 | **-0.23 (-0.43, -0.04)***  **-0.69 (-0.87, -0.39)*** |  |
| Type of Control  - Non-intervention  - Attention control  - Usual care  - Wait-list control  - Other | 9  6  3  5  12 | 572  356  116  438  967 | **-0.44 (-0.80, -0.07)***  -0.25 (-0.80, 0.31)  **-0.48 (-0.95, -0.01)***  **-0.42 (-0.78, -0.07)***  **-0.50 (-0.74, -0.25)*** |  |
| IPD provided  - Yes  - No | 10  25 | 640  1809 | **-0.31 (-0.61, -0.01)***  **-0.47 (-0.65, -0.28)*** |  |
| Sequence generation  - Low  - High  - Unclear | 33  2  -- | 2306  143  -- | **-0.44 (-0.61, -0.27)***  -0.25 (-0.58, 0.09)  -- |  |
| Allocation concealment  - Low  - High  - Unclear | 8  2  25 | 490  143  1816 | **-0.42 (-0.60, -0.24)***  -0.25 (-0.58, 0.09)  **-0.45 (-0.66, -0.24)*** |  |
| Blinding (Participants/Personnel)  - Low  - High  - Unclear | --  35  -- | --  2449  -- | --  **-0.42 (-0.58, -0.26)***  -- |  |
| Blinding (Outcome Assessors)  - Low  - High  - Unclear | 13  1  21 | 733  180  1536 | **-0.39 (-0.70, -0.09)***  -**0.33 (-0.63, -0.03)***  **-0.45 (-0.66, -0.26)*** |  |
| Incomplete outcome data  - Low  - High  - Unclear | 22  3  10 | 1650  269  530 | **-0.33 (-0.50, -0.17)***  -0.41 (-1.46, 0.63)  **-0.62 (-0.87, -0.37)*** |  |
| Selective outcome reporting  - Low  - High  - Unclear | 6  --  29 | 355  --  2094 | **-0.36 (-0.60, -0.12)***  --  **-0.44 (-0.62, -0.25)*** |  |
| Previously Inactive  - Low  - High  - Unclear | 18  1  16 | 1597  46  806 | **-0.27 (-0.49, -0.06)***  -0.42 (-1.01, 0.17)  **-0.61 (-0.81, -0.41)*** |  |
| Type of analysis  - Per protocol  - Intention-to-treat | 27  12 | 1603  996 | **-0.45 (-0.66, -0.23)***  **-0.37 (-0.54, -0.20)*** |  |
| Sample size estimate  - Yes  - No | 18  17 | 1431  1018 | **-0.28 (-0.51, -0.05)***  -**0.59 (-0.79, -0.39)*** |  |
| Funding for study  - Yes  - No | 29  6 | 2220  229 | **-0.35 (-0.51, -0.19)***  **-0.82 (-1.21, -0.43)*** |  |
| Method to Assess Depression  - BDI  - CES-D  - FIQ  - AIMS  - MHI  - POMS  - DASS  - VAS  - HAD  *Participant Characteristics*  Adverse events  - Yes  - No  Sex  - Females  - Males  - Mixed | 9  11  10  4  1  2  2  1  1  3  7  16  --  19 | 390  1159  493  189  50  209  152  40  180  189  424  677  --  1772 | **-1.01 (-1.34, -0.69)***  **-0.26 (-0.44, -0.07)***  -0.18 (-0.48, 0.11)  **-0.51 (-0.78, -0.24)***  **-1.24 (-1.71, -0.76)***  0.35 (0.11, 0.58)  -0.14 (-0.44, 0.16)  -0.08 (-0.54, 0.38)  **-0.33 (-0.63, -0.03)***  **-0.42 (-0.74, -0.09)***  **-0.31 (-0.49, -0.13)***  **-0.71 (-0.97, -0.45)***  --  **-0.23 (-0.41, -0.05)*** |  |
| Race/Ethnicity  - Non-Hispanic White  - Other Hispanic  - Multiple  Smoking  - Yes  - No  - Some | 1  2  17  --  --  3 | 24  64  1533  --  --  225 | -0.79 (-1.62, 0.05)  -**0.55 (-1.05, -0.05)***  **-0.19 (-0.36, -0.01)***  --  --  -0.29 (-0.58, 0.002) |  |
| Overweight (BMI > 25 kg/m^2^)  - Yes  - No  - Some | 1  --  15 | --  --  -- | 0.44 (-1.05, 0.17)  --  **-0.42 (-0.69, -0.15)*** |  |
| Type of AORC  - OA  - RA  - FM  - OA & RA  - RA & SLE | 7  3  22  2  1 | 1025  245  1037  96  46 | **-0.26 (-0.38, -0.14)***  0.14 (-0.17, 0.45)  **-0.58 (-0.84, -0.31)***  **-0.53 (-0.91, -0.14)***  -0.42 (-1.01, 0.17) |  |
| Medications for AORC  - Yes  - No  - Some | 4  --  21 | 185  --  1433 | **-0.51 (-0.98, -0.03)***  --  **-0.51 (-0.73, -0.29)*** |  |
| *Intervention Characteristics*  Type of exercise - 1  - Aerobic  - Strength  - Aerobic + strength | 15  5  15 | 1017  551  1052 | **-0.53 (-0.76, -0.29)***  **-0.42 (-0.74, -0.10)***  **-0.31 (-0.58, -0.05)*** |  |
| Type of exercise - 2  - Aerobic  - Strength  - Aerobic + strength  - Tai chi or qi gong | 15  5  11  4 | 1017  551  834  259 | **-0.53 (-0.76, -0.29)***  **-0.42 (-0.74, -0.10)***  -0.19 (-0.49, 0.10)  **-0.63 (-1.16, -0.10)*** |  |
| Exercise intensity  - Low  - Moderate  - High | 3  11  5 | 139  585  598 | -0.16 (-1.39, 1.08)  **-0.38 (-0.67, -0.08)***  **-0.25 (-0.40, -0.11)*** |  |
| Exercise delivery  - Supervised  - Unsupervised  - Supervised + Unsupervised | 18  7  10 | 990  621  906 | **-0.58 (-0.89, -0.26) ***  -0.15 (-0.38, 0.08)  **-0.40 (-0.58, -0.21)*** |  |

Notes: ES (#), number of effect sizes; Participants (#), total number of participants nested within effect sizes; $\bar{X}$ $\bar{\times}$(95% CI), mean and 95% confidence intervals; BDI, Beck Depression Inventory; CES-D, Center for Epidemiologic Studies Depression Scale; FIQ, Fibromyalgia Impact Scale for depression; AIMS, Arthritis Impact and Measurement Scale for depression; MHI, Mental Health Inventory; POMS, Profile of Moods States; DASS, Depression, Anxiety and Stress Scale; VAS, Visual Analog Scale; HAD, Hospital Anxiety and Depression Scale; AORC, arthritis and other rheumatic conditions; OA, osteoarthritis; RA, rheumatoid arthritis; FM, fibromyalgia; SLE, systemic lupus erythematosus; * statistically significant within-group changes (non-overlapping 95% CI).
