# Supplementary material for: Effects of exercise on depression in adults with arthritis: a systematic review with meta-analysis of randomized controlled trials
Source: Arthritis Res Ther. 2015 Feb 3;17(1):21. doi: 10.1186/s13075-015-0533-5 (PMC4467075; doi:10.1186/s13075-015-0533-5)
Supplement: Additional file 5: — Mixed effects meta-regression results for changes in depressive symptoms. This file provides a table of all meta-regression analyses conducted. [file 13075_2015_533_MOESM5_ESM.docx]

Additional File 5. Mixed effects meta-regression results for changes in depressive symptoms.

|  |  |  |  | |  | |  |
| --- | --- | --- | --- | --- | --- | --- | --- |
| Variable | Studies  (#) | ES  (#) | Participants  (#) | $\beta$_1_ + SE | | CI (95%) |  |
| *Study Characteristics* |  |  |  |  | |  |  |
| - Year of publication | 29 | 35 | 2499 | 0.011 + 0.015 | | -0.018, 0.040 |  |
| - Dropouts (%) | 26 | 32 | 1422 | -0.004 + 0.007 | | -0.009, 0.018 |  |
| *Participant Characteristics* |  |  |  |  | |  |  |
| - Age (yrs)  - Symptom Duration (yrs)  - Diagnosis duration (yrs)  - Body weight changes (kg)  - BMI changes (kg^.^m^2^)  - Body fat changes (%)  - Physical function changes (*g*)  - Pain changes (*g*)  - Quality-of-life changes (*g*)  - Anxiety changes (*g*)  - VO_2max_ changes (ml^.^kg^.-1^min^-1^)  - Upper body strength changes (*g)*  - Lower body strength changes *(g)*  - Balance changes (*g)* | 28  12  8  3  5  3  21  25  18  13  7  6  9  3 | 33  14  10  3  5  3  26  30  21  16  10  9  10  3 | 2353  652  521  226  266  121  1513  1971  1276  976  590  530  584  147 | 0.014 + 0.009  0.016 + 0.036  -0.045 + 0.060  -0.158 + 0.093  -1.583 + 0.304  1.282 +1.770  -0.269 + 0.287  0.355 + 0.125  -0.674 + 0.157  0.539 + 0.290  -0.182 + 0.124  -0.473 + 0.422  -0.299 + 0.253  1.497 + 0.730 | | -0.004, 0.031  -0.054, 0.087  -0.161, 0.071  -0.340, 0.023  **-2.180, -0.986***  -2.188, 4.752  -0.832, 0.294  **0.110, 0.601***  **-0.982, -0.366***  -0.030, 1.108  -0.425, 0.060  -1.301, 0.354  -0.794, 0.196  **0.067, 2.927*** |  |
| *Exercise Characteristics*  - Length (weeks)  - Frequency (days/week)  - Duration (minutes/session)  - Compliance (%)  - Minutes/week  - Minutes/week (adj.)  - Total minutes  - Total minutes (adj.) | 29  27  18  15  24  15  24  15 | 35  33  24  20  30  18  30  20 | 2499  2340  1674  1393  2100  1393  2100  1393 | 0.003 + 0.005  -0.034 + 0.044  0.003 + 0.009  0.0005 + 0.007  -0.001 + 0.001  -0.001 + 0.002  0.00001 + 0.00004  0.00000 + 0.00006 | | -0.006, 0.013  -0.120, 0.051  -0.014, 0.020  -0.015, 0.013  -0.004, 0.002  -0.005, 0.002  -0.00007, 0.00009  -0.0001, 0.0001 |  |

Notes: Notes: ES(#), number of effect sizes; Participants (#), number of participants nested within effect sizes; yrs, years; adj, adjusted for compliance; kg, kilograms; kg^.^m^2^ , kilogram meters squared; *g*, standardized mean difference effect size, adjusted for compliance; VO_2max,_ maximum oxygen consumption in ml^.^kg^.-1^min^-1^; $\beta$_1_ + SE, slope + standard error; CI (95%), 95% confidence intervals; (adj), adjusted for compliance; *, statistically significant, non-overlapping 95% confidence intervals; **Boldfaced** items indicate statistical significance.
